# Borylation Directed Borylation of Indoles Using Pyrazabole Electrophiles: A One‐Pot Route to C7‐Borylated‐Indolines

**DOI:** 10.1002/anie.202206230

**Published:** 2022-06-28

**Authors:** Jürgen Pahl, Emily Noone, Marina Uzelac, Kang Yuan, Michael J. Ingleson

**Affiliations:** ^1^ School of Chemistry University of Edinburgh Edinburgh EH9 3FJ UK

**Keywords:** Boranes, C−H Borylation, Electrophilic Substitution, Indoles, Transient Directing Group

## Abstract

Pyrazabole (**1**) is a readily accessible diboron compound that can be transformed into ditopic electrophiles. In **1** (and derivatives), the B⋅⋅⋅B separation is ca. 3 Å, appropriate for one boron centre bonding to N and one to the C7 of indoles and indolines. This suitable B⋅⋅⋅B separation enables double E−H (E=N/C) functionalisation of indoles and indolines. Specifically, the activation of **1** with HNTf_2_ generates an electrophile that transforms N−H indoles and indolines into N/C7‐diborylated indolines, with N−H borylation directing subsequent C7−H borylation. Indole reduction to indoline occurs before C−H borylation and our studies indicate this proceeds via hydroboration—C3‐protodeboronation to produce an intermediate that then undergoes C7 borylation. The borylated products can be converted in situ into C7‐BPin‐N‐H‐indolines. Overall, this represents a transient directed C−H borylation to form useful C7‐BPin‐indolines.

## Graphical abstract

Electrophiles based on pyrazabole are simple to access reagents for the double E−H (E=N/C) borylation of N−H‐indoles/indolines. Due to the robust B_2_N_4_ core of pyrazaboles and a B⋅⋅⋅B separation of ca. 3 Å, the first borylation directs the second to a proximal C−H. For N−H‐indoles, C7‐borylation occurs alongside reduction resulting, post work‐up, in the one‐pot formation of C7‐BPin‐indolines without requiring installation of a directing group.

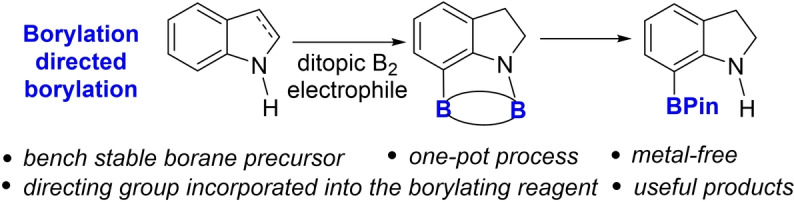

Organoboranes are ubiquitous in modern synthesis in part due to the power of the Suzuki–Miyaura reaction.^[1]^ Therefore there is a continued impetus to discover improved routes to known organoboranes and methods to form novel organoboranes. One efficient method to form organoboranes is by C−H borylation, with iridium‐catalysed borylation methodologies particularly powerful.^[2]^ Ir‐catalysed borylation proceeds under steric control,^[2c]^ while electrophilic C−H borylation is a precious metal free approach that is controlled by (hetero)arene electronics.^[3]^ Directed C−H borylation (metal catalysed and metal‐free) is an established method to borylate C−H positions otherwise challenging to functionalise,^[4]^ e.g. the C7 position in indoles and indolines.^[5]^ Directed C−H borylation most often proceeds by formation of a bond between the substrate and the catalyst (or the electrophilic borane), with the directing group either part of the substrate (e.g. the basic N in pyridines) or installed in a separate step (e.g. by N−H functionalisation). However, the latter (Figure 1, top) is step inefficient. Transient directing groups are more attractive as these are installed, direct C−H borylation and are removed all in one‐pot.^[6]^ Significant advances in metal‐catalysed transient directed C−H borylation have been reported.^[7]^ Notably, the transient directing group approach has not been applied for the C7 borylation of indoles/indolines, while its use in electrophilic C−H borylation is significantly underdeveloped.^[8]^

**Figure 1:**
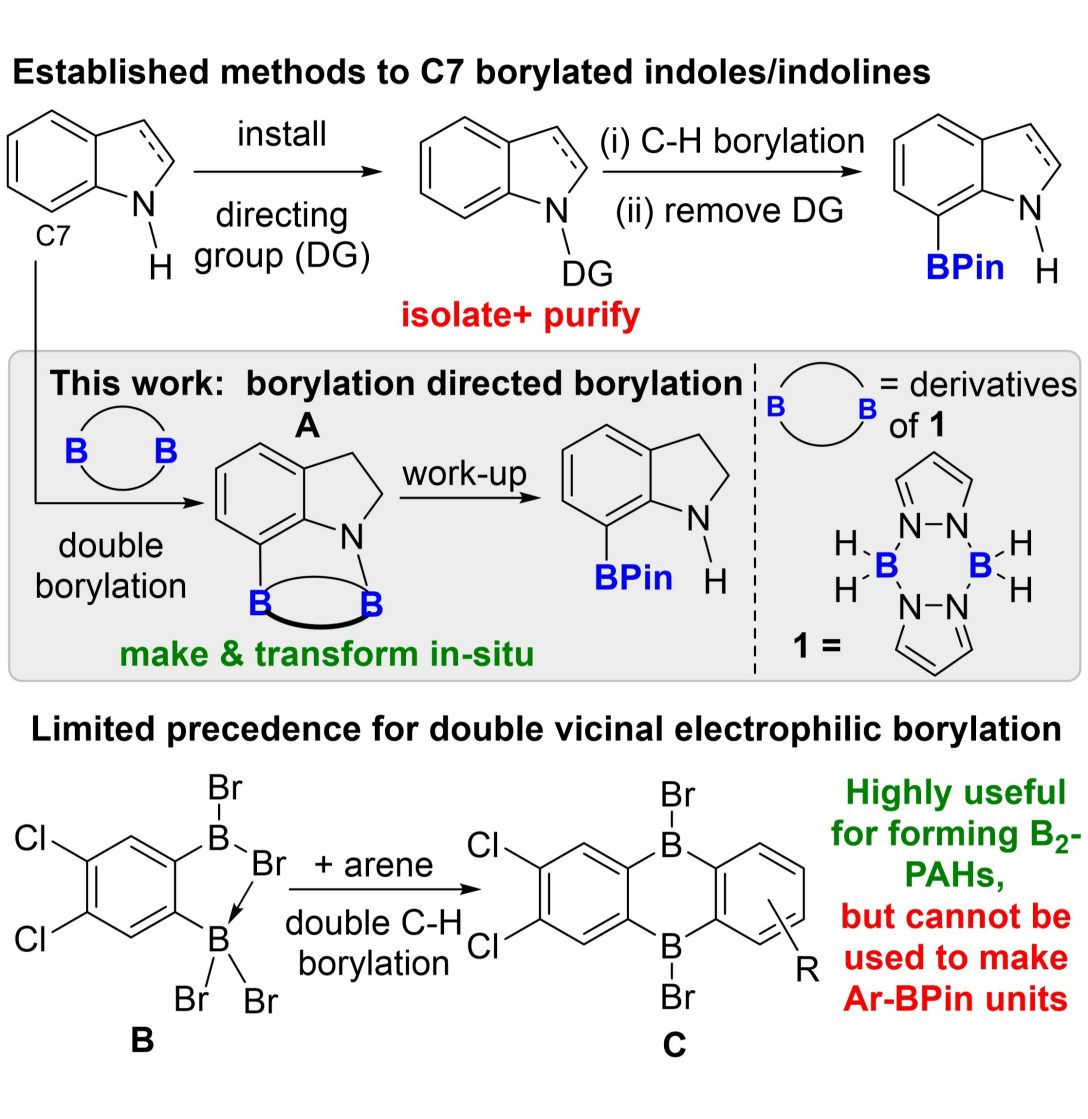
Top: Established routes to achieve C7 borylation using a pre‐installed directing group. Middle: This work, borylation directed borylation. Bottom: A rare example of a diboron ditopic electrophile used in vicinal double C−H borylation.

Conceptually, if two electrophilic boron centres present in a single molecule are separated by an appropriate distance then one boron centre can act as a directing group by borylating the most reactive E−H site (E=C or N). This will position the second boron centre appropriately to transform a C−H into a C−B unit at a site otherwise hard to borylate (e.g. to form **A**, Figure 1 middle). Deliberate cleavage of the more reactive E−B bond, e.g. by selective protodeboronation during work up,^[9]^ then would leave only the desired C−B bond. In this process, that we term borylation directed borylation, the first borylation step fulfils the role of a transient directing group. Notably, this method is distinct to the more common traceless directing group approach which installs the directing group onto the substrate before adding a separate reagent/catalyst to effect C−H functionalisation.^[6]^ In borylation directed borylation the directing group is embedded into the borylating reagent. However, generating suitably structured ditopic B_2_‐electrophiles that are: i) sufficiently reactive for double electrophilic borylation, and ii) can be transformed into synthetically useful boron species post borylation is challenging. One notable report on addressing the first point comes from Wagner et al. using **B** (Figure 1, bottom) which possesses a B⋅⋅⋅B separation of ca. 3 Å,^[10]^ to effect double vicinal C−H borylation of aromatics.^[11]^ While a powerful method for accessing B_2_‐containing polycyclic aromatic hydrocarbons (e.g. **C**), double electrophilic borylation using **B** has not been combined with further transformations to form organoboranes ubiquitous in synthesis (e.g. pinacol boronate esters (BPin)). Thus, identifying readily accessible ditopic diboron electrophiles able to perform double E−H borylation and then be transformed readily into synthetically useful boron units is an unmet challenge. If realised this would streamline the synthesis of desirable borylated (hetero)arenes.

Pyrazabole, **1**, is an attractive precursor to ditopic diboron‐electrophiles^[12]^ as it: i) can be readily synthesised from inexpensive materials (pyrazole and L‐BH_3_);^[13]^ ii) is bench stable;^[13]^ iii) undergoes facile activation of B−H bonds to generate diboron‐electrophiles.^[14]^ Furthermore, pyrazaboles have a relatively flexible B_2_N_4_ core (able to adopt planar, chair and boat conformations)^[15]^ and a B⋅⋅⋅B separation of ca. 3 Å,^[16]^ comparable to the B⋅⋅⋅B separation expected in N/C7 or C3/C4 diborylated indolines/indoles.^[17]^ This indicates that the double E−H borylation products, e.g. the polycyclic species **A** (Figure 1), will not be significantly strained thus should be accessible. Herein, we report that pyrazabole derived electrophiles enable the unprecedented transformation of N−H‐indoles and indolines into C7‐borylated‐indolines by borylation directed borylation.

Due to the precedence in C−B bond formation using boron electrophiles derived from L‐BR_2_H/HNTf_2_ combinations (L for example=*N‐*heterocyclic carbenes, HNTf_2_=bis(trifluoromethane sulfonyl)amine)^[18]^ our investigations started by activating **1** with commercially available HNTf_2_. The addition of HNTf_2_ to **1** resulted in immediate H_2_ evolution and crystals suitable for X‐ray diffraction studies were formed directly from the reaction mixture. The solid‐state structure of [Tf_2_N(H)B(pyrazole)]_2_, **2** (Figure 2), contained a *trans* arangement of the NTf_2_ units and an almost planar B_2_N_4_ ring with the two boron atoms being 0.128(3) Å out of the N_4_‐plane. The B⋅⋅⋅B separation in **2** is in the expected range at 3.126(2) Å, while the B‐NTf_2_ distance at 1.609(2) Å is unremarkable for a B‐NTf_2_ unit,^[19]^ Compound **2** could be isolated in good (74 %) yield on multigram scale using two equiv. of HNTf_2_ with respect to **1**. While **2** is poorly soluble (in halocarbon solvents) it displays a *δ* ${}_{{}^{11}B}$ =−3 (Figure S6), shifted downfield from that of **1** (*δ* ${}_{{}^{11}B}$ =−8.3, Figure S4). Attempts to isolate a mono‐NTf_2_ derivative, compound **3**, by using equimolar HNTf_2_ and **1** led to broad ^1^H NMR spectra and precipitation of **2** on standing, with 0.5 equiv of **1** left unreacted in solution. NMR spectra from combining equimolar mixtures of **1** and **2** suggest an exchange process occurs as new broad ^1^H resonances not corresponding to **1** or **2** are observed (Figure S14–S18) along with new ^11^B resonances at −1.8 and −4.5 ppm (consistent with a lower symmetry pyrazabole such as **3**). Thus, the mono‐NTf_2_ species **3**, appears accessible in solution although only **1** and **2** can be isolated as crystalline solids in our hands.

**Figure 2:**
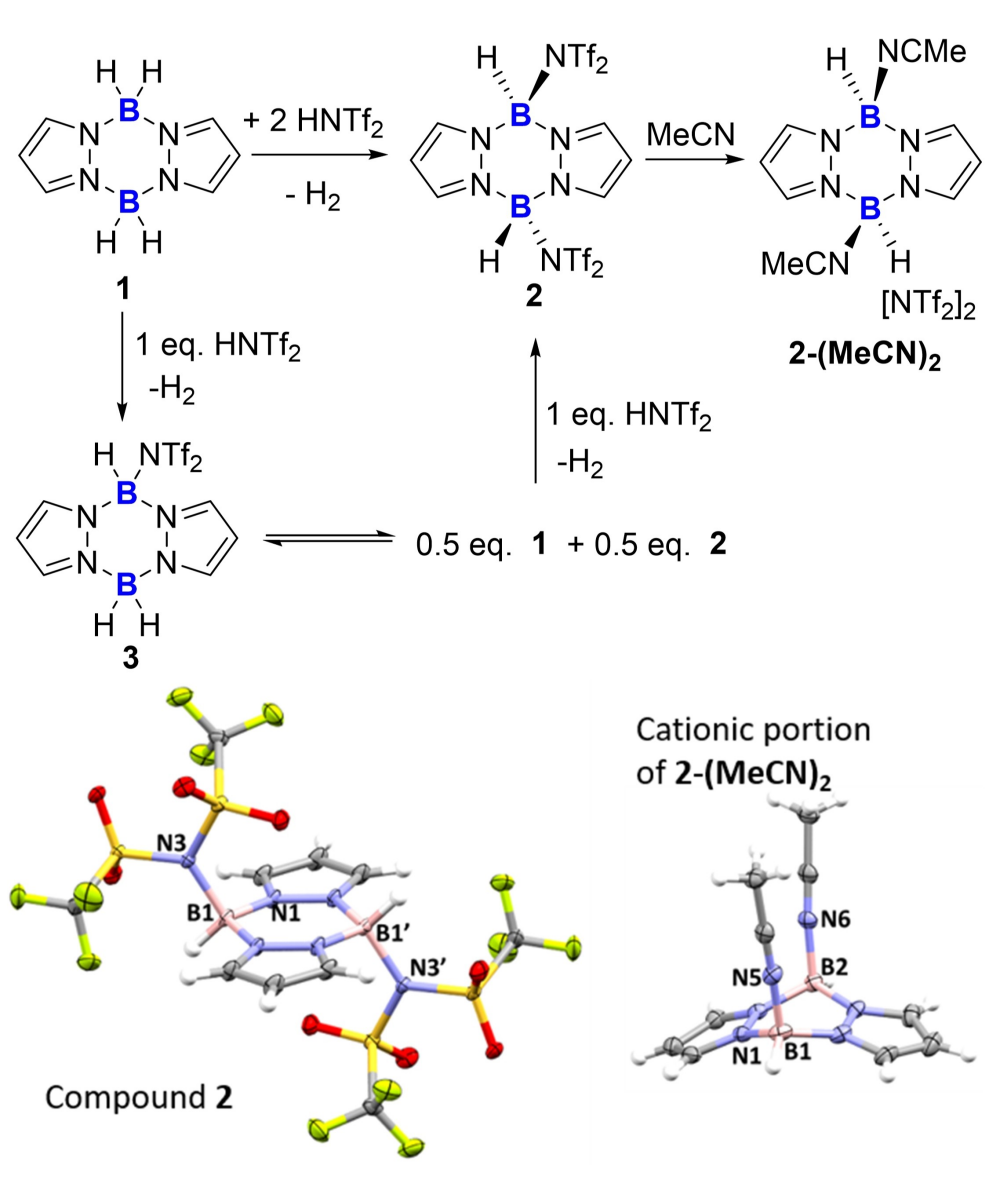
Formation of **2**,**2‐(MeCN)_2_** and **3** and bottom the solid‐state structures of **2** and the cationic portion of **2‐(MeCN)_2_** (ellipsoids at 50 % probability). Selected metrics [Å] for **2**: B1⋅⋅⋅B1′ 3.126(2), B1−N3 1.609(2), B1−N1 1.532(2). For **2‐(MeCN)_2_**: B1⋅⋅⋅B2 2.974(4), N1−B1 1.530(4), B1−N5 1.565(4), B2−N6 1.569(4).

Compound **2** is a ditopic electrophile, for example the addition of excess MeCN to **2** leads to displacement of both NTf_2_ anions and formation of the salt **2‐(MeCN)_2_**. This crystallises as the *cis*‐isomer with both MeCN molecules on one face. Therefore, the formation of **2** does not lock a *trans* arrangement, important as a *cis*‐configuration is essential for borylation directed borylation. Note, the displacement of [NTf_2_]^−^ by MeCN indicates that **2** and **3** will be reactive borenium equivalents.^[20]^ Assessment of the Lewis acidity of the borenium **[3]^+^** (Scheme 1) towards a soft nucleophile (H^−^) using the hydride affinity relative to BEt_3_ methodology,^[21]^ revealed that **[3]^+^** is indeed a strong electrophile. In fact, **[3]^+^** has a hydride affinity greater than the [(catecholato)B(NEt_3_)]^+^ borenium cation (Δ*H*=−43.6 kcal mol^−1^),^[18]^ which is effective in heteroarene C−H borylation.^[22]^ Thus **3** should be sufficiently electrophilic to react with activated heteroarenes.

**Scheme 1:**
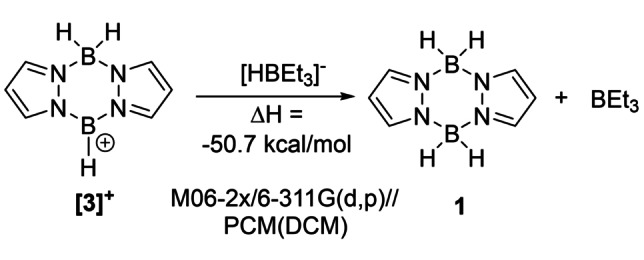
The hydride affinity (relative to BEt_3_) of **[3]^+^**.

With electrophilic pyrazabole derivatives accessible the functionalisation of indoles was explored targeting double E−H borylation (C3/C4 or N/C7). Work up using pinacol/base would then form mono‐BPin indoles by selective protodeboronation of the more reactive E−B bond (C3−B or N−B).[^7^, ^23^] Pyrazabole‐NTf_2_ electrophiles do indeed effect double borylation of N−H‐indoles, with it selective for N and C7−H borylation. However, this proceeded alongside the reduction of the C2=C3 unit, leading to formation of a C7‐borylated indoline. The initial product was converted in situ to the pinacol boronate ester **4 a** (Figure 3). **4 a** could be isolated in 78 % in a one‐pot process starting from N−H indole using 1.1 equiv of **“3”** (this refers to the mixture formed in situ from combining 0.55 equiv of **1** and 0.55 equiv of **2** and is labelled as **3** herein) via a simple work up (addition of pinacol/K_2_CO_3(aq)_). In contrast, combining **2** and N−H indole led to no C7 borylation (significant **2** remained even after 18 h at 100 °C). However, the combination of a hindered base, **2** and N−H indole did lead (on heating) to C2−C3 reduction and borylation at N and C7 to form an analogous C7‐borylated indoline (see below). C7‐functionalised indolines are of significant interest, for example as anti‐cancer and anti‐inflammatory agents.^[24]^ While directed borylation using *N*‐pivaloyl‐indoline and BBr_3_ also leads to C7‐borylated indolines,^[5c]^ this is step inefficient as it requires separate installation and subsequent removal of the pivaloyl directing group. Furthermore, removal of N‐pivaloyl from indolines requires forcing conditions (e.g. heating with strong base or acid),^[25]^ that would be incompatible with C‐BPin units. As many N−H‐indoles are commercially available and inexpensive this indole reduction/C7‐borylation process is an attractive route to form desirable C7‐BPin indolines.

**Figure 3:**
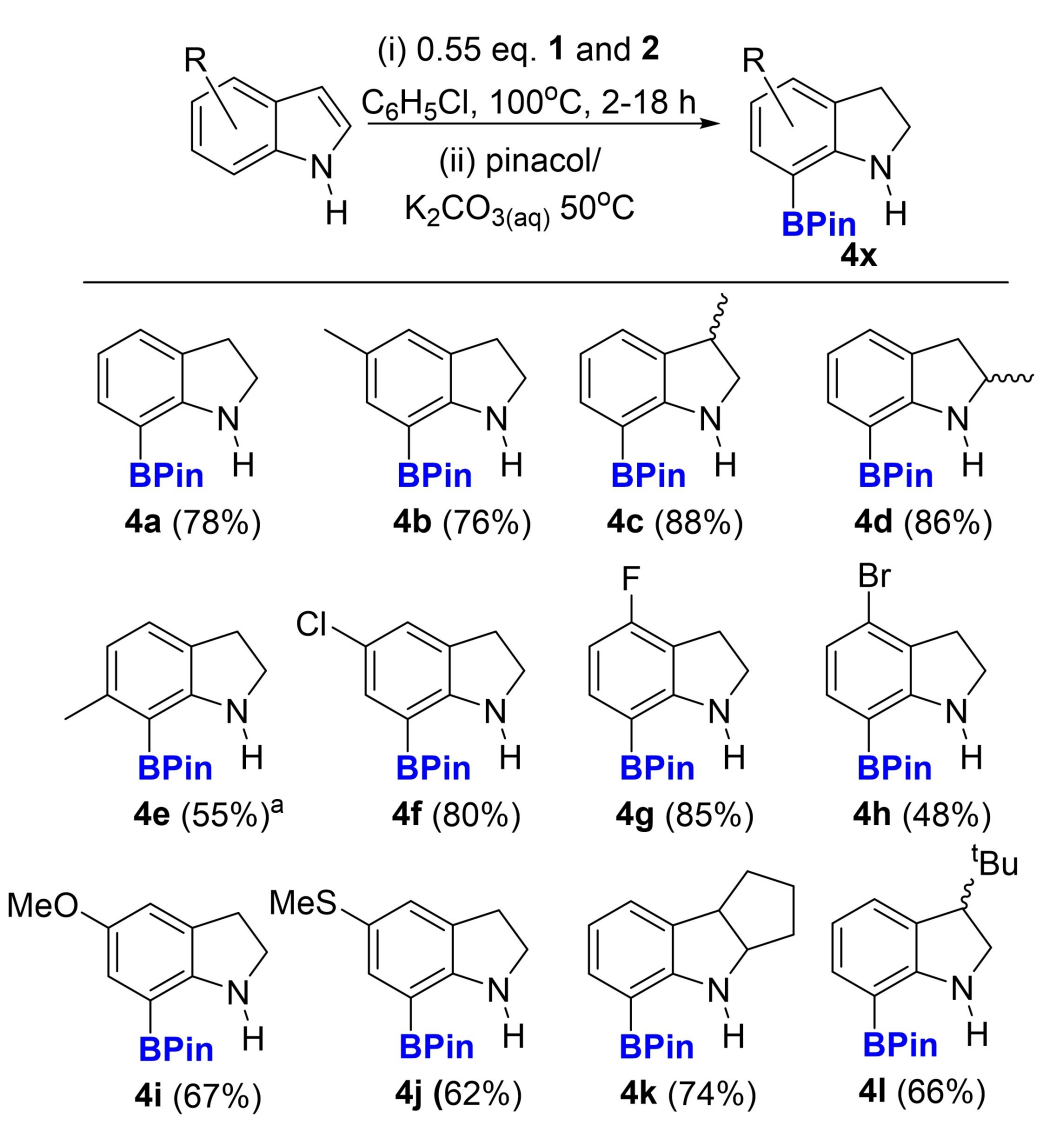
Scoping of N−H‐indole reduction/C7‐borylation using **3** (combining equimolar **1**+**2**). *a*=Pinacol installation requires heating at 80 °C.

Scoping studies (as using **3** is more atom efficient than using **2**/hindered base only **3** is utilised in the scoping) revealed reduction/C7‐borylation to be compatible with substituents at indole positions C2−C6, although C6 substitution (**4 e**) slows pinacol installation meaning higher temperatures are required for this step. The transformation was compatible with N−H‐indoles mono‐substituted with methyl (**4 b**–**4 e**), halide (**4 f**–**h**), OMe (**4 i**), SMe (**4 j**), a C2/C3 disubstituted indole (**4 k**) and even with a ^t^Bu group at the C3 position (**4 l**). Furthermore, the majority of BPin products can be isolated in good purity (>95 %) without column chromatography. From these studies the functional group tolerance is comparable in scope to other electrophilic C−H borylation reactions using borocations (or functional equivalents of borocations), although groups prone to hydroboration (e.g. esters) are not compatible.^[22]^

The mechanism leading to C7 borylated indolines also was explored, with the starting pyrazabole electrophile assumed to be **3** given the absence of any N−H‐indole borylation using **2** without an exogenous base (e.g. under Figure 3 conditions). Studies from Fontaine et al. indicated that N−H‐indole reduction with L‐BH_3_ can proceed by C2−C3 hydroboration and subsequent C3‐protodeboronation (Scheme 2).^[26]^ In our work a related process involving hydroboration/C3‐protodeboronation is also feasible, which could then be followed by directed C7−H borylation.

**Scheme 2:**
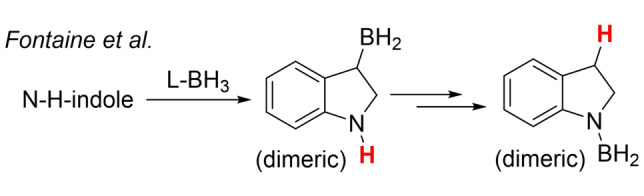
L‐BH_3_ reduction of indoles.

In situ NMR spectroscopy studies of N−H‐indole borylation using **3** revealed multiple indoline intermediates. For facilitating their identification the borylation of N−H‐indole and 5‐Cl‐N−H‐indole with **3** was monitored in situ (Figures S56–S66). The major intermediates formed at room temperature displayed NMR data consistent with hydroboration and binding of the second boron at N (e.g. **5‐H/5‐Cl** Figure 4), this included diastereotopic proton resonances consistent with a chiral indoline. Further reaction proceeded though a species consistent with **6‐H/6‐Cl** (e.g. two indoline CH_2_ units appearing as triplets in the ^1^H NMR spectrum, Figure S58). Heating to 100 °C consumed mixtures of **5‐H(Cl)/6‐H(Cl)** with formation of one new product in each case. These displayed resonances consistent with **7‐H** (**7‐H** was also crystallographically characterised, see below) and **7‐Cl** (Figure S66–S77) and these are the major products formed at the end of the reaction. Based on this data, the precedence from Fontaine and co‐workers and subsequent reactions using N−Me‐indole and N−H‐indoline as substrates (see below) we propose the mechanism shown in Figure 4. An alternative process to form **6** proceeding via **D** (formed by N−B bond formation and tautomerisation) is feasible but is disfavoured by the absence of diagnostic iminium C2‐H resonances (by ^1^H and ^13^C{^1^H} NMR spectroscopy).^[27]^

**Figure 4:**
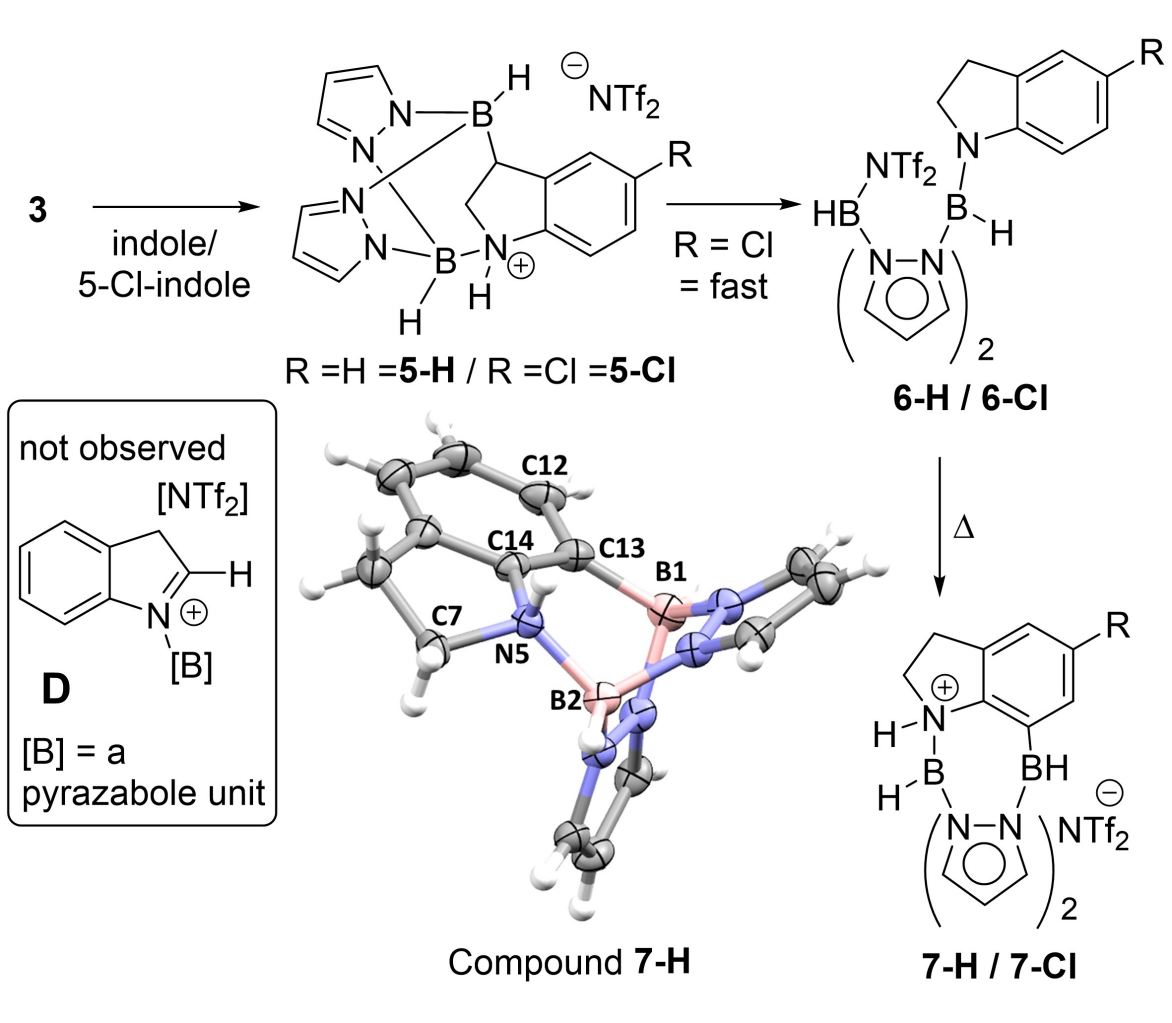
Reduction/C7‐borylation to form **7‐H/7‐Cl** proceeding via **5‐H/5‐Cl** and **6‐H/6‐Cl**. Note an alternative isomer of **6** (from exchanging H/NTf_2_ on the boron centres is also feasible (the ^11^B NMR resonances are broad thus multiplicity from B−H coupling is not observed)). Bottom: Structure of the cationic portion of **7‐H** (ellipsoids at 50 % probability). Selected bond lengths [Å] and angles [°]: B1−C13 1.602(4), B2−N5 1.578(3), B1⋅⋅⋅B2 2.858(4); C7‐N5‐C14 103.2(2), C14‐N5‐B2 119.2(2), B2‐N5‐C7 116.4(2).

Regarding the solid‐state structure of **7‐H** (Figure 4) this confirmed the product is reduced at C2/C3 and contains a single pyrazabole unit that has borylated the N and C7 positions, with the indoline N (N5) quaternized by protonation (Σ C7‐N5‐C14, B2‐N5‐C7 and B2‐N5‐C14 339.6°). One notable point regarding the structure is the lack of significant strain in the system, for example the B1‐C13‐C12 and B1‐C13‐C14 angles are comparable (122.2(6)° and 123.6(2)°) and the B⋅⋅⋅B distance (2.858(4) Å) is close to the B⋅⋅⋅B distance in **1** (and **2**). This is consistent with our hypothesis that pyrazaboles are appropriate diboron scaffolds for indole/indoline double borylation at N/C7 (and presumably C3/C4). Electrophilic C−H borylation requires a base to facilitate deprotonation of arenium species (via a step‐wise or concerted S_E_Ar mechanism).[^20^, ^22^, ^28^] The structure of **7‐H** shows the indoline‐N has acted as the Brønsted base and is thus chiral (leading to diasterotopic resonances in **7‐H**). Thus addition of an exogenous base deprotonates the N−H unit of **7‐H** simplifying ^1^H NMR spectra (to two triplets observed for the CH_2_ units, Figure S78).

Regarding the mechanism for forming C7‐borylated indolines from **2**/N−H‐indole in the presence of the hindered base, 2,6‐^t^Bu‐4‐Me‐pyridine (DBP, used to preclude _base_N→B dative bond formation), we propose this proceeds via a different sequence, starting with rapid _indole_N−B bond formation. This was indicated by isolation of **8** (Figure 5) from a room temperature reaction of **2** with N−H‐indole in the presence of DBP (with [DBP‐H][NTf_2_] observed as the by‐product by NMR spectroscopy). The formation of **8** indicates that pyrazabole‐NTf_2_ electrophiles actually react with N−H‐indoles at N in preference to C3 as no C3 borylation was observed under these conditions. However, in the absence of an exogenous base (as per the conditions in Figure 3) we propose the initial _indole_N(H)−B_pyrazabole_ interaction is reversible and this ultimately leads to formation of **5** from combining N−H‐indoles and **3**. Thus when using an exogenous base/**2**, reduction/C7‐borylation still proceeds (see Figure S79), but it is via a different sequence of steps to that using **3**/no exogenous base.

**Figure 5:**
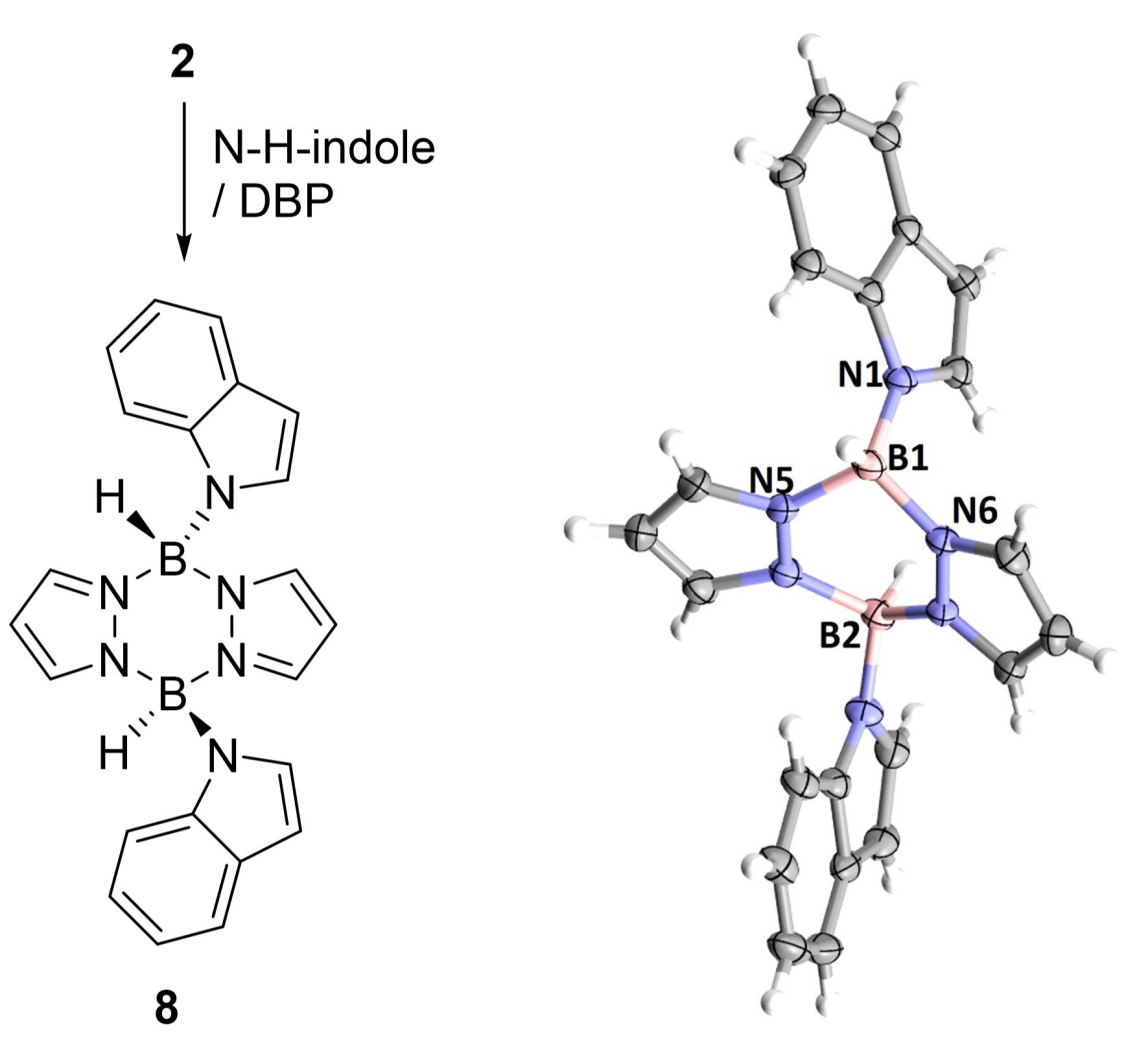
Formation of **8**, ellipsoids at 50 % probability. Selected bond lengths [Å]: B1⋅⋅⋅B2 3.071(2), N−B1 1.551(2), N5−B1 1.517(2), N6−B2 1.498(2).

Returning to the more atom efficient borylation method involving using just **3**, if the mechanism in Figure 4 is correct then N−H‐indoline should undergo C7‐borylation when treated with equimolar **2**/hindered base. The treatment of N−H‐indoline with one equiv. **2** and DBP rapidly forms the N‐borylation product **6‐H**, as the major species and then **7‐H** (slowly at room temperature, Figure S86). Heating leads to faster consumption of **6‐H** with **7‐H** formed as the major product (Scheme 3 and Figure S88). The identical NMR spectra for the intermediate assigned as **6‐H** starting from both N−H‐indole and N−H‐indoline confirms reduction precedes C7‐borylation starting from N−H‐indole/**3**. Furthermore, it indicates that the formation of C7‐borylated indolines can use N−H‐indole (with **3**/no base or **2**/base) or N−H‐indoline (with **2/**base) starting materials. Note, when using N−H indoles in the absence of exogenous base the C3‐B unit (formed by indole hydroboration) can be viewed as acting as the Brønsted base for the N−H borylation step (as observed by Fontaine, Scheme 1).

**Scheme 3:**
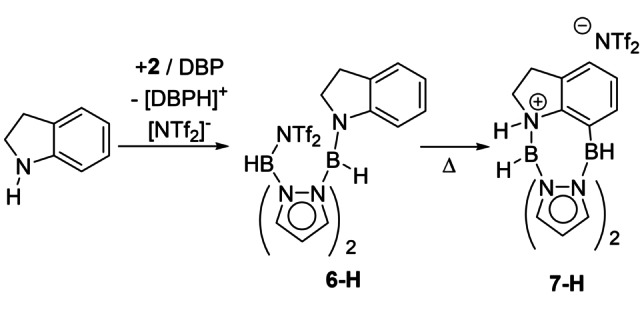
Formation of **7‐H** from N−H‐indoline via **6‐H**.

To further support the initial hydroboration step (e.g. to form **5**, Figure 4), N−Me‐indole was used in place of N−H‐indole as the N−Me group would disfavour any further reactivity such as the acidic N−H unit in **5** reacting with the B−C3 unit by protodeboronation to form **6** (Figure 4). The addition of **3** to N−Me‐indole results in the formation of **9** (Figure 6) as the major product at room temperature, which could be isolated in 87 % yield. Compound **9** is the product from *N*‐Me‐indole hydroboration with **3**, with one boron bonded to C3 and the second boron centre coordinating to the indoline nitrogen. The ^1^H NMR spectra for **9** contains three diastereotopic resonances for the indoline unit as expected (Figure S93). It should be noted that the unactivated pyrazabole **1** does not react with N−H‐indole or N−Me‐indole (even on heating). In addition, attempts to hydroborate N−Me‐indole with (IMe)BH_2_NTf_2_ also led to no reaction at room temperature, suggesting that the facile hydroboration observed on combining **3** and N−Me‐indole maybe due to the bis‐hydroborane structure of electrophilic **3**.

**Figure 6:**
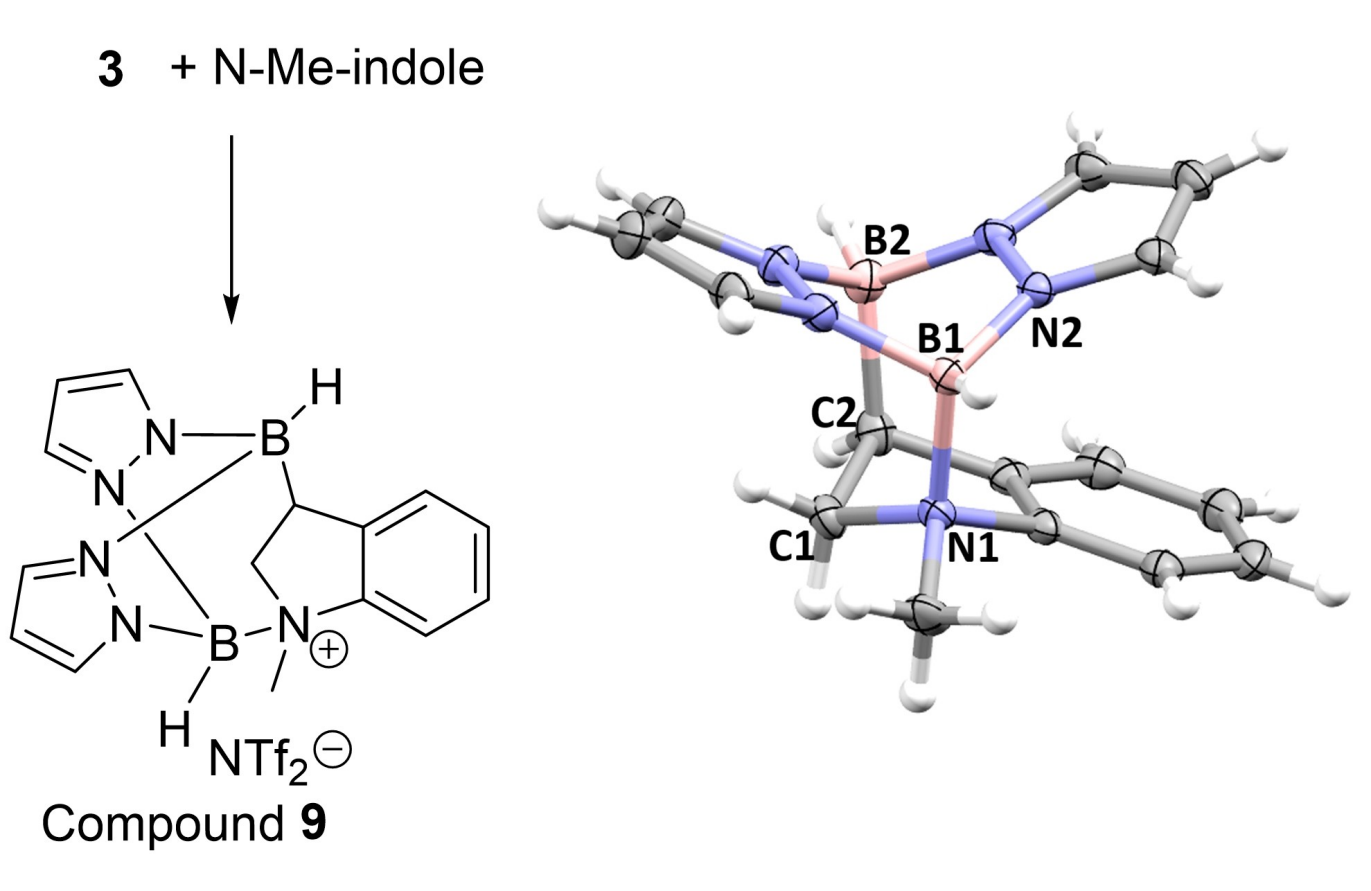
Hydroboration of N−Me‐indole with **3** to form **9**. Right: Solid‐state structure of the cationic portion of **9** (ellipsoids at 50 % probability). Selected bond lengths and angles [Å/°]: B1⋅⋅⋅B2 2.829(3), B1−N1 1.664(2), B1−N2 1.532(2), B2−C2 1.660(2); B1‐N1‐C1 112.4(1), B2‐C2‐C1 112.1(1).

While the B⋅⋅⋅B separation in **9** is in the expected range, the B1‐N1 bond in **9** is significantly longer than the B−NTf_2_ bonds in **2** and the B2‐N5 distance in **7‐H**, however it is comparable to the B−N distance in the Lewis adduct between N−H‐indoline and B(C_6_F_5_)_3_ (B−N 1.650(2) Å).^[27]^ To investigate if any dissociation of the B−N_indoline_ dative bond occurs **9** was heated with or without a hindered base present (targeting borylation at C4). This was unsuccessful leading to extremely slow reactivity at 100 °C (Figure S99), indeed **9** remains the major product even after 14 days at 100 °C) while forming multiple intractable products at higher temperatures (160 °C). Finally, *N*‐SiR_3_‐indoles were explored as the larger R_3_Si groups may disfavour boron coordination at N, e.g. in the R_3_Si analogues of **9**. However, combining **3** with N‐TMS‐indole led to the hydroborated product **10** (Scheme 4) as the major species. Compound **10** forms at room temperature and while it frustrated isolation in our hands its assignment is based on its closely related NMR spectra to **9** (Figure S104). Heating of mixtures containing the hydroborated species **10** led to the formation of the C7‐borylated indoline product **7‐H** as the major product (see Figure S105, S106). Using N‐TIPS‐indole (TIPS=triisopropylsilyl) also led on heating to the formation of **7‐H**, however in this case no hydroboration intermediate (e.g. **11**) was observed. The major silane by‐product observed by NMR spectroscopy was R_3_SiH in both cases, presumably formed by abstraction of a hydride from a four coordinate at boron B−H unit of a pyrazabole species by a silicon electrophile, with related processes well‐documented to occur.^[29]^ Regardless of the exact mechanism leading from N‐R_3_Si‐indole/**3** to **7‐H**, the fact that reduction/C7 borylation proceeds even using the large TIPS protecting group has to date frustrated our attempts to direct reactivity towards C3/C4 diborylation.

**Scheme 4:**
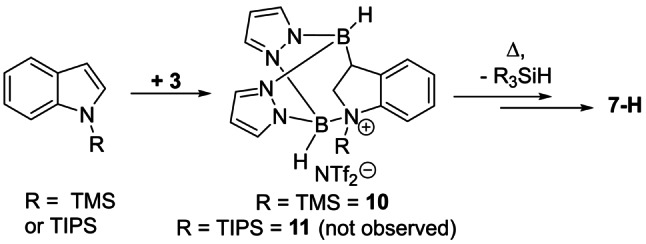
Formation of **7‐H** from N‐SiR_3_‐indoles.

In conclusion, pyrazabole derived electrophiles are readily accessible reagents for converting N−H‐indoles into C7‐borylated indolines. The diboron core of pyrazabole, with a B⋅⋅⋅B separation of ca. 3 Å, is key for enabling the formation of two bonds from one pyrazabole unit to one indole/indoline. Importantly, while the B_2_N_4_ pyrazabole core is sufficiently robust to persist during electrophilic borylation (enabling borylation directed borylation), the B_2_N_4_ pyrazabole core is reactive enough that it can be converted into synthetically ubiquitous organoboron reagents (pinacol boronate esters) via a simple work‐up. This balance between stability of the diboron core during E−H borylation and reactivity during work‐up is essential to access the synthetic desirable organoboron products. Work is ongoing in our laboratory using pyrazabole derived electrophiles and other ditopic diboron compounds to expand the borylation directed borylation approach.

## Conflict of interest

The authors declare no conflict of interest.

## Supporting information

As a service to our authors and readers, this journal provides supporting information supplied by the authors. Such materials are peer reviewed and may be re‐organized for online delivery, but are not copy‐edited or typeset. Technical support issues arising from supporting information (other than missing files) should be addressed to the authors.

Supporting Information

Supporting Information

Supporting Information

Supporting Information

Supporting Information

Supporting Information

## Data Availability

The data that support the findings of this study are available in the Supporting Information of this article.
