# Supplementary material for: Borylation Directed Borylation of Indoles Using Pyrazabole Electrophiles: A One‐Pot Route to C7‐Borylated‐Indolines
Source: Angew Chem Int Ed Engl. 2022 Jun 28;61(32):e202206230. doi: 10.1002/anie.202206230 (PMC9401042; doi:10.1002/anie.202206230)
Supplement: Supplementary file 6 — Supporting Information [file ANIE-61-0-s003.pdf]

Angewandte Chemie
Eine Zeitschrift der Gesellschaft Deutscher Chemiker

Supporting Information

Borylation Directed Borylation of Indoles Using Pyrazabole Electrophiles: A One-Pot Route to C7-Borylated-Indolines

*J. Pahl, E. Noone, M. Uzelac, K. Yuan, M. J. Ingleson**

Table of Contents

1.0 General Considerations 3

2.0 Synthesis and Activation of Pyrazaboles 4

2.1 Synthesis of Pyrazabole (1) 4

2.2 Synthesis of *bis*-NTf_2 Pyrazabole (2) 4

2.3 Synthesis of *bis*-MeCN Pyrazabole 2-$(MeCN)_2$ 5

2.4 Formation of *mono*-NTf_2 Pyrazabole (3) *in situ* 5

2.5 NMR Spectra of Synthesised Pyrazaboles 6

3.0 Directed C7-Borylation of N-H Indoles 16

3.1 General Procedure 1 16

3.2 Substrate Scope and Characterisation of 7-BPin Indolines (4a-k) 16

3.3 NMR Spectra of Synthesised 7-BPin Indolines 23

4.0 Mechanistic Studies 42

4.1 Reaction Profiling with Indole and 5-Cl Indole 42

4.2 Characterisation of C7-Borylated Intermediates 7-H / 7-Cl 48

4.3 Addition of Exogenous Base to C7-Borylayed Intermediate 7-H 56

4.4 Reactivity in the presence of a hindered base 57

4.5 Reaction of N-H Indoline and 2 in the Presence of a Exogenous Base 63

5.0 Reactivity of N-R Indoles with Pyrazaboles 67

5.1 Reactivity with N-Me Indole 67

5.3 Synthesis of N-TMS Indole 73

5.2 Synthesis of N-TIPS Indole 74

5.4 Hydroboration of N-TMS Indole to 10 75

5.6 C7-Borylation of N-SiR_3 Indole to form 7-H 76

6.0 Computational Data 78

7.0 Crystallographic Data 82

8.0 References 84

1.0 General Considerations

All reactions were performed under inert conditions using standard Schlenk techniques or in an *MBraun Unilab* glovebox (< 0.1 ppm H_2O / O_2).

Unless otherwise stated, solvents were degassed with nitrogen, dried over activated aluminium oxide (Solvent Purification System: *Inert PureSolv MD5 SPS*) and stored over 3 Å molecular sieves in ampules equipped with J. Young's valves. Chlorobenzene, 1,2-difluorobenzene and 1,2-dichlorobenzene were dried over calcium hydride, distilled and stored over 3 Å molecular sieves. Deuterated solvents ($CDCl_3$, CD_2Cl_2, C_6D_6 and C_6D_5Br (99.6% D, Sigma Aldrich)) were dried and stored over 3 Å molecular sieves. All chemicals were, unless stated otherwise, purchased from commercial sources and used as received. $BH_3{\cdot}SMe_2$ and BCl_3 in DCM (1M) were transferred to ampules fitted with J. Young's valves prior to use.

Column chromatography was performed on a *Teledyne Isco CombiFlash® 100* instrument using *Advion-Interchim* columns (spherical silica, 25 µm; or C18 silanised silica, 30 µm, for RP chromatography).

NMR spectra (^{1}H, $^{1}H\{^{11}B\}$, ^{2}H, ^{11}B, $^{11}B\{^{1}H\}H$, $^{13}C\{^{1}H\}$ and ^{19}F) were recorded on *Bruker Avance III 400 MHz*, *Bruker Avance III 500 MHz or Bruker PRO 500 MHz* spectrometers. Chemical shifts (δ) are quoted in parts per million (ppm), coupling constants (J) are given in hertz (Hz) to the nearest 0.5 Hz, and as positive vales regardless of their real individual signs. ^{1}H and ^{13}C shifts are referenced to the appropriate residual solvent peak while ^{11}B and ^{19}F shifts are referenced relative to external $BF_3{\cdot}Et_2O$ and C_6F_6, respectively. Abbreviations used are s (singlet), d (doublet), t (triplet), q (quartet), p (pentet), dd (doublet of doublets), dt (doublet of triplets), m (multiplet), br (broad). ^{13}C resonances of carbon atoms directly bonded to boron atoms were not always observed due to the quadrupolar relaxation effects. The observation of very broad signals at *ca*. 0 ppm in ^{11}B NMR spectra owes to the use of borosilicate glass NMR tubes and boron-containing parts in the NMR cavity. Unless otherwise stated, all NMR spectra were recorded at 20 °C.

Mass spectrometry was performed by the *Scottish Instrumentation and Resource Centre for Advanced Mass Spectrometry* (SIRCAMS) at the University of Edinburgh using electron impact (EI) or electrospray ionisation (ESI) techniques.

2.0 Synthesis and Activation of Pyrazaboles

2.1 Synthesis of Pyrazabole (1)

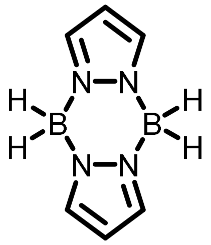

Neat $BH_3 \cdot SMe_2$ (4.0 ml, 42.2 mmol) was slowly added to a solution of pyrazole (2.87 g, 42.2 mmol, 1 equiv.) in DCM (20 ml) at 0 °C. After the initial gas evolution stopped the reaction was heated for 18 hrs at 35 °C in an unpressurized system. All volatiles were removed *in vacuo* and the remaining white solid was sublimed (120 °C, $4x10^{-2}$ mbar) to yield the product as a white powder in 50% yield (1.67 g, 10.45 mmol). Analytical data are in accordance with literature values.[1]

^{1}H NMR (500 MHz, $CDCl_3$) δ 7.61 (d, $^{3}J_{HH}$ = 2 Hz, 4H), 6.31 (t, $^{3}J_{HH}$ = 2 Hz, 2H), 3.61 (1:1:1:1 q, $^{1}J_{BH}$ = 101 Hz, 4H); **^{11}B NMR** (161 MHz, $CDCl_3$) δ -8.5 (t, $^{1}J_{BH}$ = 101 Hz); **^{13}C{^{1}H} NMR** (126 MHz, $CDCl_3$) δ 135.08, 105.54 ppm.

2.2 Synthesis of *bis*-NTf_2 Pyrazabole (2)

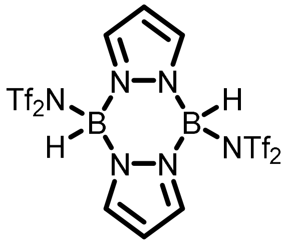

A solution of $HNTf_2$ (5.62 g, 20 mmol, 2 equiv.) in DCM (30 ml) was added dropwise to a solution of pyrazabole (1.60 g, 10 mmol) in DCM (1 ml) leading to gas evolution and precipitation of a white solid. The suspension was filtered after stirring at room temperature for 16 hours. The obtained white solid was washed with DCM (3 x 5 ml) and dried *in vacuo*. The product was obtained as a white powder in 74% yield (5.294 g, 7.37 mmol). Crystals suitable for X-Ray diffraction analysis were obtained from a less concentrated reaction by dissolving $HNTf_2$ (0.011 g, 0.039 mmol, 1.25 eq) and pyrazabole (0.005 g, 0.031 mmol) in DCM (0.5 ml) and letting it stand for 1 hour at room temperature. Very low solubility of **2** in polar, weakly-coordinating solvents resulted in poor NMR data (see below), 1,2-difluorobenzene gave the best (but still limited) solubility for solvents that did not react with **2**, thus ^{11}B NMR data is reported in this solvent. Better NMR data could be obtained using MeCN-d_3 but this coordinating solvent formed **2**-$(MeCN)_2$ (see next entry).

^{11}B NMR (160 MHz, $C_6H_4F_2$) δ -3.4 (d, $^{1}J_{BH}$ = 86 Hz, B*H*); **^{11}B{^{1}H} NMR** (160 MHz, $C_6H_4F_2$) δ -3.6 (s, B*H*) ppm.

HRMS (ESI$^+$) *m/z* calcd for $C_{10}H_7B_2F_{12}N_6O_8S_4^+$: 716.9221

5 [M-(H⁻)]⁺, found 716.9197.

2.3 Synthesis of *bis*-MeCN Pyrazabole 2-(MeCN)$_2$

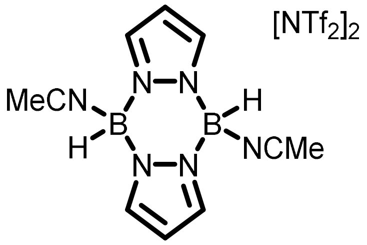

Compound **2** [Tf$_2$N(H)B(pyrazole)]$_2$ (0.011 g, 0.015 mmol) was dissolved in MeCN-d$_3$ (0.5 ml). Crystals suitable for X-Ray diffraction were obtained after removing all volatiles *in vacuo*, dissolving the solid in PhCl at 100 °C and cooling by 20 °C per day till 40 °C was reached. The sample was then kept at 40 °C for 3 days.

^{1}H NMR (500 MHz, CD$_3$CN) δ 8.33 (d, $^3J_{HH}$ = 3 Hz, 4H, NC(*H*)=C), 6.89 (t, $^3J_{HH}$ = 3 Hz, 2H, C=C(*H*)C), 4.50-4.19 (br m 2H, B*H*); **^{1}H{^{11}B} NMR** (500 MHz, CD$_3$CN) δ 8.33 (d, $^3J_{HH}$ = 3 Hz, 4H, NC(*H*)=C), 6.89 (t, $^3J_{HH}$ = 3 Hz, 2H, C=C(*H*)C), 4.33 (s, 2H, B*H*); ^{11}B NMR (160 MHz, CD$_3$CN) δ -7.9 (d, $^1J_{BH}$ = 136 Hz, *B*H); **^{11}B{^{1}H} NMR** (160 MHz, CD$_3$CN) δ -7.9 (s, *B*H); **^{19}F NMR** (471 MHz, CD$_3$CN) δ -80.14– -80.17 (m, CF$_3$); **^{13}C{^{1}H} NMR** (126 MHz, CD$_3$CN) δ 143.9 (s, N*C*(H)=C) 121.0 (q, $^1J_{CF}$ = 321 Hz, CF$_3$), 111.3 (s, C=*C*(H)C) ppm.

The compound could not be observed by using (EI) mass spectrometry (only compound **2** was detected).

2.4 Formation of *mono*-NTf$_2$ Pyrazabole (3) *in situ*

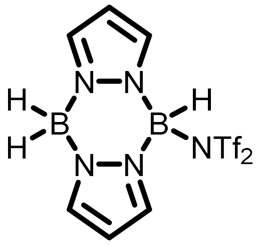

Pyrazabole (**1**) (0.005 g, 0.03 mmol) and **2** [Tf$_2$N(H)B(pyrazole)]$_2$ (0.023 g, 0.032 mmol, 1 equiv.) were suspended in CD$_2$Cl$_2$ (0.55 ml) and NMR data was recorded which showed v. broad resonances in the NMR spectra and no resonances for **1**. For spectra see below (Figure S14 to Figure S15). Note some compound **2** remains insoluble.

2.5 NMR Spectra of Synthesised Pyrazaboles

<u>NMR Spectra of Pyrazabole (**1**):</u>

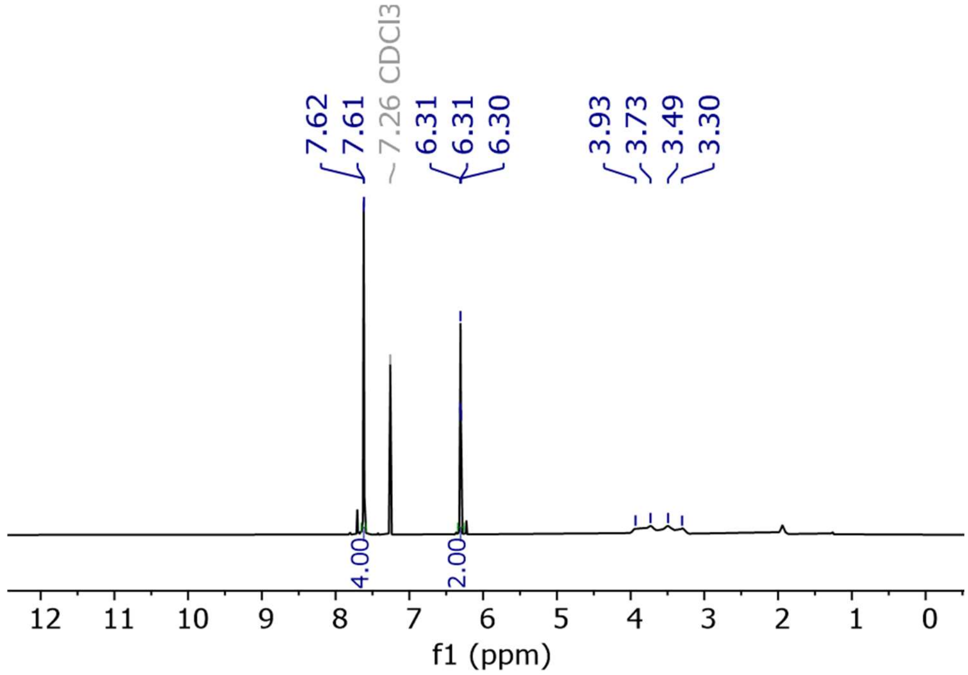

Figure S1: ^{1}H NMR spectrum of pyrazabole (**1**) in $CDCl_3$.

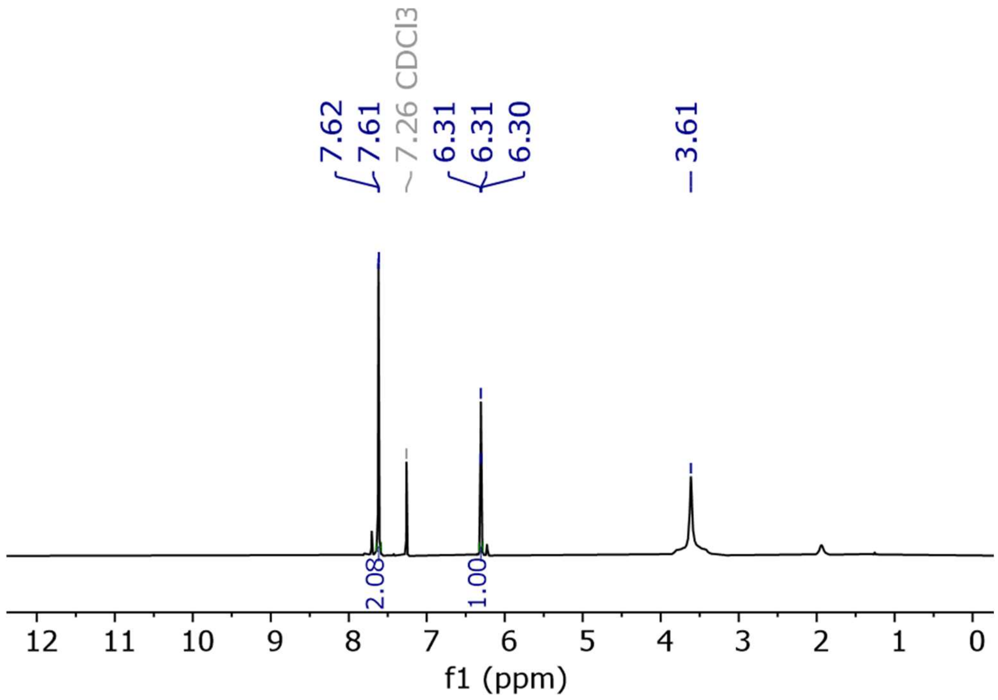

Figure S2: $^{1}H\{^{11}B\}$ NMR spectrum of pyrazabole (**1**) in $CDCl_3$.

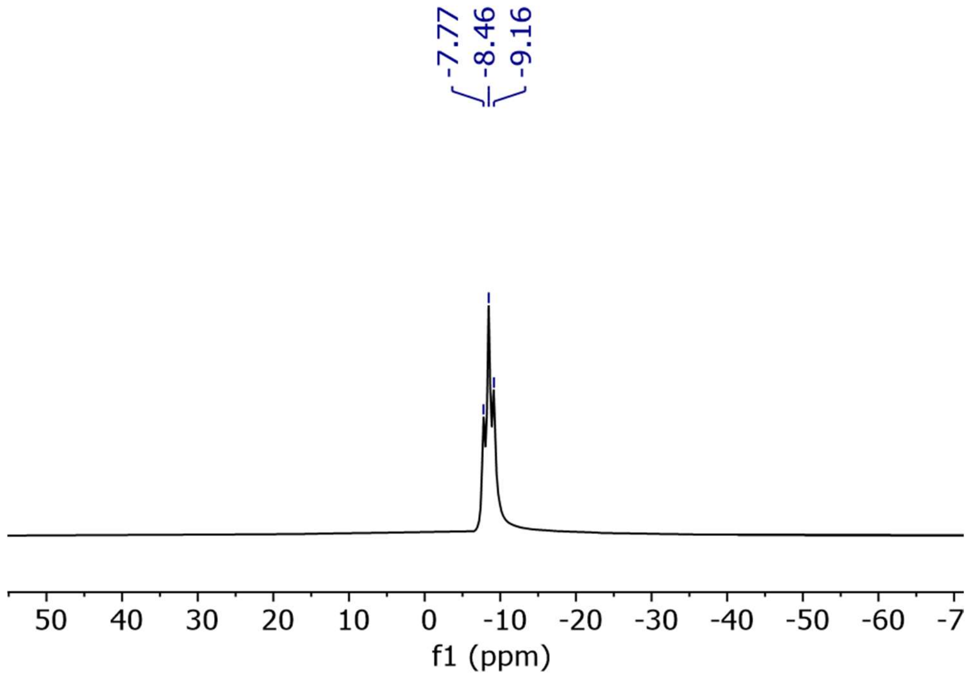

Figure S3: ^{11}B NMR spectrum of pyrazabole (**1**) in $CDCl_3$.

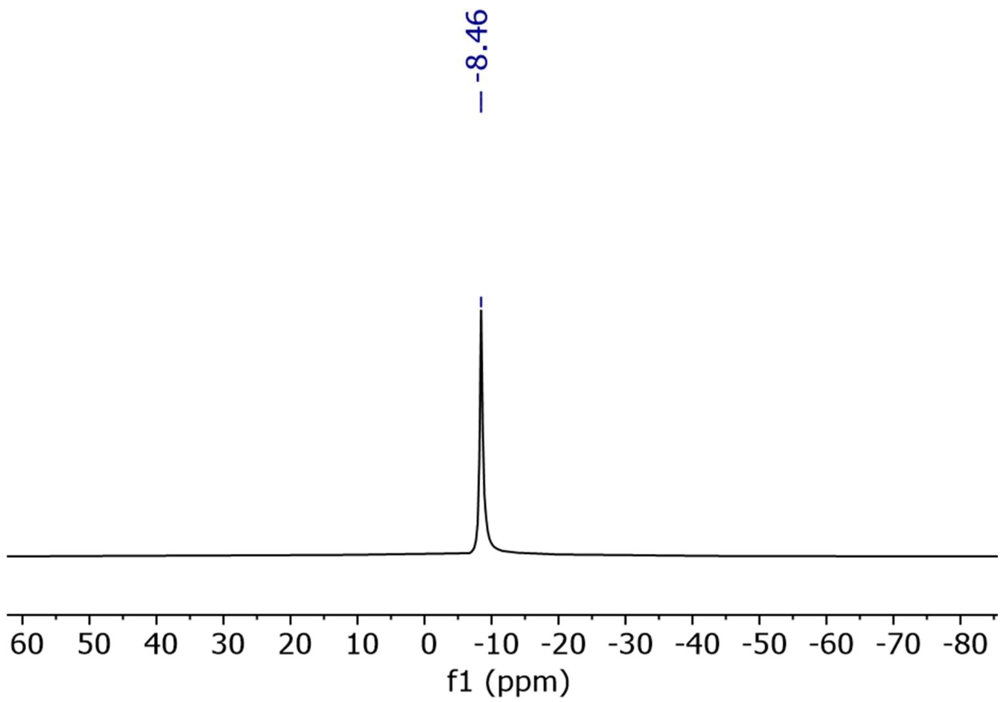

Figure S4: $^{11}B\{^1H\}$ NMR spectrum of pyrazabole (**1**) in $CDCl_3$.

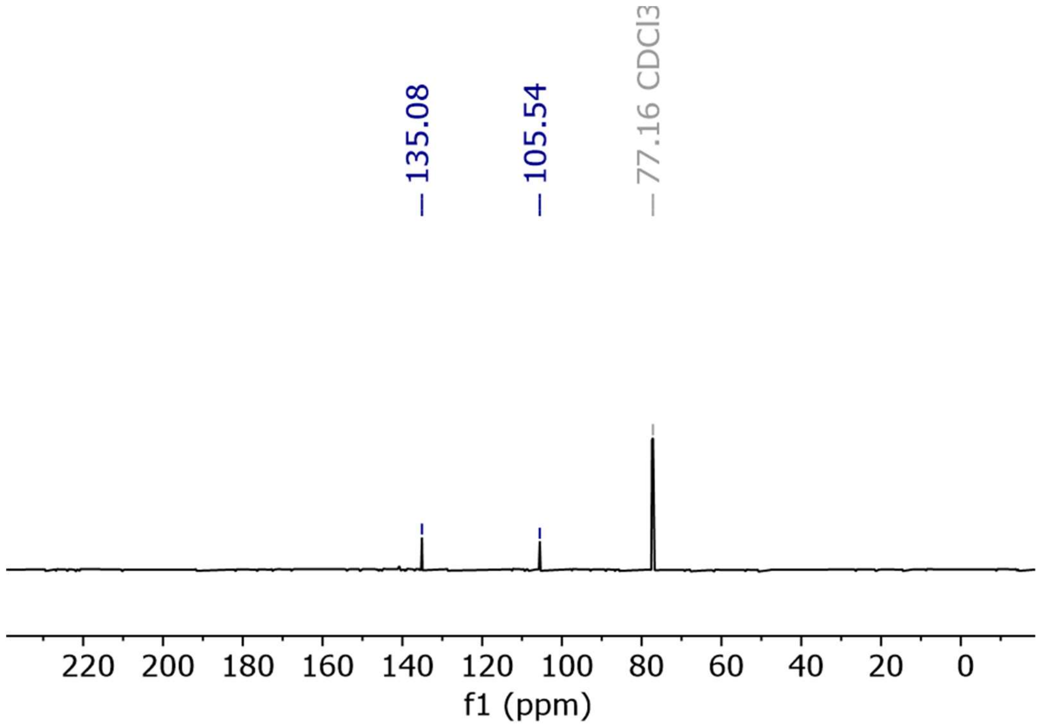

Figure S5: $^{13}C\{^{1}H\}$ NMR spectrum of pyrazabole (**1**) in $CDCl_3$.

NMR Spectra of *bis*-NTf2 Pyrazabole (**2**):

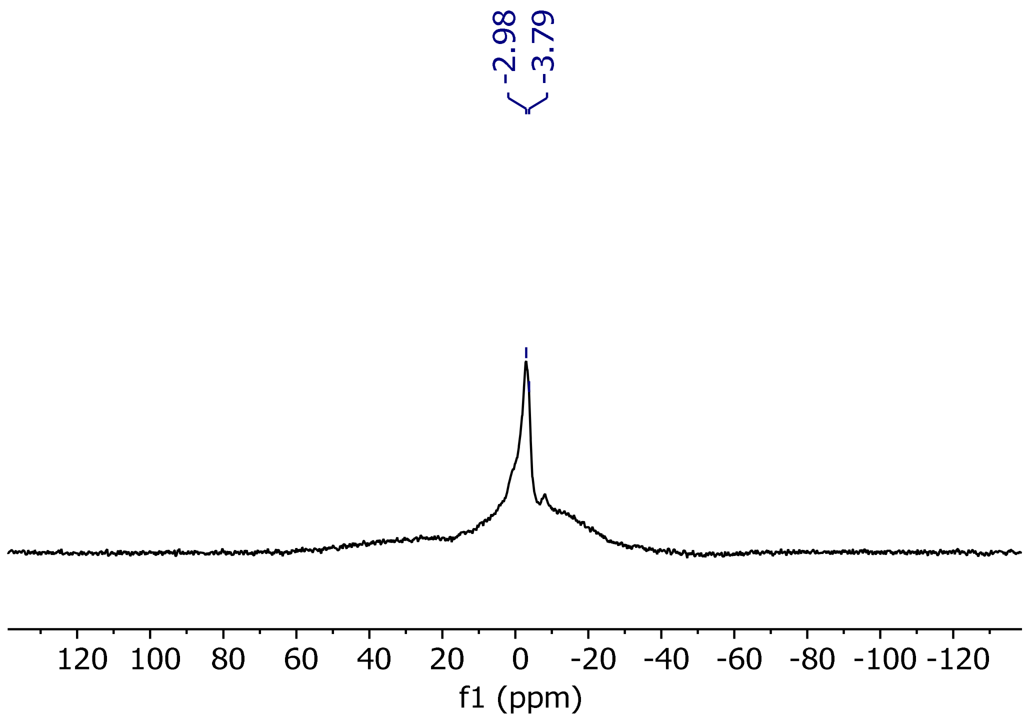

Figure S6: ^{11}B NMR spectrum of *bis*-NTf2 pyrazabole (**2**) in $C_6H_4F_2$.

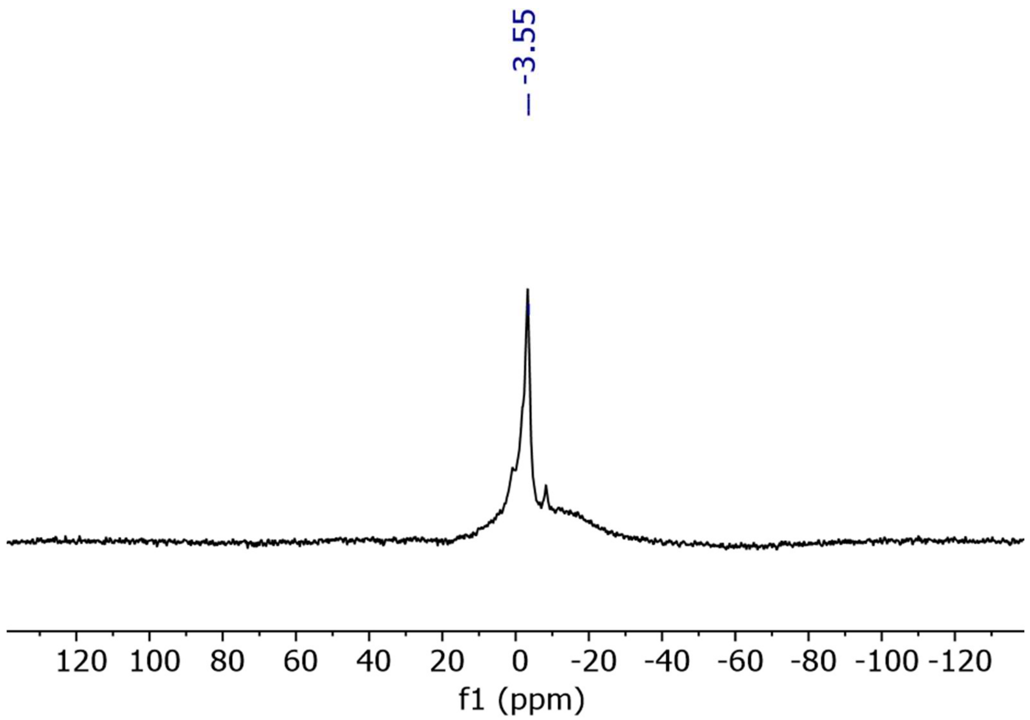

Figure S7: $^{11}B\{^1H\}$ NMR spectrum of *bis*-NTf2 pyrazabole (**2**) in $C_6H_4F_2$.

NMR Spectra of *bis*-MeCN Pyrazabole **2**-(MeCN)2

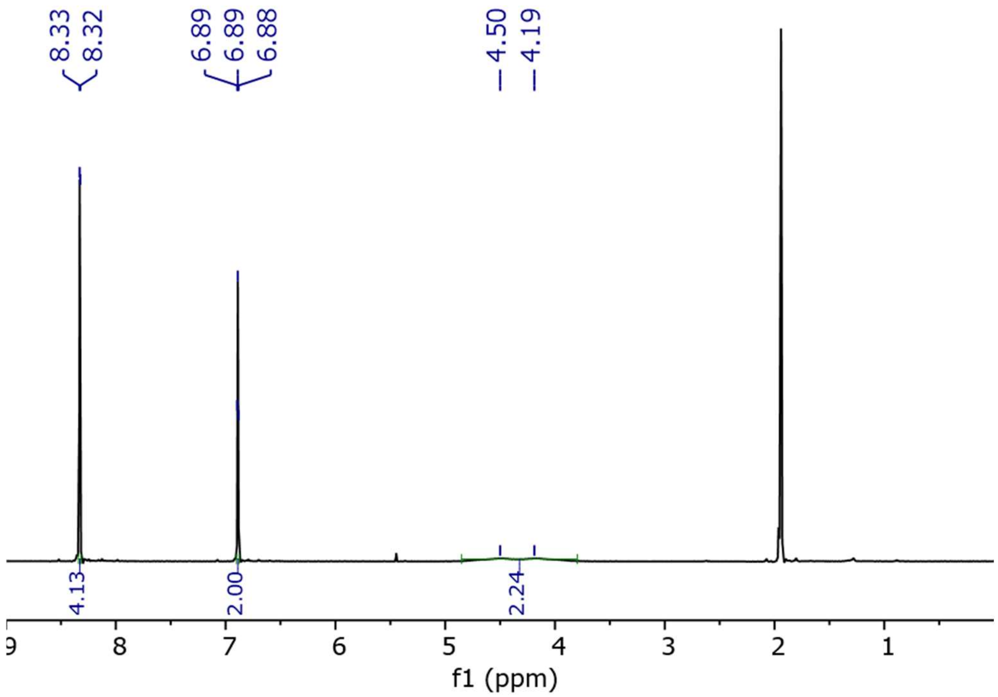

Figure S8: ^{1}H NMR spectrum of *bis*-MeCN pyrazabole **2**-(MeCN)2 in CD_3CN.

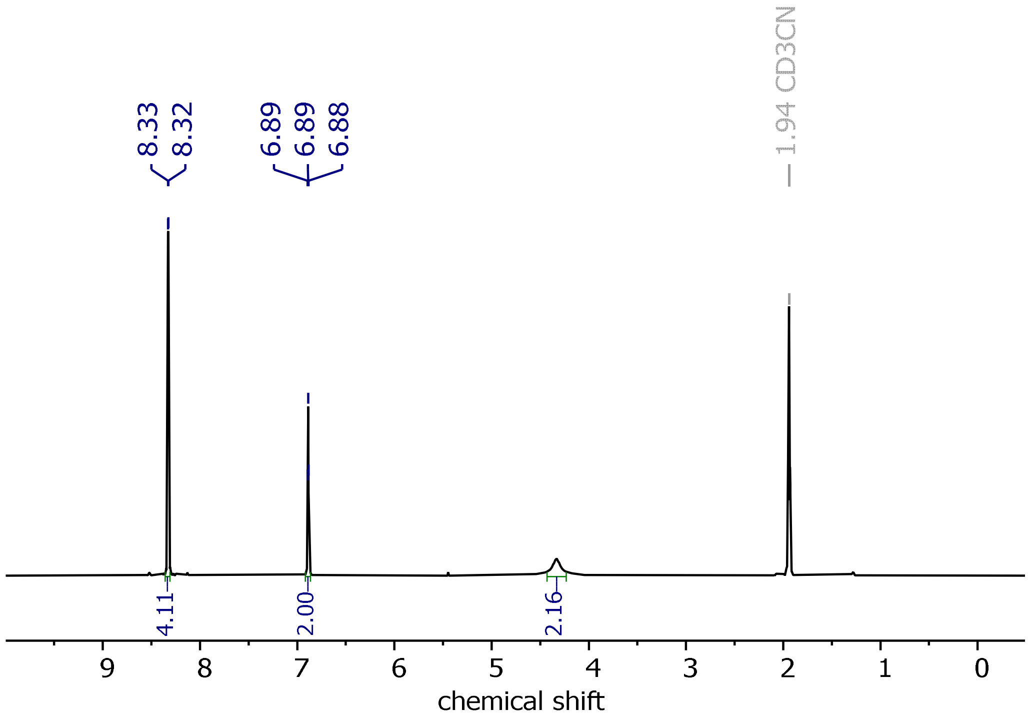

Figure S9: $^{1}H\{^{11}B\}$ NMR spectrum of *bis*-MeCN pyrazabole **2**-(MeCN)2 in CD_3CN.

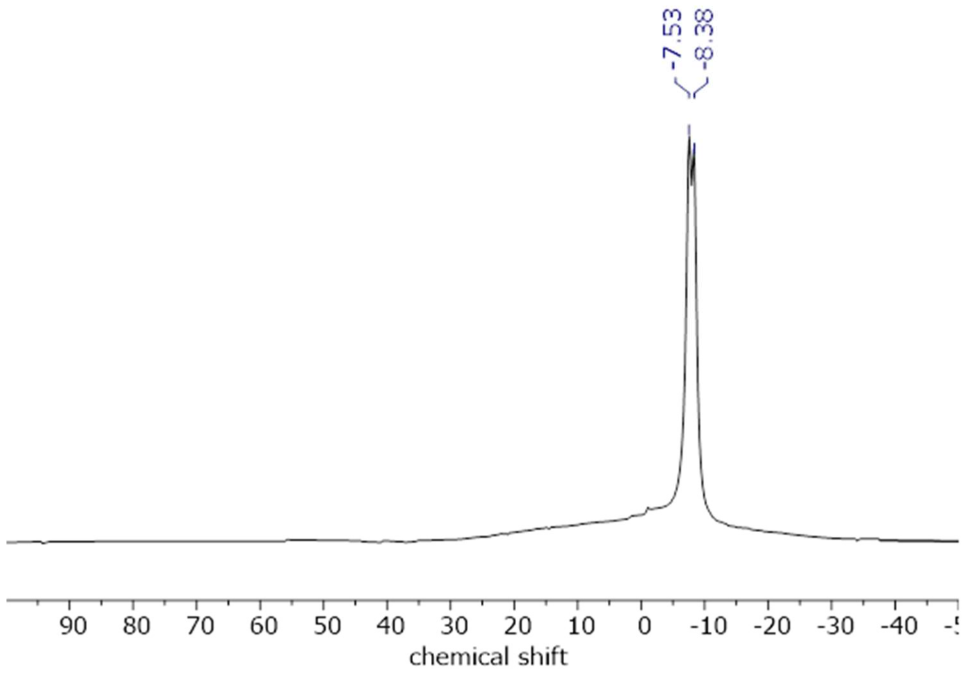

Figure S10: ^{11}B NMR spectrum of *bis*-MeCN pyrazabole **2**-$(MeCN)_2$ in CD_3CN.

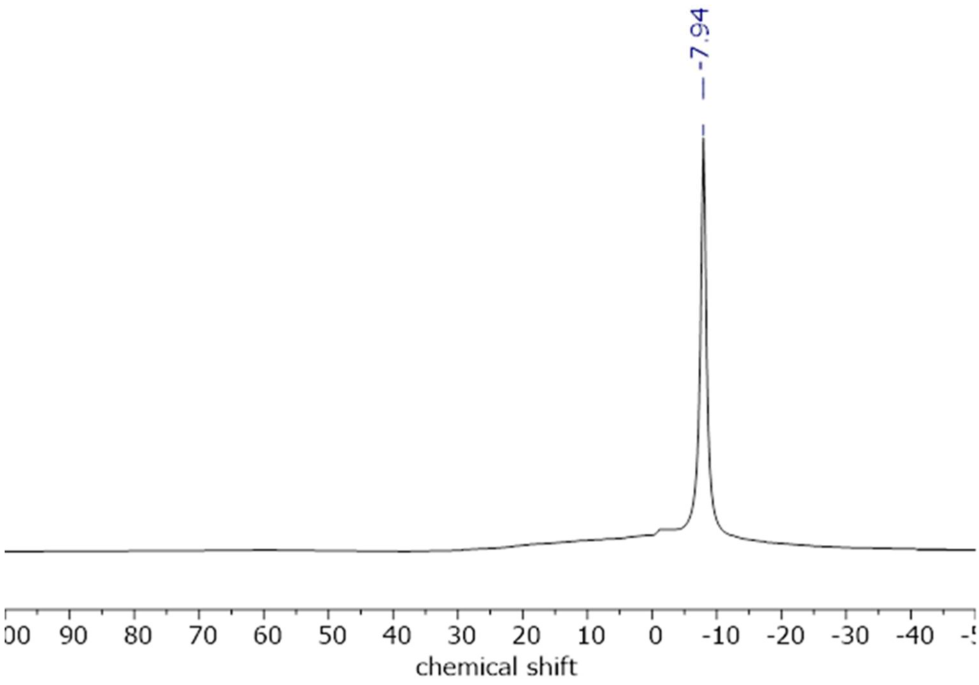

Figure S11: $^{11}B\{^1H\}$ NMR spectrum of *bis*-MeCN pyrazabole **2**-$(MeCN)_2$ in CD_3CN.

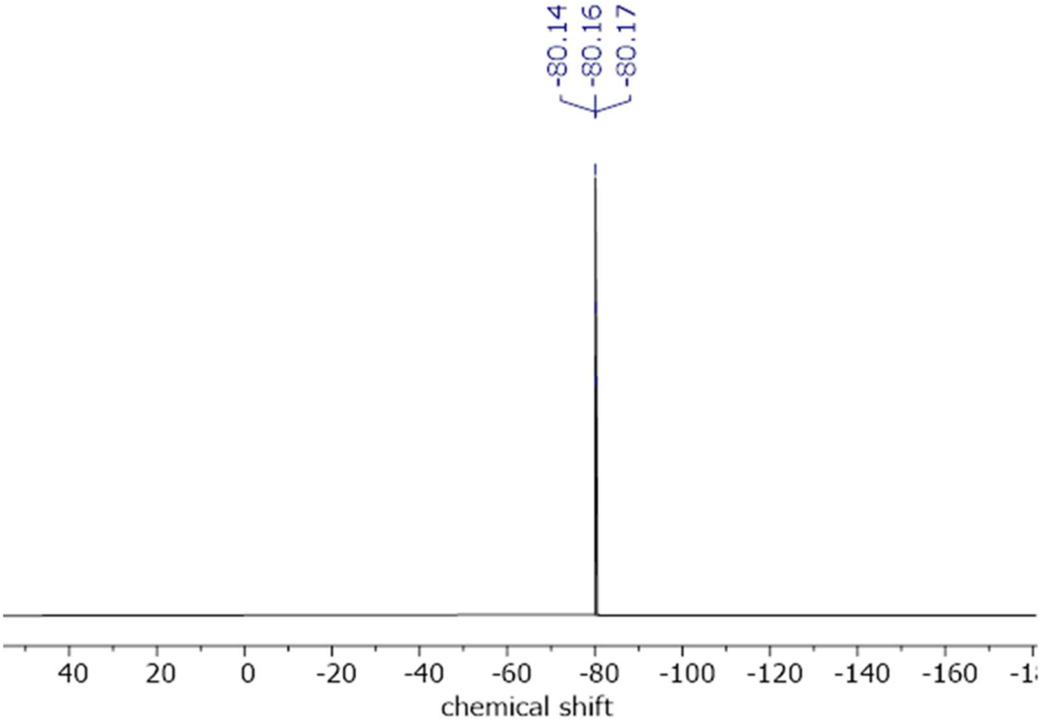

Figure S12: ^{19}F NMR spectrum of *bis*-MeCN pyrazabole **2**-(MeCN)$_2$ in CD_3CN.

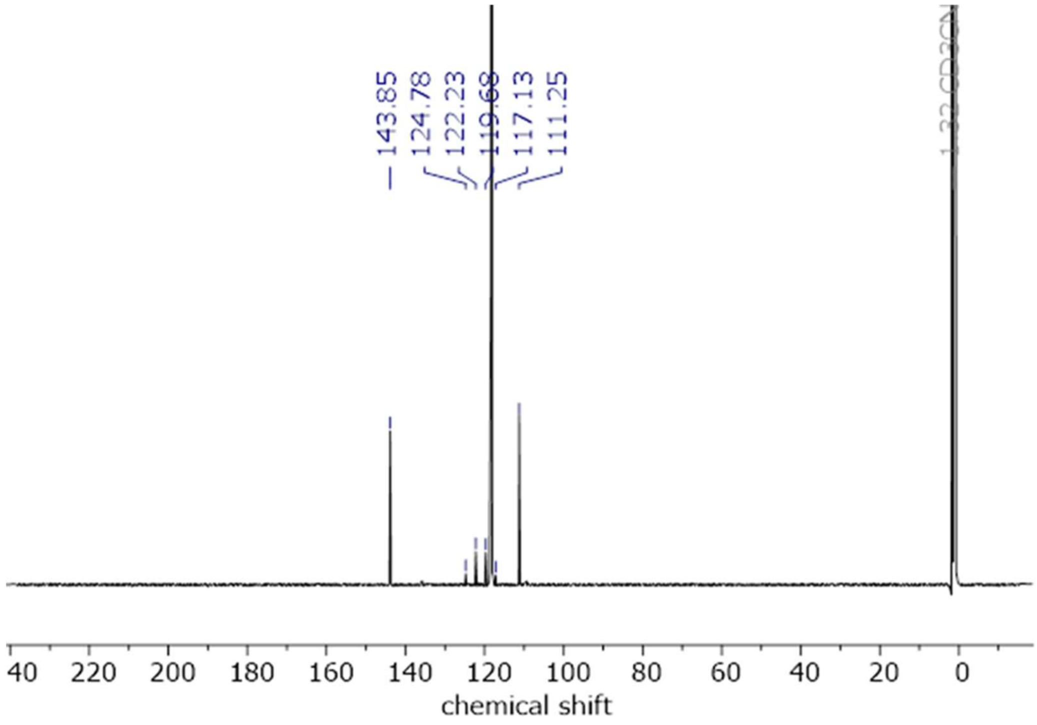

Figure S13: ^{13}C{^{1}H} NMR spectrum of *bis*-MeCN pyrazabole **2**-(MeCN)$_2$ in CD_3CN.

NMR Spectra of *mono*-NTf2 Pyrazabole (**3**):

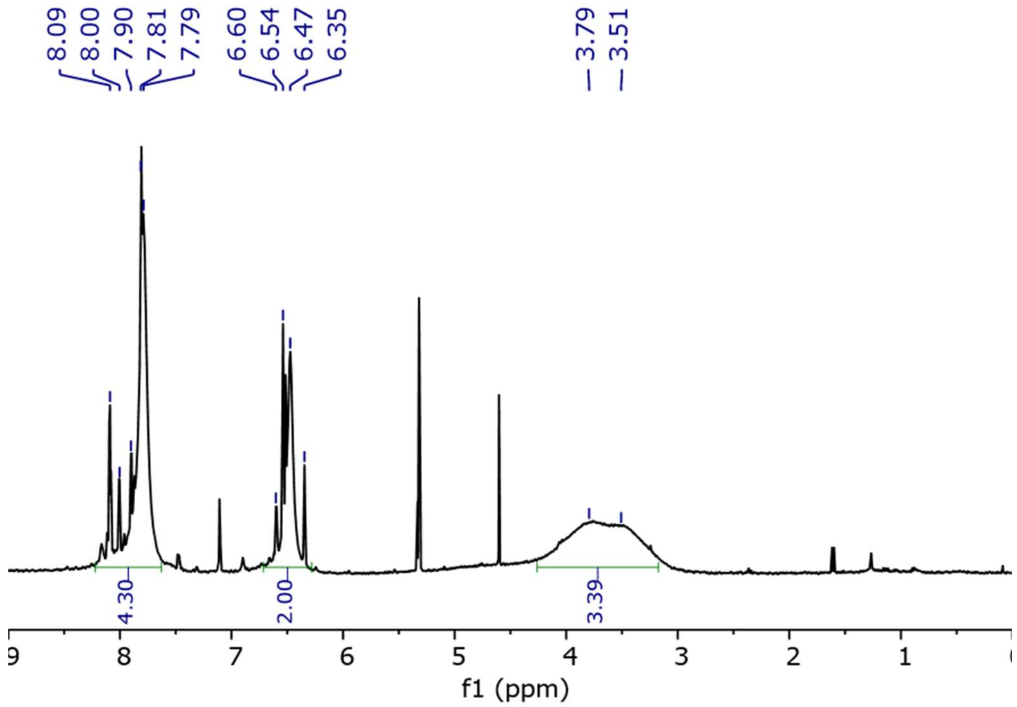

Figure S14: ^{1}H NMR spectrum of a 1:1 mixture of pyrazabole (**1**) and **2** formally resulting in **3**. Spectrum recorded in CD_2Cl_2. *Note: some insoluble **2** is present in this reaction*.

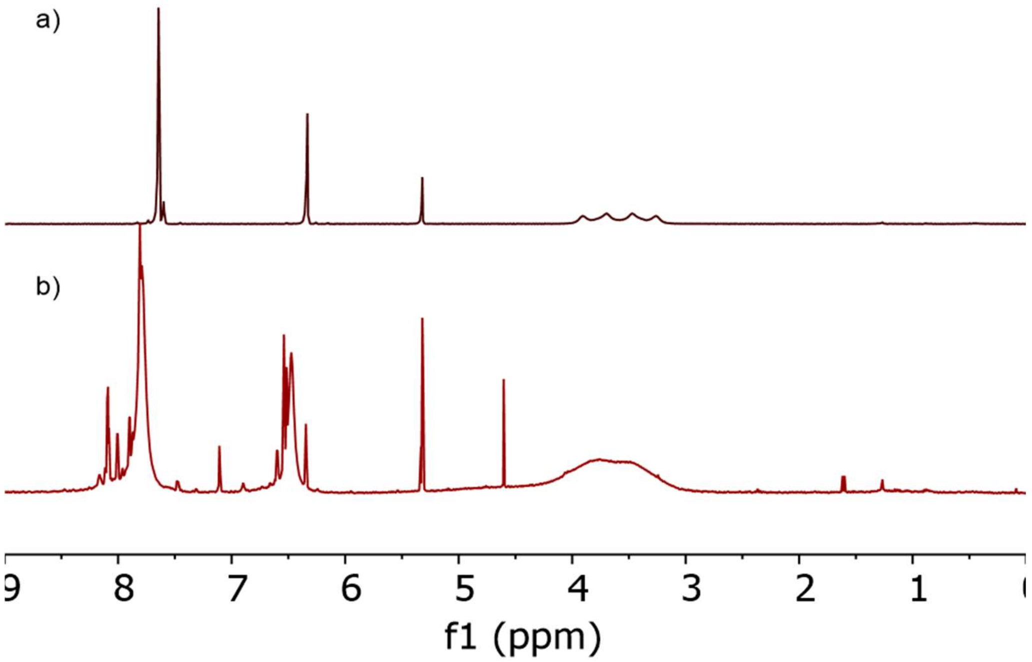

Figure S15: Stacked ^{1}H NMR spectra of pyrazabole (**1**) (top, **a**) before addition of **2**, and after addition of 1 equiv. of **2** (bottom, b). Spectra recorded in CD_2Cl_2. *Note: some insoluble **2** is present in this reaction.*

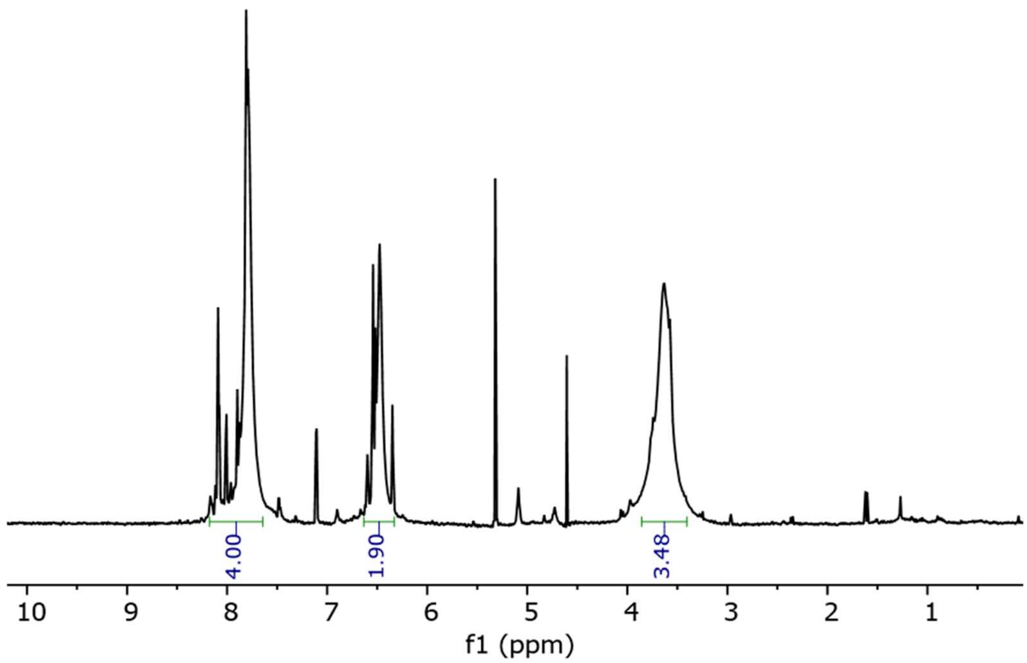

Figure S16: $^{1}H\{^{11}B\}$ NMR spectrum of a 1:1 mixture of pyrazabole (**1**) and **2** formally resulting in **3**. Spectrum recorded in CD_2Cl_2. *Note: some insoluble **2** is present in this reaction.*

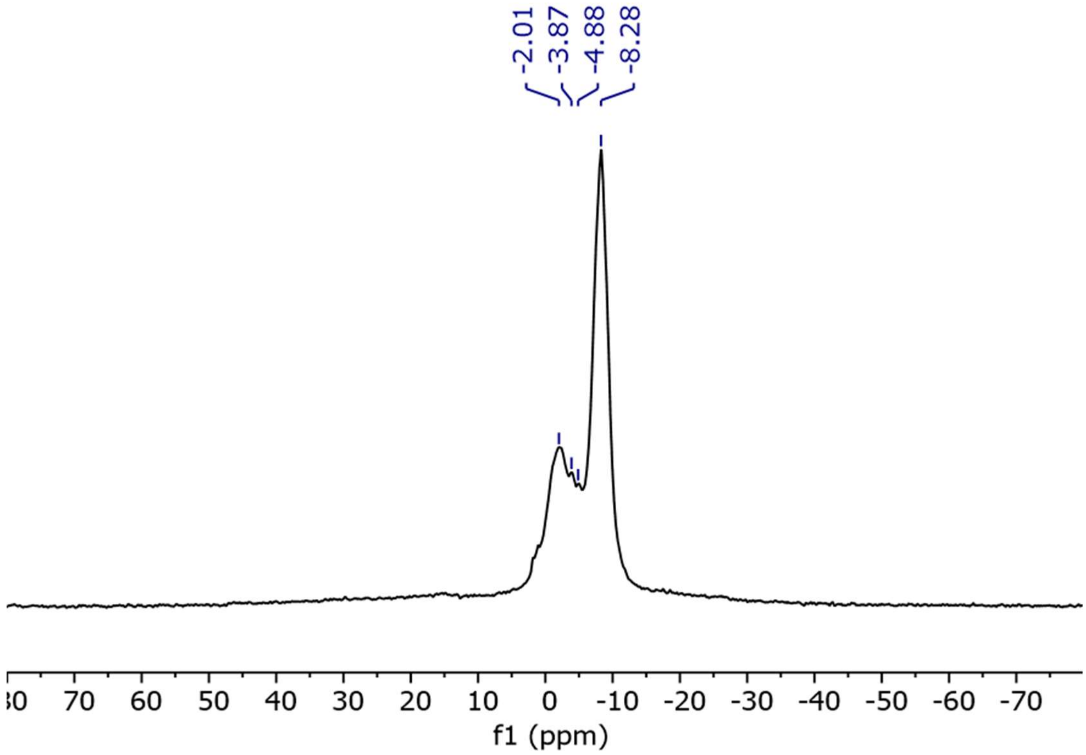

Figure S17: ^{11}B NMR spectrum of a 1:1 mixture of pyrazabole (**1**) and **2** formally resulting in **3**. Spectrum recorded in CD_2Cl_2. *Note: some insoluble **2** is present in this reaction. The resonance at -8.3 ppm corresponds to **1**.*

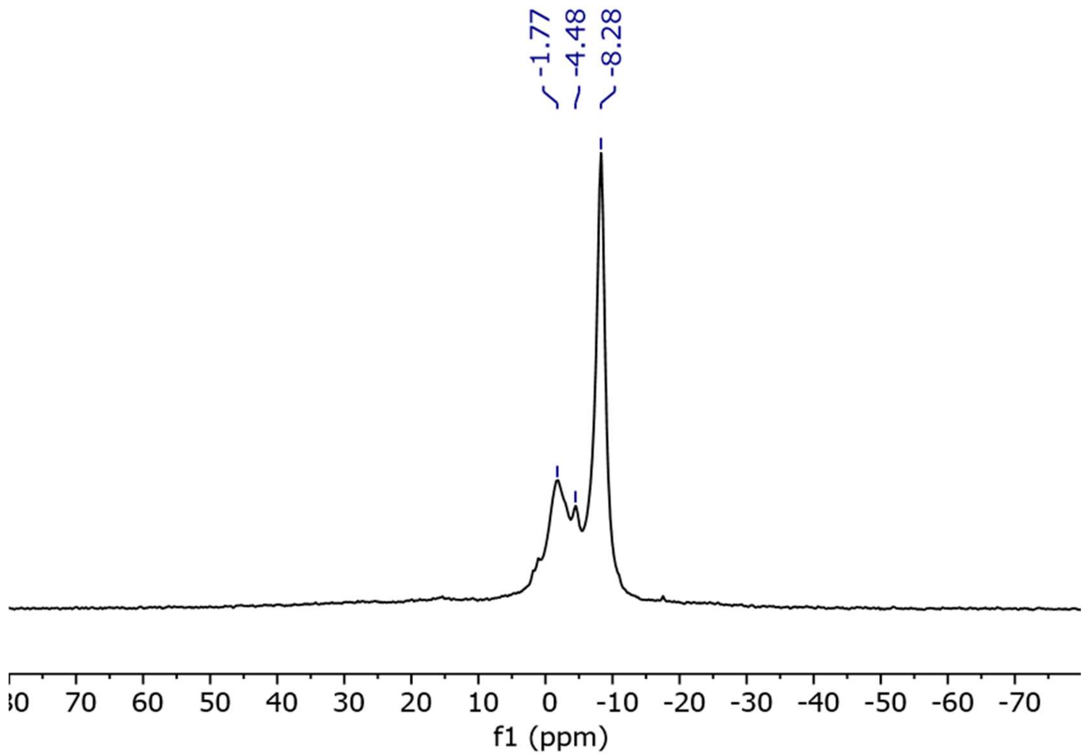

Figure S18: $^{11}B\{^{1}H\}$ NMR spectrum of a 1:1 mixture of pyrazabole (**1**) and **2** formally resulting in **3**. Spectrum recorded in CD_2Cl_2. *Note: some insoluble **2** is present in this reaction. The resonance at -8.3 ppm corresponds to **1**.*

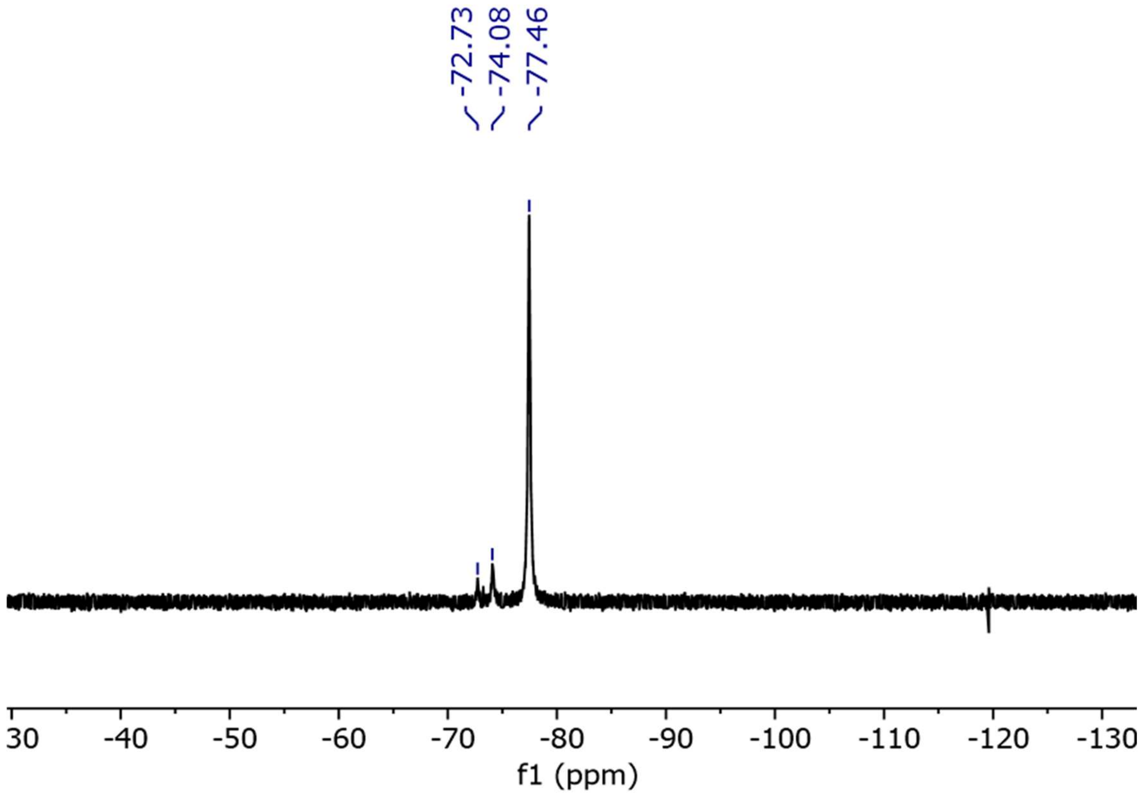

Figure S19: ^{19}F NMR spectrum of a 1:1 mixture of pyrazabole (**1**) and **2** resulting formally in **3**. Spectrum recorded in CD_2Cl_2. *Note: some insoluble **2** is present in this reaction.*

3.0 Directed C7-Borylation of N-H Indoles

3.1 General Procedure 1

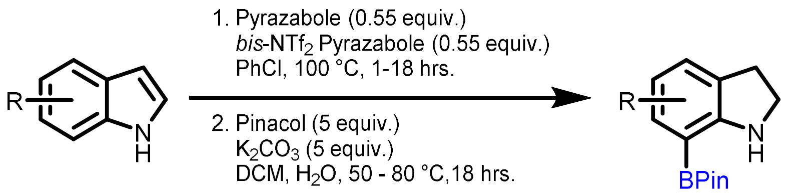

Pyrazabole **1** (0.026 g, 0.165 mmol, 0.55 equiv.), *bis*-NTf_2 Pyrazabole **2** (0.118 g, 0.165 mmol, 0.55 equiv.) and the corresponding indole (1 equiv., 0.3 mmol) were suspended in chlorobenzene (2 mL) and heated to 100 °C in a sealed ampule for 1-18 hours. Upon cooling, pinacol (0.177 g, 1.5 mmol, 5 equiv.), K_2CO_3 (0.207 g, 1.5 mmol, 5 equiv.), DCM (4 mL) and water (4 mL) were added sequentially, and the ampule was sealed and heated to either 50 °C or 80 °C for 18 hours, with rapid stirring to mix the resulting biphasic mixture. Upon returning to room temperature, the 7-BPin indoline product was extracted with pentane (4 x 10 mL). The combined organic phases were washed with water (4 x 10 mL), dried over $MgSO_4$, and filtered. Concentration *in vacuo* yielded the desired product. *Further purification was enabled by reverse phase chromatography (C18, MeOH), where required.*

3.2 Substrate Scope and Characterisation of 7-BPin Indolines (4a-k)

7-BPin Indoline (4a)

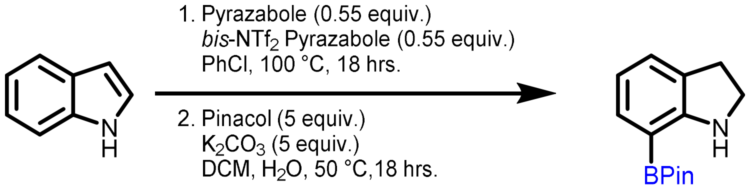

Compound **4a** was prepared following **General Procedure 1** with indole (0.035 g). Reaction was heated to 100 °C for 18 hours to effect C7-borylation, then to 50 °C for 18 hours to convert to the BPin product. The product was isolated as a white solid (0.057 g, 0.233 mmol, 78 %) with no further purification necessary.

^{1}H NMR (500 MHz, $CDCl_3$) δ 7.38 (d, $^3J_{HH}$ = 7 Hz, 1H, ArC*H*), 7.15 (d, $^3J_{HH}$ = 7 Hz, 1H, ArC*H*), 6.61 (t, $^3J_{HH}$ = 7 Hz, 1H, ArC*H*), 5.02 (br, 1H, N*H*), 3.60 (t, $^3J_{HH}$ = 8.5 Hz, 2H, H_2C-CH_2), 3.00 (t, $^3J_{HH}$ = 8.5 Hz, 2H, H_2C-CH_2), 1.33 (s, 12H, O-C(CH_3)$_2$); **^{11}B NMR** (160 MHz, $CDCl_3$) δ 31.0 (s, Ar*B*Pin); **^{13}C{^{1}H}** NMR (126 MHz, $CDCl_3$) δ 158.74, 133.67, 128.52, 127.65, 117.03, 106.23 (br, *C*-B, only detectable *via* ^{1}H ^{13}C HMBC), 83.53, 47.09, 29.30, 25.10 ppm.

HRMS (ESI$^+$) *m/z* calcd for $C_{14}H_{21}BNO_2^+$: 246.1660 [M+H]$^+$, found 246.1659.

5-Methyl 7-BPin Indoline (4b)

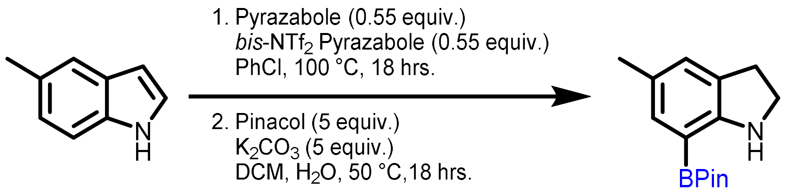

Compound **4b** was prepared following **General Procedure 1** with 5-methylindole (0.039 g). Reaction was heated to 100 °C for 18 hours to effect C7-borylation, then to 50 °C for 18 hours to convert to the BPin product. The product was isolated as a yellow solid (0.059 g, 0.228 mmol, 76 %) with no further purification necessary.

^{1}H NMR (500 MHz, $CDCl_3$) δ 7.19 (s, 1H, ArC*H*), 7.00 (s, 1H, ArC*H*), 4.87 (br, 1H, N*H*), 3.57 (t, $^3J_{HH}$ = 8.5 Hz, 2H, H_2C-CH_2), 2.96 (t, $^3J_{HH}$ = 8.5 Hz, H_2C-CH_2), 2.22 (s, 3H, ArC-CH_3), 1.33 (s, 12H, O-C(CH_3)$_2$); **^{11}B NMR** (160 MHz, $CDCl_3$) δ 31.0 (s, Ar*B*Pin); **^{13}C{^{1}H} NMR** (126 MHz, $CDCl_3$) δ 156.63, 133.33, 129.04, 128.88, 126.27, 106.45 (br, *C*-B, only detectable *via* ^{1}H ^{13}C HMBC), 83.52, 47.40, 29.39, 25.09, 20.69 ppm.

HRMS (ESI$^+$) *m/z* calcd for $C_{15}H_{23}BNO_2^+$: 260.1816 [M+H]$^+$, found 260.1825.

3-Methyl 7-BPin Indoline (4c)

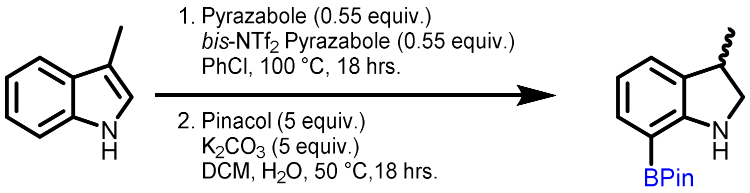

Compound **4c** was prepared following **General Procedure 1** with 3-methylindole (0.039 g). Reaction was heated to 100 °C for 18 hours to effect C7-borylation, then to 50 °C for 18 hours to convert to the BPin product. The product was isolated as a colourless oil (0.068 g, 0.262 mmol, 87 %) with no further purification necessary.

^{1}H NMR (500 MHz, $CDCl_3$) δ 7.40 (dt, $^4J_{HH}$ = 1 Hz, $^3J_{HH}$ = 7.5 Hz, 1H, ArC*H*), 7.13 (dt, $^4J_{HH}$ = 1 Hz, $^3J_{HH}$ = 7.5 Hz, 1H, ArC*H*), 6.65 (t, $^3J_{HH}$ = 7.5 Hz, 1H, ArC*H*), 4.93 (br, 1H, N*H*), 3.76 (dd, $^3J_{HH}$ = 8.5 Hz, 1H, (H_3C)CH-CH_2), 3.35 (m, 1H, (H_3C)C*H*-CH_2), 3.17 (dd, $^3J_{HH}$ = 8.5 Hz, 1H, (H_3C)CH-CH_2), 1.34 (s, 12H, O-C(CH_3)$_2$), 1.33 (d, $^3J_{HH}$ = 7 Hz, 3H, (H_3C)C(H)-CH_2); **^{11}B NMR** (160 MHz, $CDCl_3$) δ 30.9 (s, Ar*B*Pin); **^{13}C{^{1}H} NMR** (126 MHz, $CDCl_3$) δ 158.31, 133.76, 133.52, 126.42, 117.06, 106.46 (br, *C*-B, only detectable *via* ^{1}H ^{13}C HMBC), 83.49, 55.18, 36.06, 25.11, 25.04 (inequivalent pinacol Me resonances), 18.87 ppm.

HRMS (ESI$^+$) *m/z* calcd for $C_{15}H_{23}BNO_2^+$: 260.1816 [M+H]$^+$, found 260.1826.

2-Methyl 7-BPin Indoline (4d)

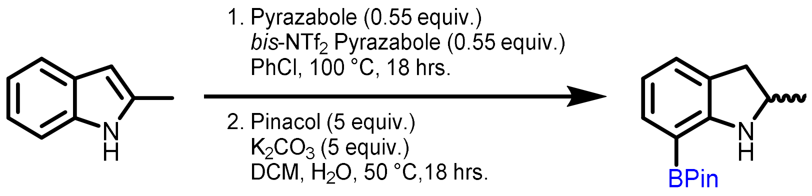

Compound **4d** was prepared following **General Procedure 1** with 2-methylindole (0.039 g). Reaction was stirred at room temperature for 1 hour (hydroboration step is slower for 2-substituted indoles), heated to 100 °C for 18 hours to effect C7-borylation, then to 50 °C for 18 hours to convert to the BPin product. The product was isolated as a yellow oil (0.067 g, 0.259 mmol, 86 %) with no further purification necessary.

^{1}H NMR (500 MHz, $CDCl_3$) δ 7.40 (d, $^3J_{HH}$ = 7.5 Hz, 1H, ArC*H*), 7.13 (d, $^3J_{HH}$ = 7.5 Hz, 1H, ArC*H*), 6.62 (dd, $^3J_{HH}$ = 7.5 Hz, 1H, ArC*H*), 5.05 (br, 1H, N*H*), 4.05 (m, 1H, H_2C-C*H*(CH_3)), 3.13 (dd, J_{HH} = 16 Hz, 8.5 Hz, 1H, H_2C-CH(CH_3)), 2.63 (dd, J_{HH} = 16 Hz, 8.5 Hz, 1H, H_2C-CH(CH_3)), 1.36 (s, 6H, O-C(CH_3)$_2$), 1.35 (s, 6H, O-C(CH_3)$_2$), 1.33 (d, $^3J_{HH}$ = 6 Hz, 3H, H_2C-CH(CH_3)); **^{11}B NMR** (160 MHz, $CDCl_3$) δ 31.0 (s, Ar*B*Pin); **^{13}C{^{1}H} NMR** (126 MHz, $CDCl_3$) δ 158.0, 133.68, 128.10, 127.67, 116.89, 105.96 (br, *C*-B, only detectable *via* ^{1}H ^{13}C HMBC), 83.44, 54.97, 37.32, 25.12, 25.0 (inequivalent pinacol Me resonances), 22.39 ppm.

HRMS (ESI$^+$) *m/z* calcd for $C_{15}H_{23}BNO_2^+$: 260.1816 [M+H]$^+$, found 260.1827.

6-Methyl 7-BPin Indoline (4e)

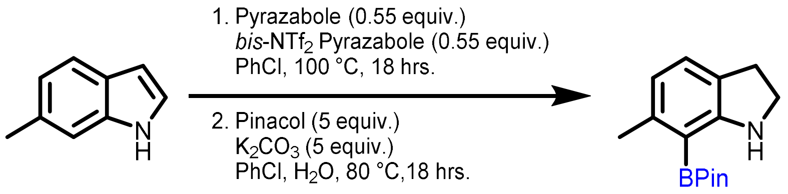

Compound **4e** was prepared following **General Procedure 1** with 6-methylindole (0.039 g). Reaction was heated to 100 °C for 18 hours to effect C7-borylation, then to 80 °C for 18 hours (PhCl instead of DCM) to convert to the BPin product. The product was isolated as a yellow oil (0.043 g, 0.166 mmol, 55 %) with no further purification necessary.

^{1}H NMR (500 MHz, $CDCl_3$) δ 7.00 (d, $^3J_{HH}$ = 7 Hz, ArC*H*) 6.43 (d, $^3J_{HH}$ = 7 Hz, ArC*H*) 5.34 (br, 1H, N*H*), 3.57 (t, $^3J_{HH}$ = 8 Hz, 2H, H_2C-CH_2), 2.95 (t, $^3J_{HH}$ = 8 Hz, 2H, H_2C-CH$_2$), 2.44 (s, 3H, Ar-CH_3), 1.33 (s, 12H, O-C(CH_3)$_2$); **^{11}B NMR** (160 MHz, $CDCl_3$) δ 31.1 (s, Ar*B*Pin); **^{13}C{^{1}H} NMR** (126 MHz, $CDCl_3$) δ 160.10, 144.46, 126.87, 125.93, 119.05, 106.30 (*C*-B), 82.95, 47.19, 29.05, 25.12, 22.77 ppm.

HRMS (ESI$^+$) *m/z* calcd for $C_{15}H_{23}BNO_2^+$: 260.1816 [M+H]$^+$, found 260.1820.

5-Chloro 7-BPin Indoline (4f)

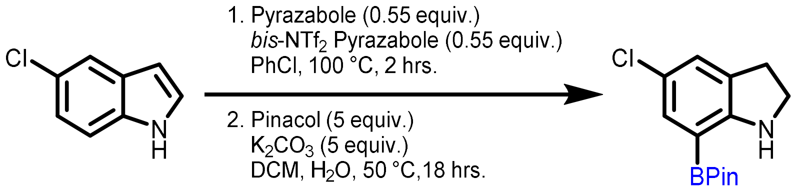

Compound **4f** was prepared following **General Procedure 1** with 5-chloroindole (0.045 g). Reaction was heated to 100 °C for 2 hours to effect C7-borylation, then to 50 °C for 18 hours to convert to the BPin product. The product was isolated as an orange oil (0.067 g, 0.240 mmol, 80 %) with no further purification necessary. *Note: the broad N-H resonance is not observed for this substrate in the ^{1}H NMR spectrum.*

^{1}H NMR (500 MHz, $CDCl_3$) δ 7.32 (d, $^{4}J_{HH}$ = 2 Hz, 1H, ArC*H*), 7.08 (d, $^{4}J_{HH}$ = 2 Hz, 1H, ArC*H*), 3.62 (t, $^{3}J_{HH}$ = 8 Hz, 2H, H_2C-CH_2), 2.99 (t, $^{3}J_{HH}$ = 8 Hz, 2H, H_2C-CH_2), 1.32 (s, 12H, O-C(CH_3)$_2$); **^{11}B NMR** (160 MHz, $CDCl_3$) δ 30.6 (s, Ar*B*Pin); **^{13}C{^{1}H} NMR** (126 MHz, $CDCl_3$) δ 157.22, 132.60, 130.79, 127.74, 121.68, 105.59 (*C*-B), 83.86, 47.34, 29.16, 25.07 ppm.

HRMS (ESI^{+}) *m/z* calcd for $C_{14}H20BNO_2Cl^{+}$ = 280.1270 [M+H]$^{+}$, found 280.1274.

4-Fluoro 7-BPin Indoline (4g)

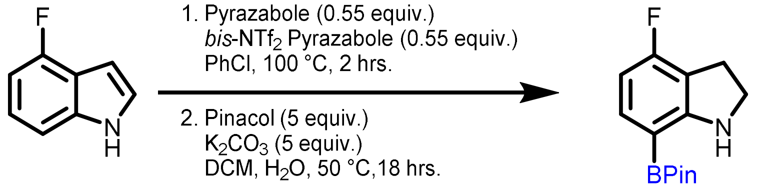

Compound **4g** was prepared following **General Procedure 1** with 4-fluoroindole (0.041 g). Reaction was heated to 100 °C for 2 hours to effect C7-borylation, then to 50 °C for 18 hours to convert to the BPin product. The product was isolated as a colourless oil (0.067 g, 0.255 mmol, 85 %) with no further purification necessary.

^{1}H NMR (500 MHz, $CDCl_3$) δ 7.35 (dd, $^{4}J_{HF}$ = 6.5 Hz, $^{3}J_{HH}$ = 8.5 Hz, 1H, ArC*H*), 6.31 (dd, $^{3}J_{HF}$ = 8.5 Hz, $^{3}J_{HH}$ = 8.5 Hz, 1H, ArC*H*), 5.12 (br, 1H, N*H*), 3.66 (t, $^{3}J_{HH}$ = 8.5 Hz, 2H, H_2C-CH_2), 3.04 (t, $^{3}J_{HH}$ = 8.5 Hz, 2H, H_2C-CH_2), 1.33 (s, 12H, O-C(CH_3)$_2$); **^{11}B NMR** (160 MHz, $CDCl_3$) δ 30.5 (s, Ar*B*Pin); ^{19}F NMR (471 MHz, $CDCl_3$) δ -114.59 (dd, $^{4}J_{HF}$ = 6.5 Hz, $^{3}J_{HF}$ = 8.5 Hz, ArC*F*); **^{13}C{^{1}H} NMR** (126 MHz, $CDCl_3$) δ 162.65 (d, $^{1}J_{CF}$ = 163 Hz), 161.62 (d, $^{3}J_{CF}$ = 74 Hz), 136.32 (d, $^{3}J_{CF}$ = 9 Hz), 113.17 (d, $^{2}J_{CF}$ = 20 Hz), 104.88 (d, $^{2}J_{CF}$ = 20 Hz), 102.31 (br, *C*-B, only detectable *via* ^{1}H ^{13}C HMBC), 83.55, 47.31, 25.54, 25.06 ppm.

HRMS (ESI^{+}) *m/z* calcd for $C_{14}H_{20}BFNO_2^{+}$ = 264.1566 [M+H]$^{+}$, found 264.1569.

4-Bromo 7-BPin Indoline (4h)

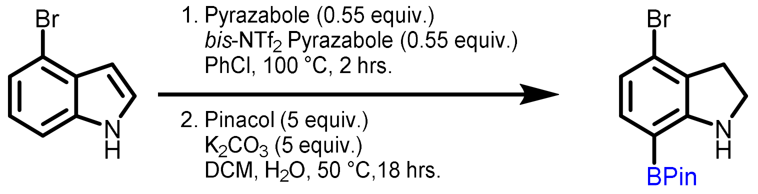

Compound **4h** was prepared following **General Procedure 1** with 4-bromoindole (0.059 g, 0.038 mL). Reaction was heated to 100 °C for 2 hours to effect C7-borylation, then to 50 °C for 18 hours to convert to the BPin product. The crude product was purified via reverse phase chromatography (4 g C18 column, celite dry load, 100% MeOH) to yield the product as a white solid (0.047 g, 0.145 mmol, 48%).

^{1}H NMR (500 MHz, $CDCl_3$) δ 7.20 (d, $^3J_{HH}$ = 8 Hz, 1H, ArC*H*), 6.71 (d, $^3J_{HH}$ = 8 Hz, 1H, ArC*H*), 5.16 (br, 1H, N*H*), 3.64 (t, $^3J_{HH}$ = 8.5 Hz, 2H, H_2C-CH_2), 3.02 (t, $^3J_{HH}$ = 8.5 Hz, 2H, H_2C-CH_2), 1.32 (s, 12H, O-C(CH_3)$_2$); **^{11}B NMR** (160 MHz, $CDCl_3$) δ 30.8 (s, Ar*B*Pin); **^{13}C{^{1}H} NMR** (126 MHz, $CDCl_3$) δ 159.46, 135.32, 128.87, 123.62, 119.86, 105.59 (*C*-B), 83.71, 46.09, 30.64, 25.07 ppm.

HRMS (ESI$^+$) *m/z* calcd for $C_{14}H_{20}BNO_2Br^+$: 324.0765 [M+H]$^+$, found 324.0765.

5-Methoxy 7-BPin Indoline (4i)

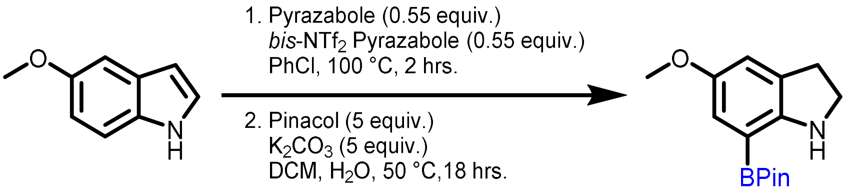

Compound **4i** was prepared following **General Procedure 1** with 5-(methoxy)indole (0.044 g). Reaction was heated to 100 °C for 2 hours to effect C7-borylation, then to 50 °C for 18 hours to convert to the BPin product. The product was isolated as a yellow oil (0.055 g, 0.200 mmol, 67 %) with no further purification necessary. *Note: the broad N-H resonance is not observed for this substrate in the ^{1}H NMR spectrum.*

^{1}H NMR (500 MHz, $CDCl_3$) δ 6.88 (m, 1H, ArC*H*), 6.84 (m, 1H, ArC*H*), 3.76 (s, 3**^{11}B NMR** (160 MHz, $CDCl_3$) δ 30.8 (s, Ar*B*Pin);**^{13}C{^{1}H} NMR** (126 MHz, $CDCl_3$) δ 153.32, 152.25, 130.82, 117.06, 115.46, 105.48 (br, *C*-B, only detectable *via* ^{1}H ^{13}C HMBC), 83.61, 56.29, 47.64, 29.64, 25.07 ppm.HRMS (ESI$^+$) *m/z* calcd for $C_{15}H_{23}BNO_3^+$ = 276.1766 [M+H]$^+$, found 276.1767.

5-Methylthio 7-BPin Indoline (4j)

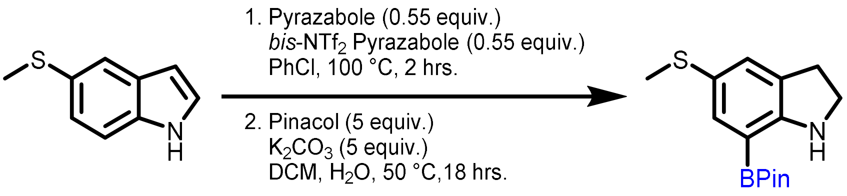

Compound **4j** was prepared following **General Procedure 1** with 5-(methylthio)indole (0.049 g, 0.040 mL). Reaction was heated to 100 °C for 2 hours to effect C7-borylation, then to 50 °C for 18 hours to convert to the BPin product. The product was isolated as a brown oil (0.054 g, 0.185 mmol, 62 %) with no further purification necessary.

^{1}H NMR (500 MHz, $CDCl_3$) δ 7.45 (m, 1H, ArC*H*), 7.21 (m, 1H, ArC*H*), 5.29 (br, 1H, N*H*), 3.63 (t, $^3J_{HH}$ = 8.5 Hz, 2H, H_2C-CH_2), 3.00 (t, $^3J_{HH}$ = 8.5 Hz, H_2C-CH_2), 2.40 (s, 3H, S-CH_3), 1.32 (s, 12H, O-C(CH_3)$_2$); **^{11}B NMR** (160 MHz, $CDCl_3$) δ 30.6 (s, Ar*B*Pin); **^{13}C{^{1}H} NMR** (126 MHz, $CDCl_3$) δ 156.79, 136.10, 130.27, 130.16, 124.69, 107.89 (br, *C*-B, only detectable *via* ^{1}H ^{13}C HMBC), 83.78, 47.16, 29.04, 25.06, 19.63 ppm.

HRMS (ESI$^+$) *m/z* calcd for $C_{15}H_{23}BNO_2S^+$ = 292.1537 [M+H]$^+$, found 292.1538.

2,3-Tetrahydrocyclopentyl 7-BPin Indoline (4k)

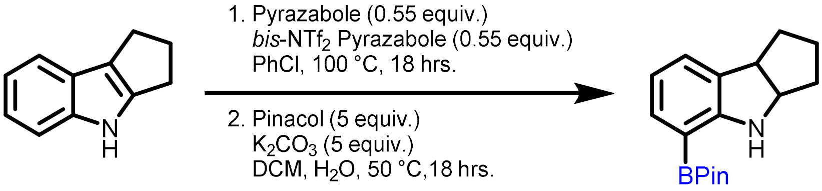

Compound **4k** was prepared following **General Procedure 1** with 1,2,3,4-tetrahydrocyclopent[b] indole (0.047 g). Reaction was stirred at room temperature for 1 hour (hydroboration step is slower for 2-substituted indoles), heated to 100 °C for 18 hours to effect C7-borylation, then to 50 °C for 18 hours to convert to the BPin product. The crude product was purified via reverse phase chromatography (4 g C18 column, celite dry load, 100% MeOH) to yield the product as a colourless oil (0.063 g, 0.221 mmol, 74%).

^{1}H NMR (500 MHz, $CDCl_3$) δ 7.36 (d, $^3J_{HH}$ = 7.5 Hz, 1H, ArC*H*), 7.08 (d, $^3J_{HH}$ = 7.0 Hz, 1H, ArC*H*), 6.56 (dd, $^3J_{HH}$ = 7.5, 7.0 Hz, 1H, ArC*H*), 5.03 (br, 1H, N*H*), 4.43 (br, 1H, HC(R)-C(R)*H*), 3.73 (br, 1H, *H*C(R)-C(R)H), 2.00-1.54 (m, 6H, HC-(CH_2)$_3$-CH), 1.35 s, 12H, O-C(CH$_3$)$_2$); **^{11}B NMR** (160 MHz, $CDCl_3$) δ 30.9 (s, Ar*B*Pin); **^{13}C{^{1}H} NMR** (126 MHz, $CDCl_3$) δ 158.34, 133.85, 132.24, 127.50, 116.05, 104.24 (br, *C*-B, only detectable *via* ^{1}H ^{13}C HMBC), 83.33, 63.14, 46.43, 36.82, 35.15, 25.17, 24.55, 24.55 ppm.

HRMS (ESI$^+$) *m/z* calcd for $C_{17}H_{25}BNO_2^+$ = 286.1973 [M+H]$^+$, found 286.1979.

3-tert-butyl-7-Bpin-Indoline (4l)

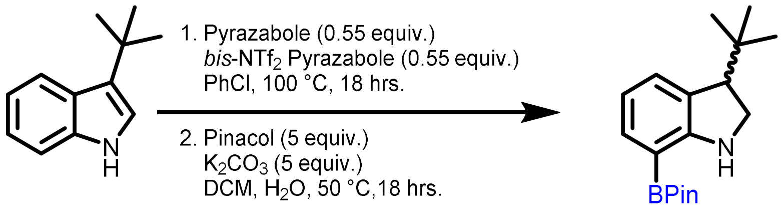

Compound **4l** was prepared following **General Procedure 1** with 3-tert-butylindole (0.052 g). Reaction was stirred at room temperature for 1 hour, heated to 100 °C for 18 hours to effect C7-borylation, then to 50 °C for 18 hours to convert to the BPin product. The product was isolated as a colorless oil (0.060 g, 0.199 mmol, 66 %) with no further purification necessary.

^{1}H NMR (500 MHz, $CDCl_3$) δ 7.39 (dt, J = 7.3, 1.0 Hz, 1H, ArC*H*), 7.24 (dt, *J* = 7.3, 1.2 Hz, 1H, ArC*H*), 6.57 (t, *J* = 7.3 Hz, 1H, ArC*H*), 5.06 (br, 1H, N*H*), 3.61 (t, *J* = 9.5 Hz, 1H), 3.50 (dd, *J* = 9.3, 5.6 Hz, 1H), 3.09 – 3.02 (m, 1H), 1.33 (s, 6H), 1.32 (s, 6H), 0.98 (s, 9H). **^{11}B NMR** (160 MHz, $CDCl_3$) δ 31.0. **^{13}C{^{1}H} NMR** (126 MHz, $CDCl_3$) δ 159.69, 134.04, 129.67, 129.38, 116.08, 105.86 (br, C-B, only detectable via ^{1}H-^{13}C HMBC) 83.44, 52.16, 49.26, 33.97, 27.65, 25.22, 25.02.

HRMS (ESI$^+$) *m/z* calcd for $C_{18}H_{29}BNO_2^+$ = 302.2286 [M+H]$^+$, found 302.2279.

3.3 NMR Spectra of Synthesised 7-BPin Indolines

A number of substrates have minor impurities in the ^{11}B NMR spectra, consistent with pyrazabole species. These are all less than 5% based on ^{11}B NMR spectroscopy and higher purity can be obtained for these substrates, if required, by column chromatography.

NMR Spectra of 7-BPin Indoline (**4a**):

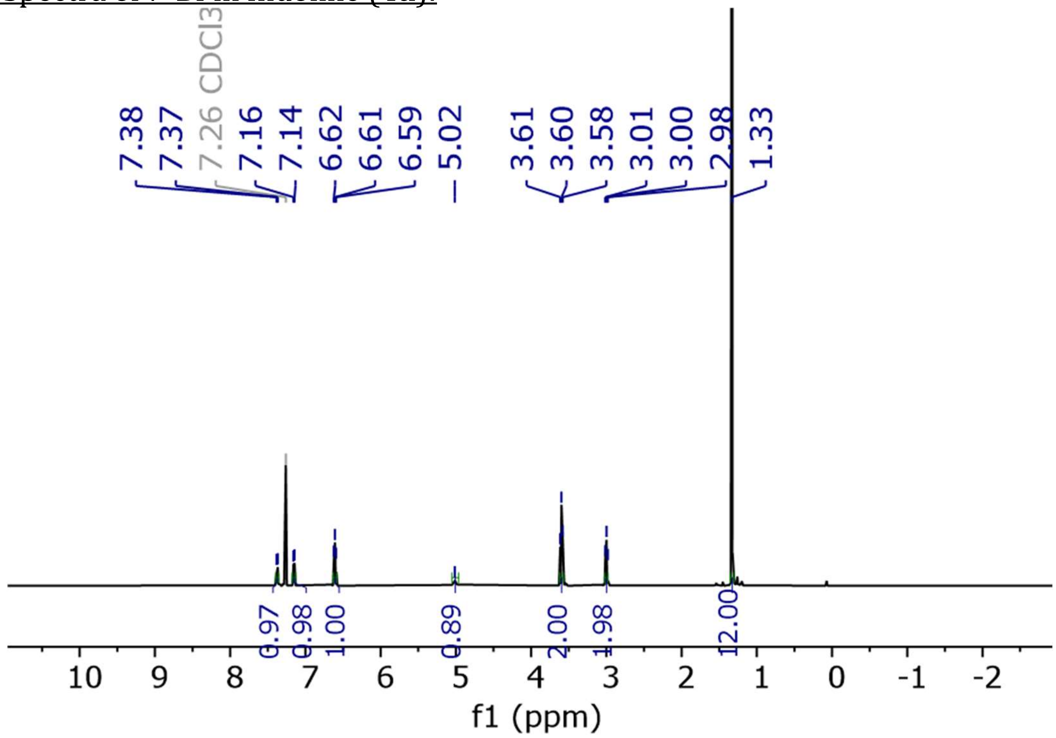

Figure S20: ^{1}H NMR spectrum of 7-BPin indoline (**4a**) in $CDCl_3$.

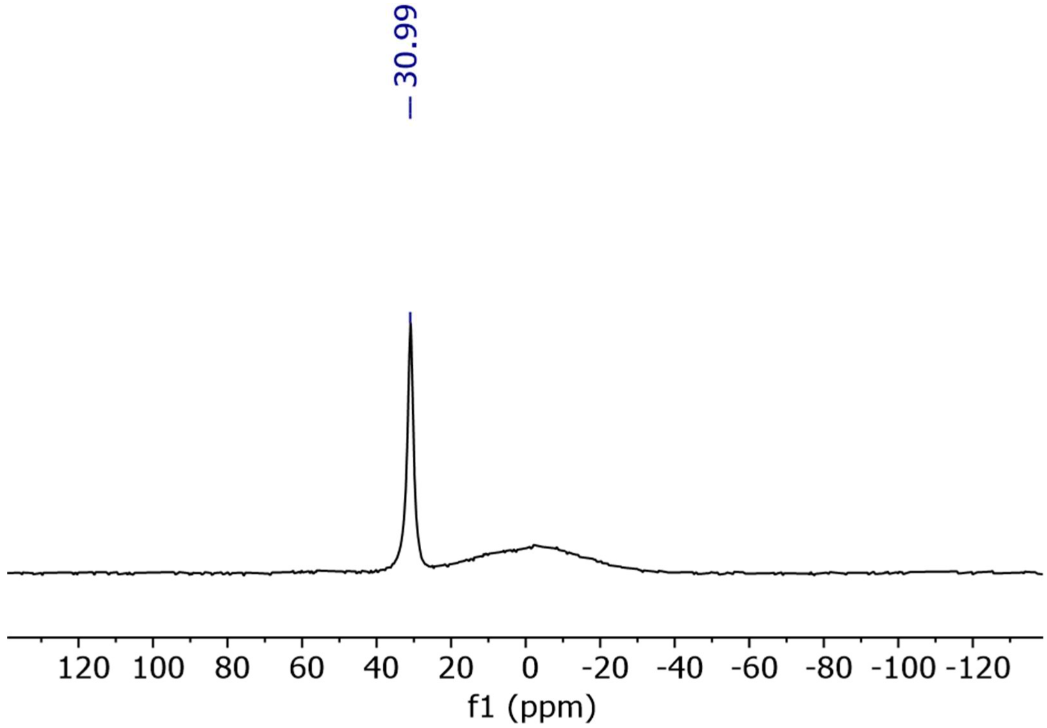

Figure S21: ^{11}B NMR spectrum of 7-BPin indoline (**4a**) in $CDCl_3$.

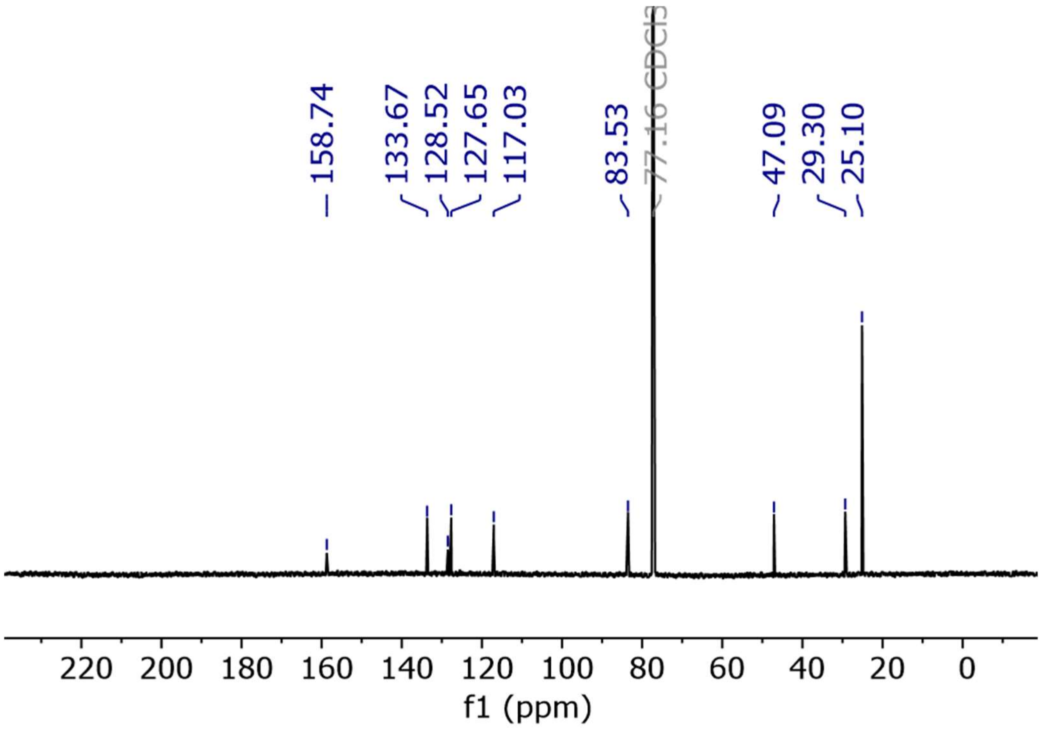

Figure S22: $^{13}C\{^{1}H\}$ NMR spectrum of 7-BPin indoline (**4a**) in $CDCl_3$.

<u>NMR Spectra of 5-Methyl 7-BPin Indoline (**4b**)</u>

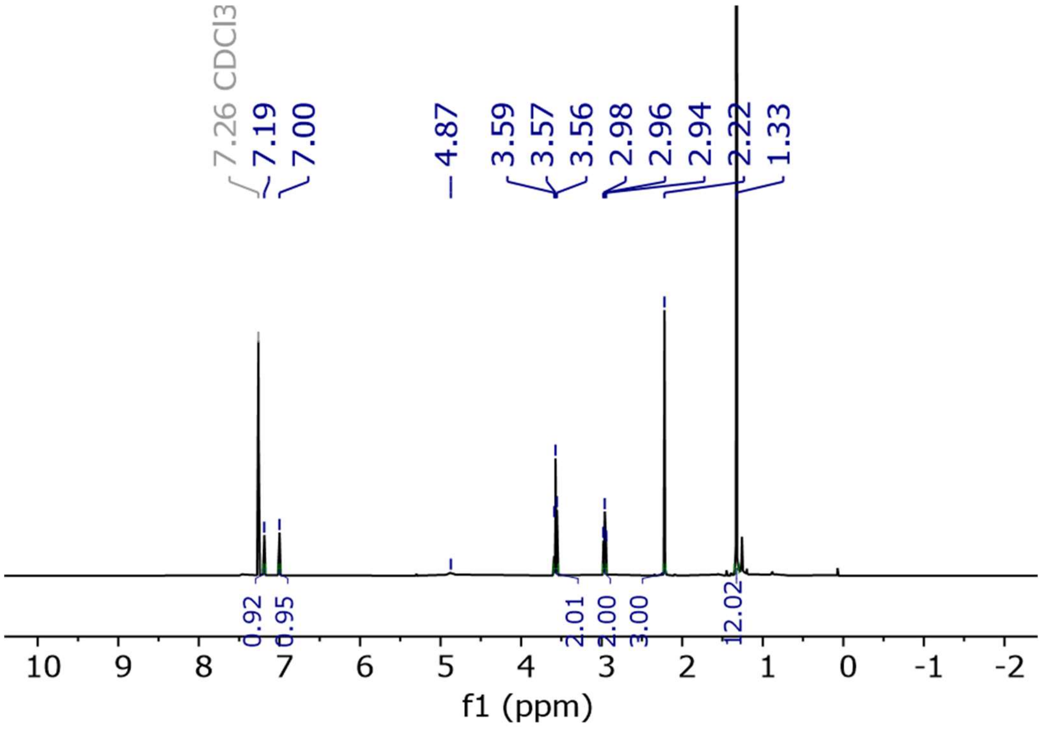

Figure S23: ^{1}H NMR spectrum of 5-Methyl 7-BPin indoline (**4b**) in $CDCl_3$.

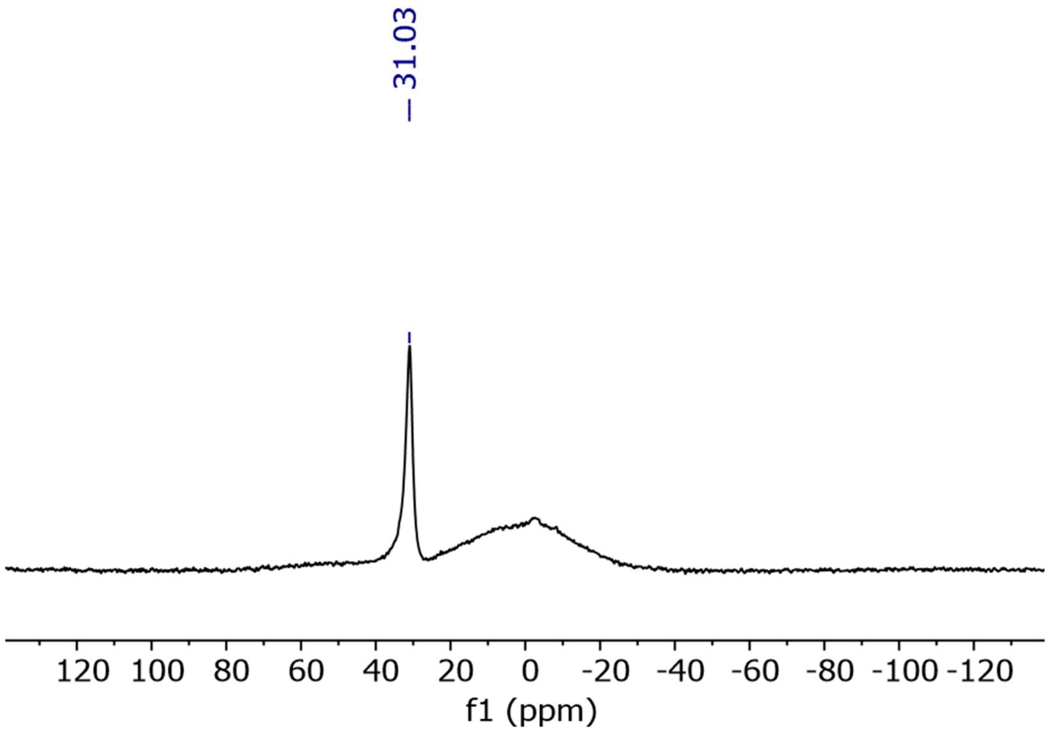

Figure S24: ^{11}B NMR spectrum of 5-Methyl 7-BPin indoline (**4b**) in $CDCl_3$.

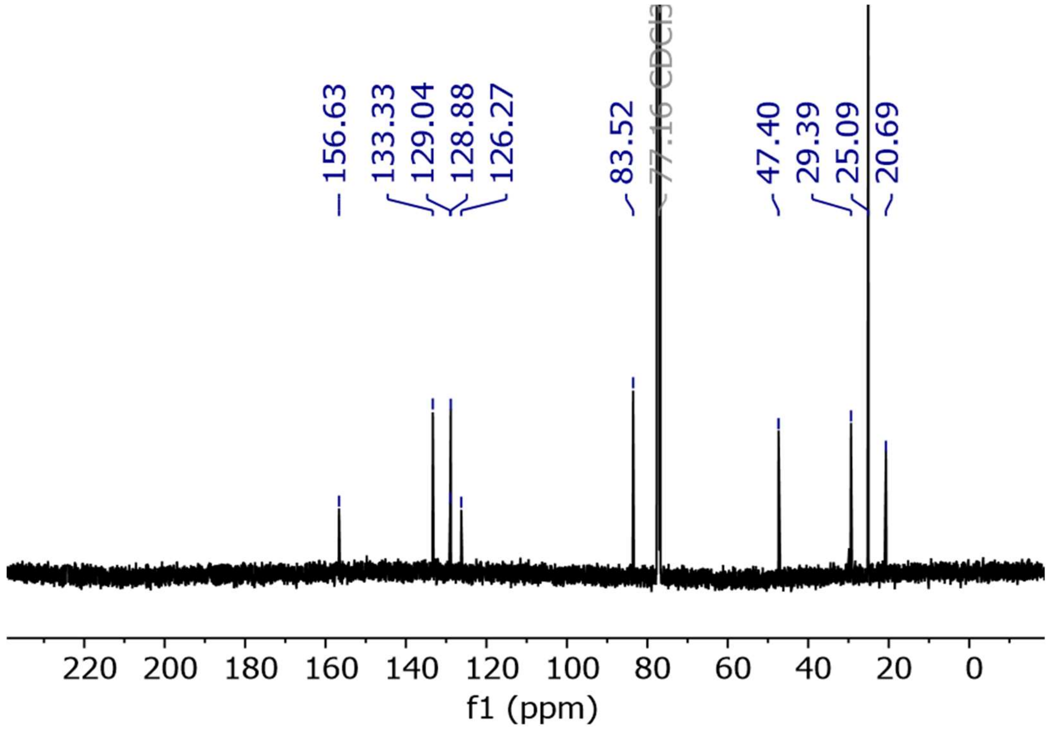

Figure S25: $^{13}C\{^1H\}$ NMR spectrum of 5-Methyl 7-BPin indoline (**4b**) in $CDCl_3$.

NMR Spectra of 3-Methyl 7-BPin Indoline (**4c**)

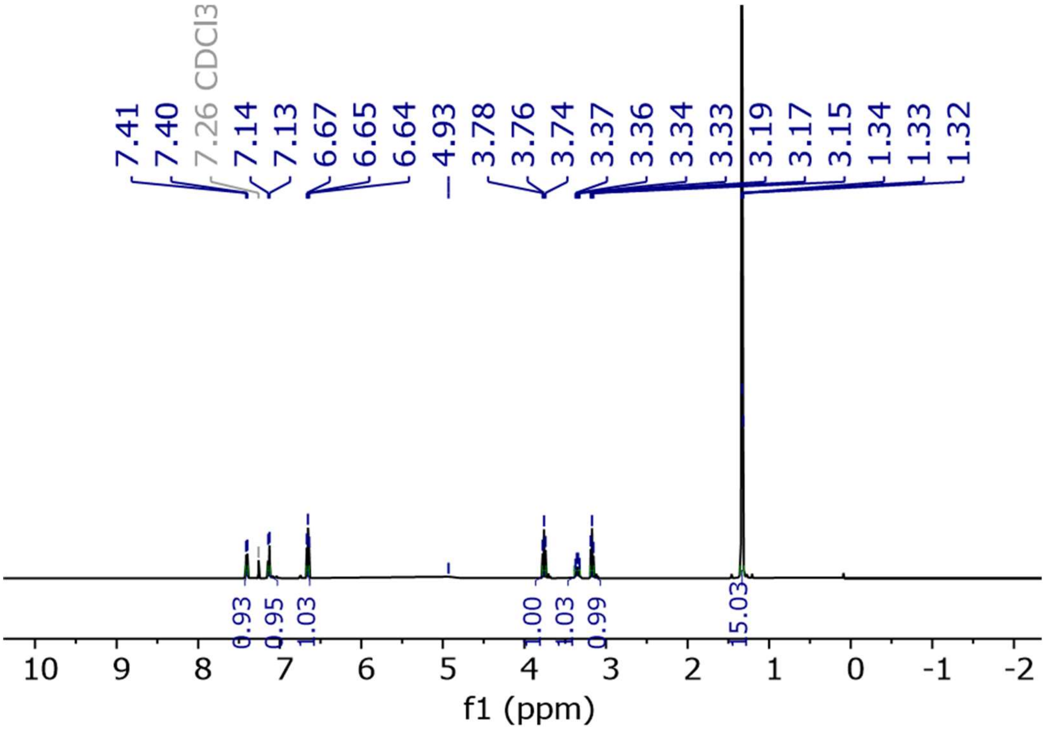

Figure S26: ^{1}H NMR spectrum of 3-Methyl 7-BPin indoline (**4c**) in $CDCl_3$.

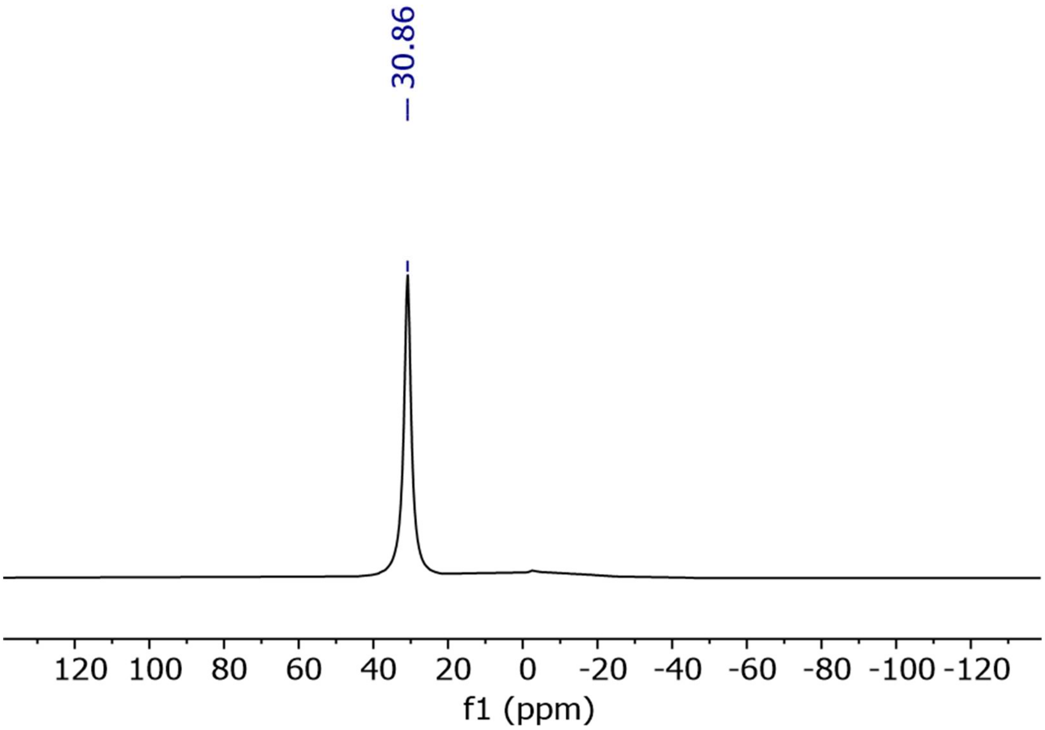

Figure S27: ^{11}B NMR spectrum of 3-Methyl 7-BPin indoline (**4c**) in $CDCl_3$.

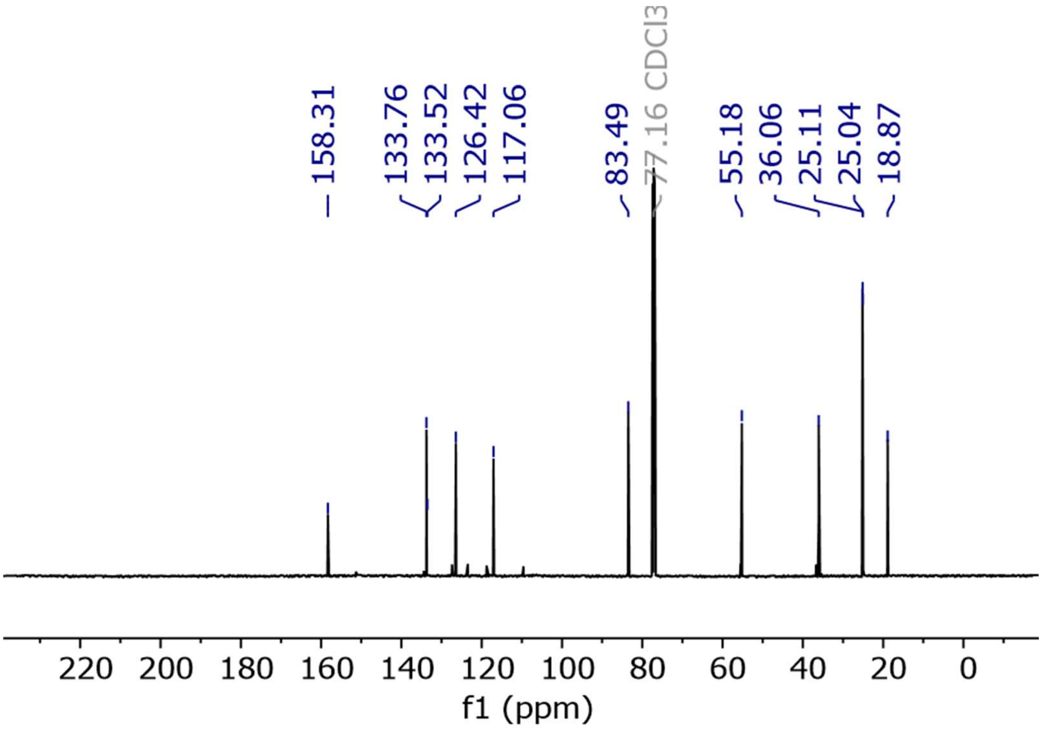

Figure S28: $^{13}C\{^1H\}$ NMR spectrum of 3-Methyl 7-BPin indoline (**4c**) in $CDCl_3$.

<u>NMR Spectra of 2-Methyl 7-BPin Indoline (**4d**)</u>

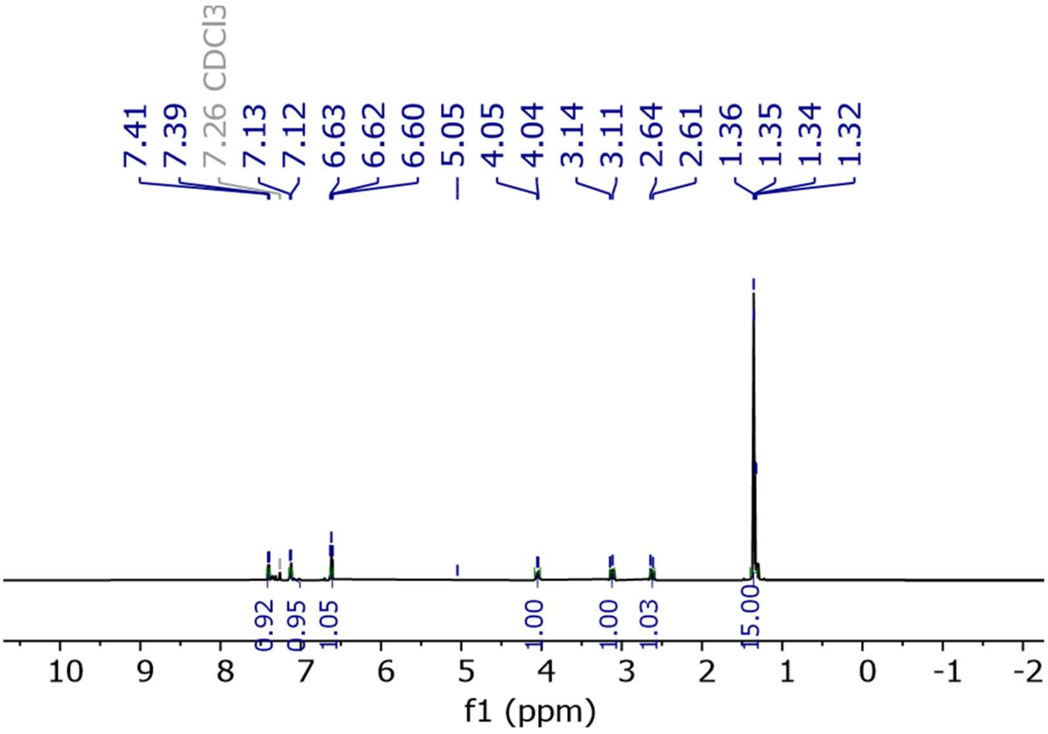

Figure S29: ^1H NMR spectrum of 2-Methyl 7-BPin indoline (**4d**) in $CDCl_3$.

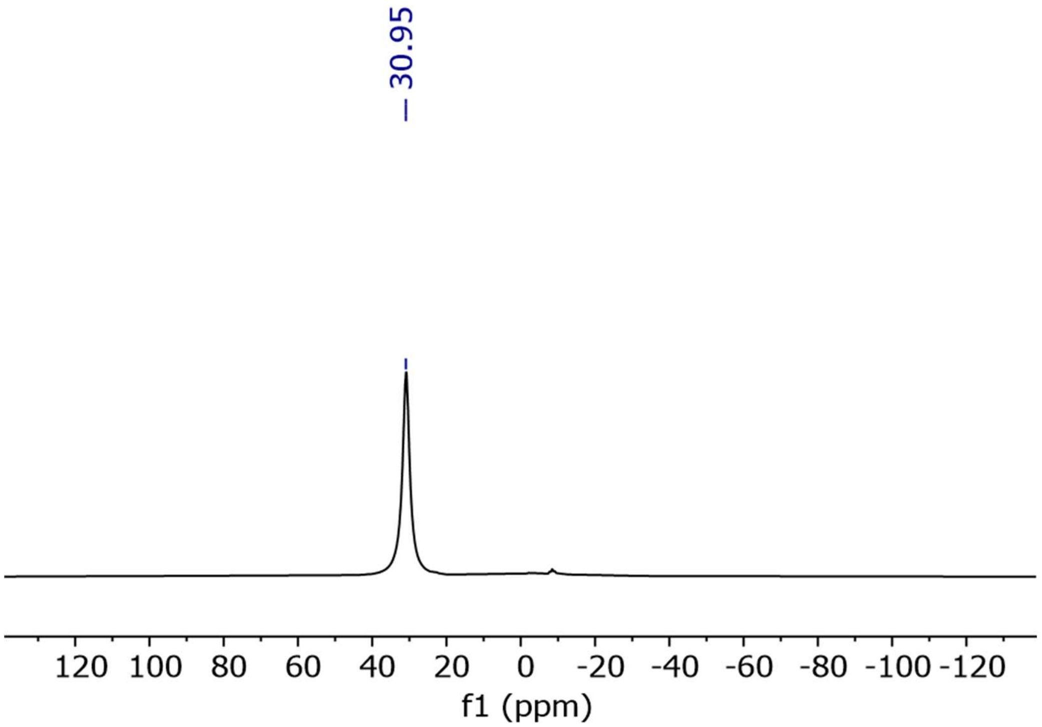

Figure S30: ^{11}B NMR spectrum of 2-Methyl 7-BPin indoline (**4d**) in $CDCl_3$.

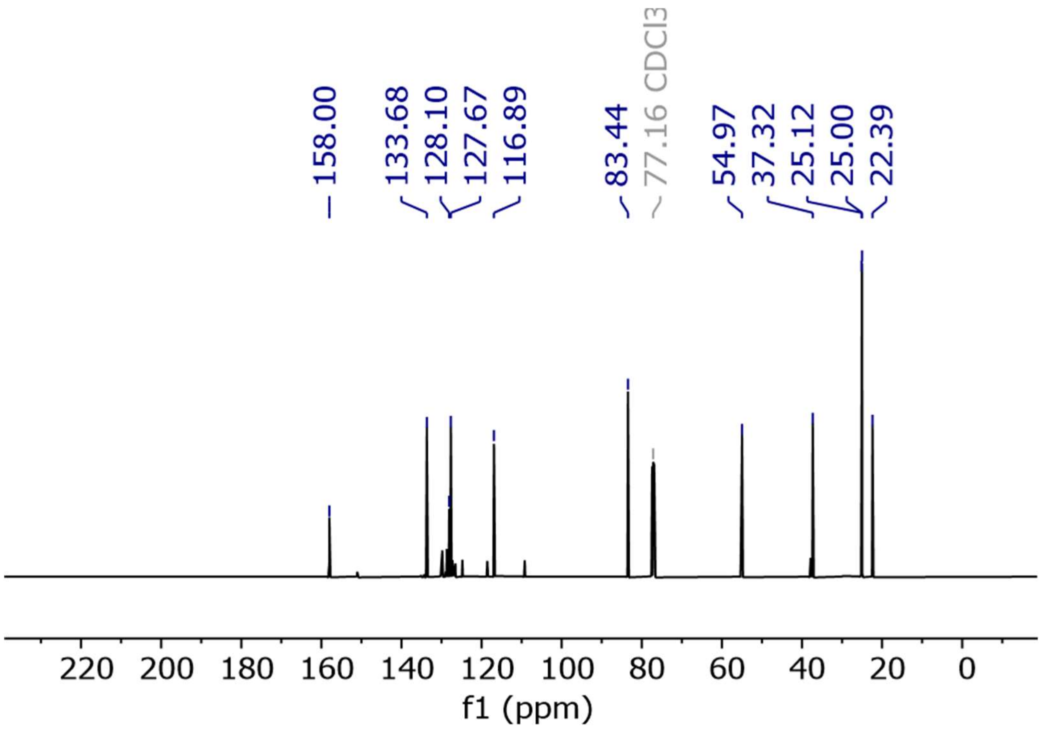

Figure S31: $^{13}C\{^1H\}$ NMR spectrum of 2-Methyl 7-BPin indoline (**4d**) in $CDCl_3$.

NMR Spectra of 6-Methyl 7-BPin Indoline (**4e**)

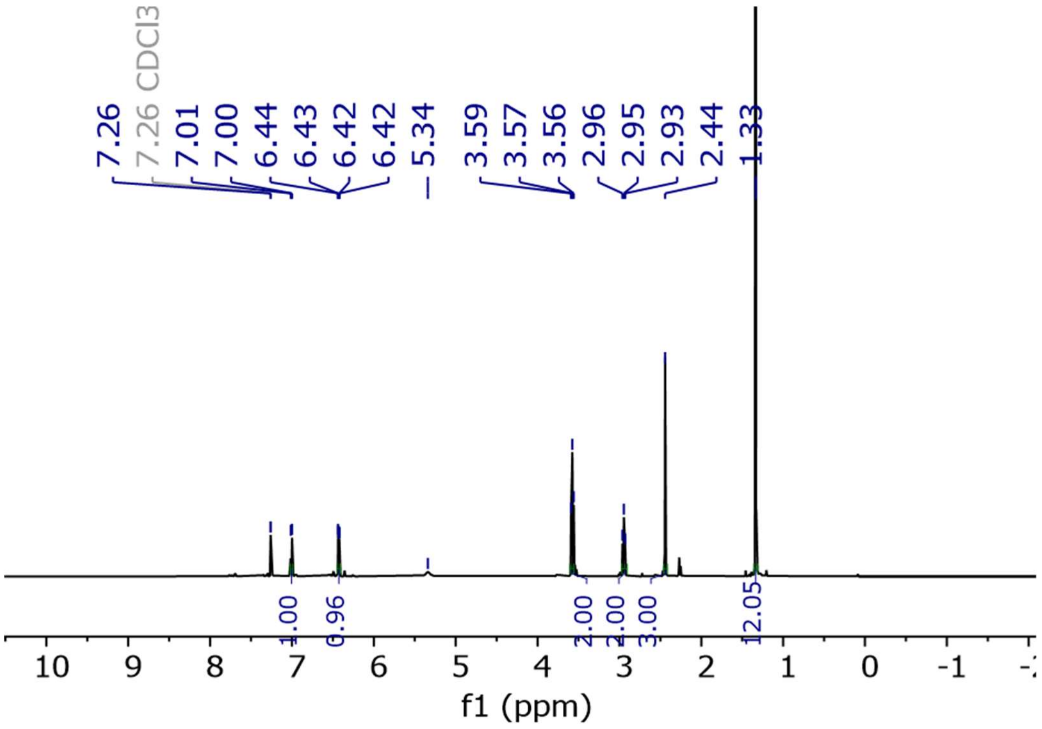

Figure S32: ^{1}H NMR spectrum of 6-Methyl 7-BPin indoline (**4e**) in $CDCl_3$.

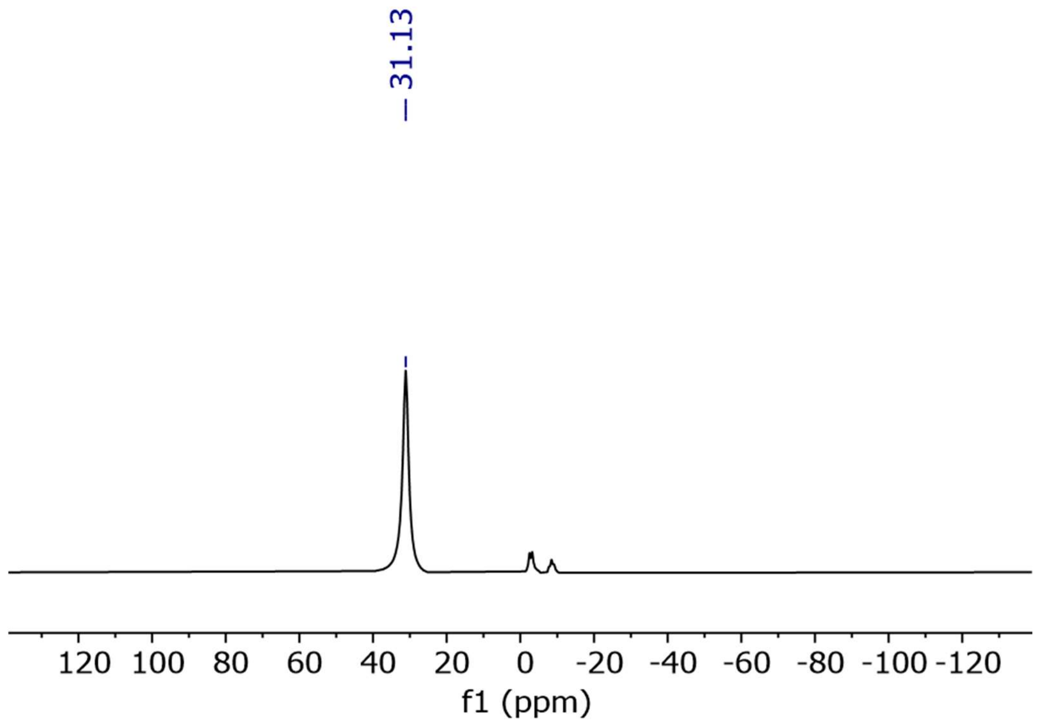

Figure S33: ^{11}B NMR spectrum of 6-Methyl 7-BPin indoline (**4e**) in $CDCl_3$.

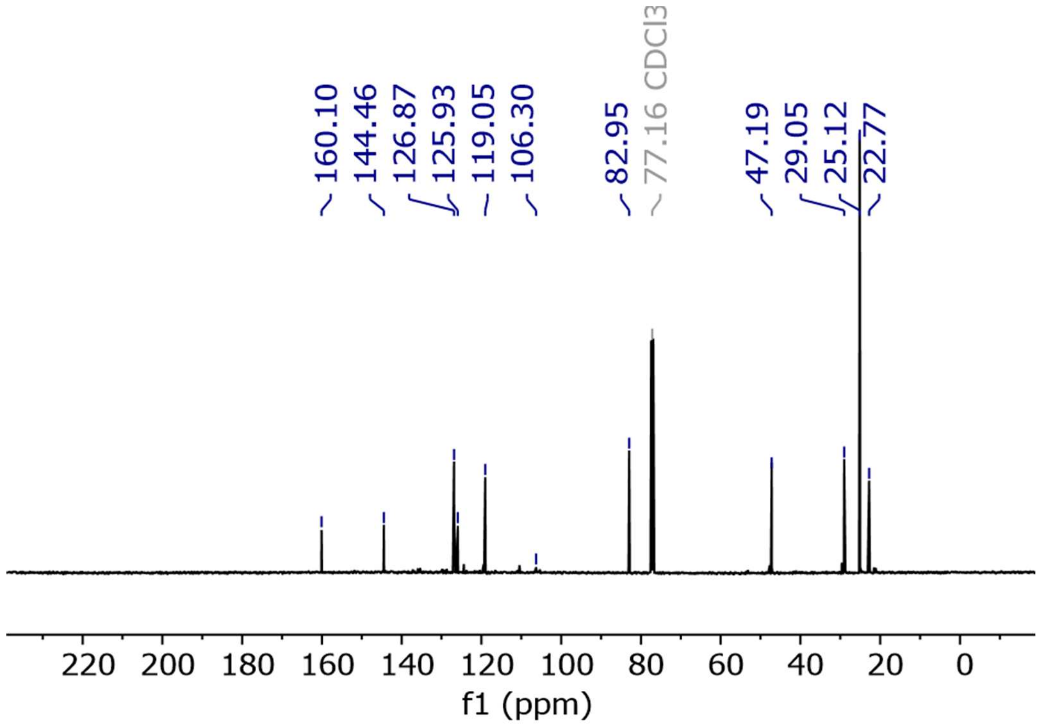

Figure S34: $^{13}C\{^1H\}$ NMR spectrum of 6-Methyl 7-BPin indoline (**4e**) in $CDCl_3$.

<u>NMR Spectra of 5-Chloro 7-BPin Indoline (**4f**)</u>

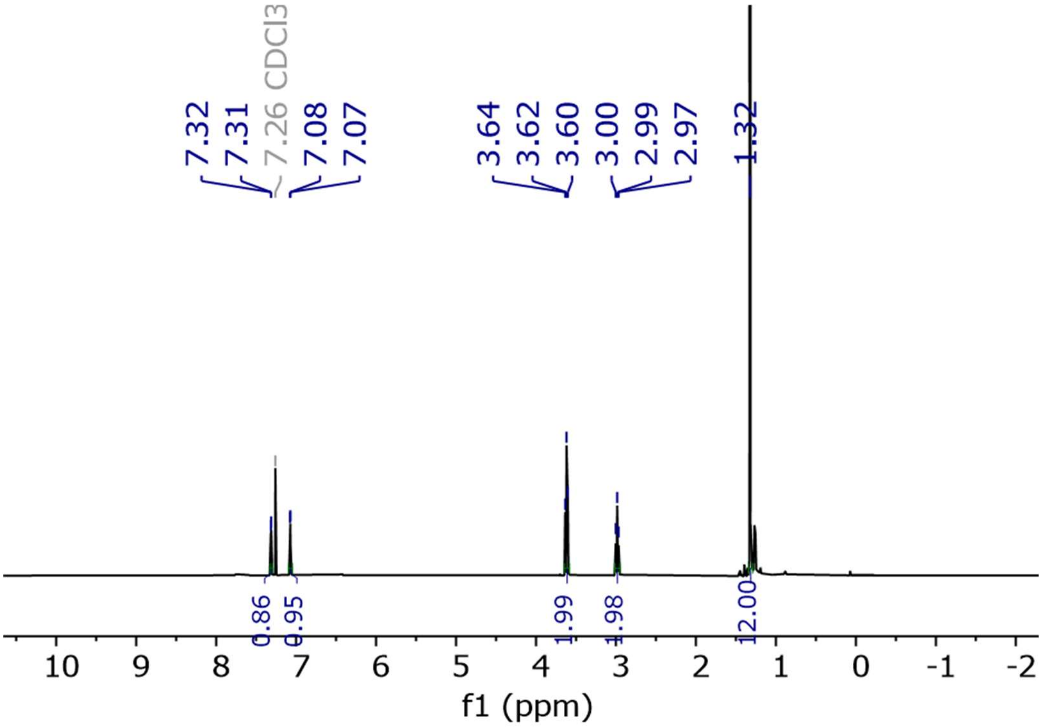

Figure S35: ^1H NMR spectrum of 5-Chloro 7-BPin indoline (**4f**) in $CDCl_3$.

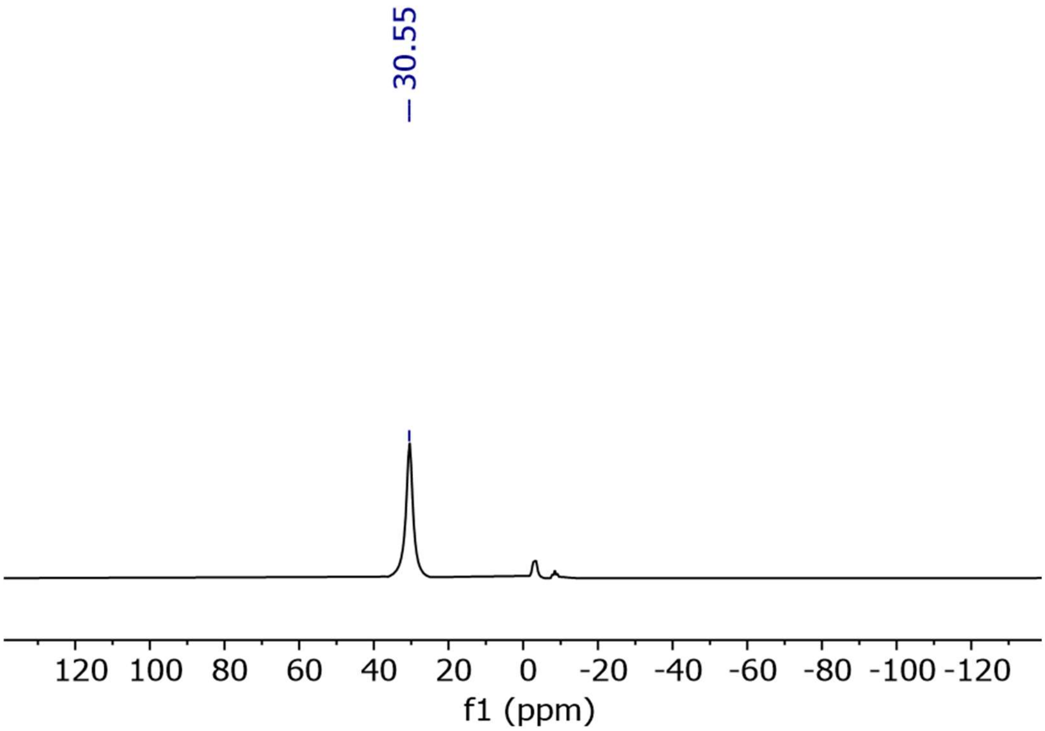

Figure S36: ^{11}B NMR spectrum of 5-Chloro 7-BPin indoline (**4f**) in $CDCl_3$.

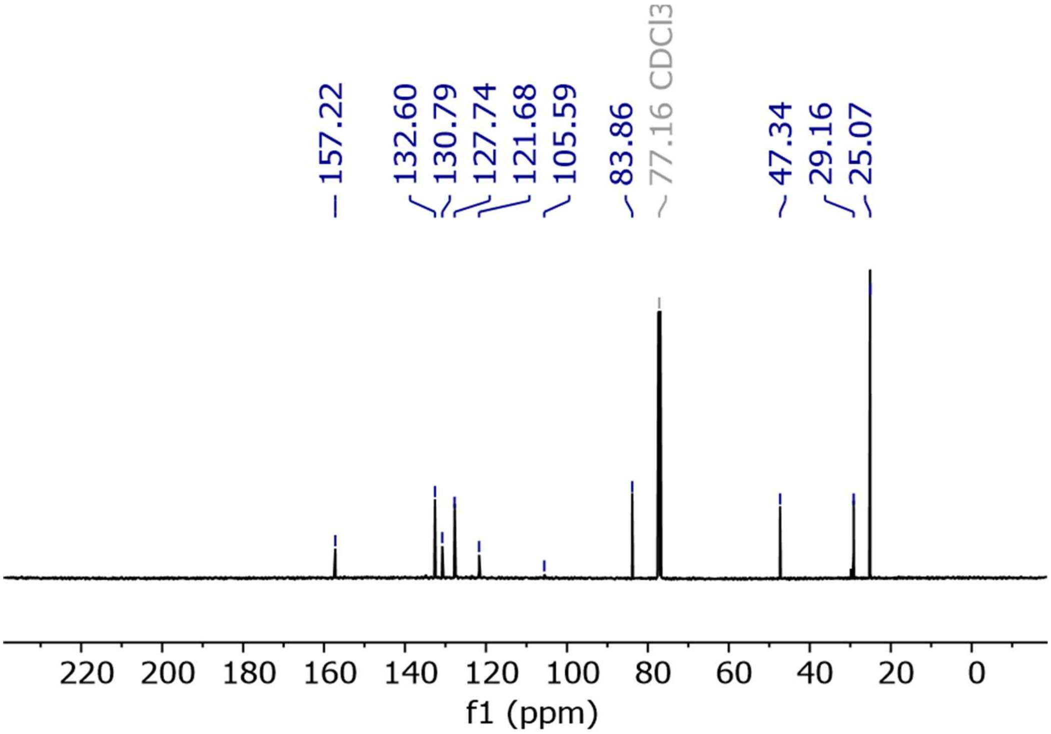

Figure S37: $^{13}C\{^1H\}$ NMR spectrum of 5-Chloro 7-BPin indoline (**4f**) in $CDCl_3$.

NMR Spectra of 4-Fluoro 7-BPin Indoline (**4g**)

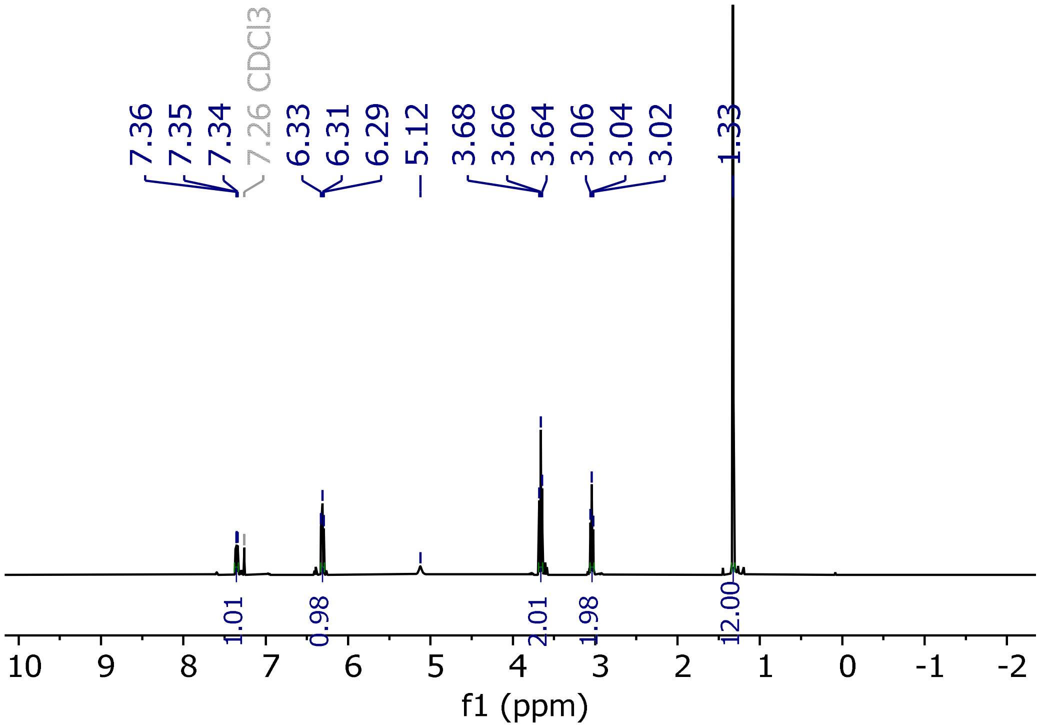

Figure S38: ^{1}H NMR spectrum of 4-Fluoro 7-BPin indoline (**4g**) in $CDCl_3$.

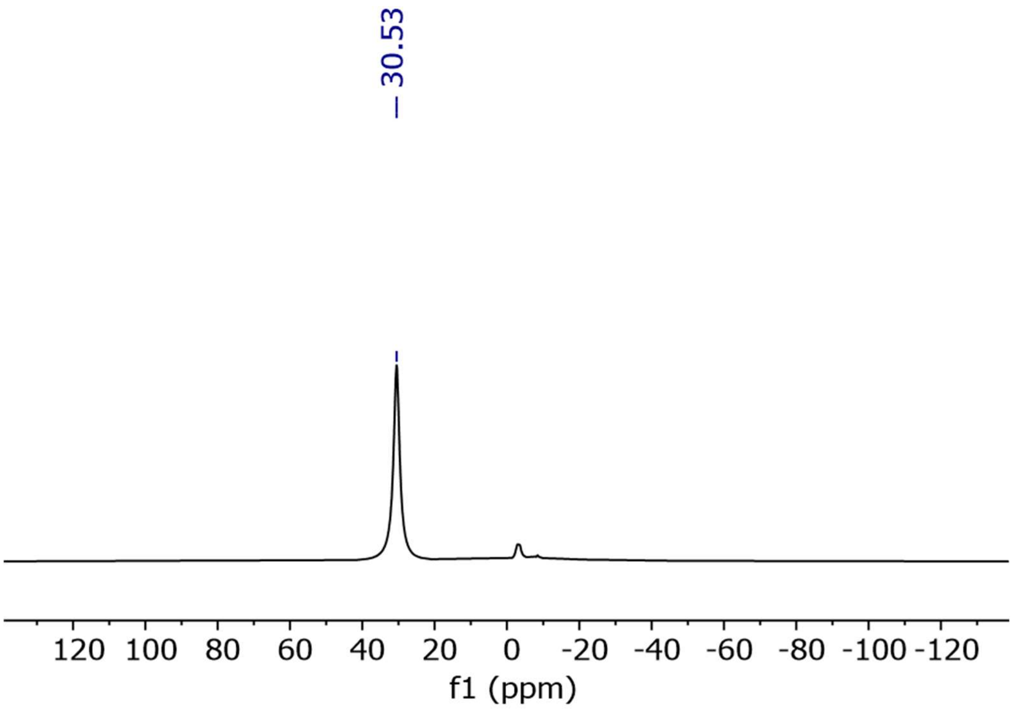

Figure S39: ^{11}B NMR spectrum of 4-Fluoro 7-BPin indoline (**4g**) in $CDCl_3$.

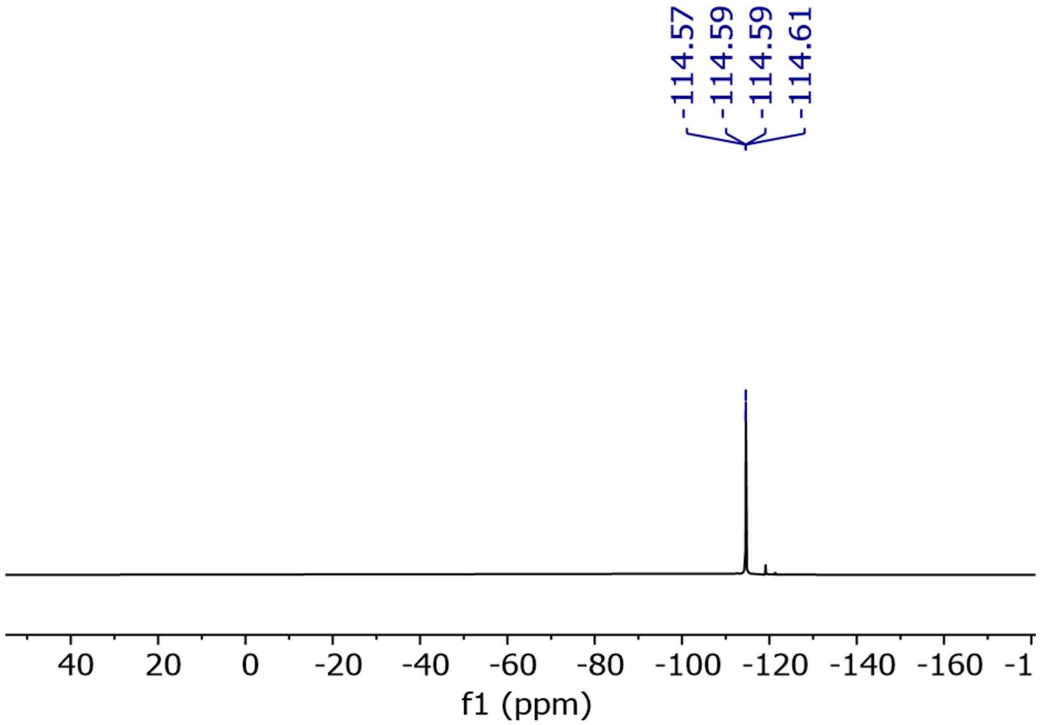

Figure S40: ^{19}F NMR spectrum of 4-Fluoro 7-BPin indoline (**4g**) in $CDCl_3$.

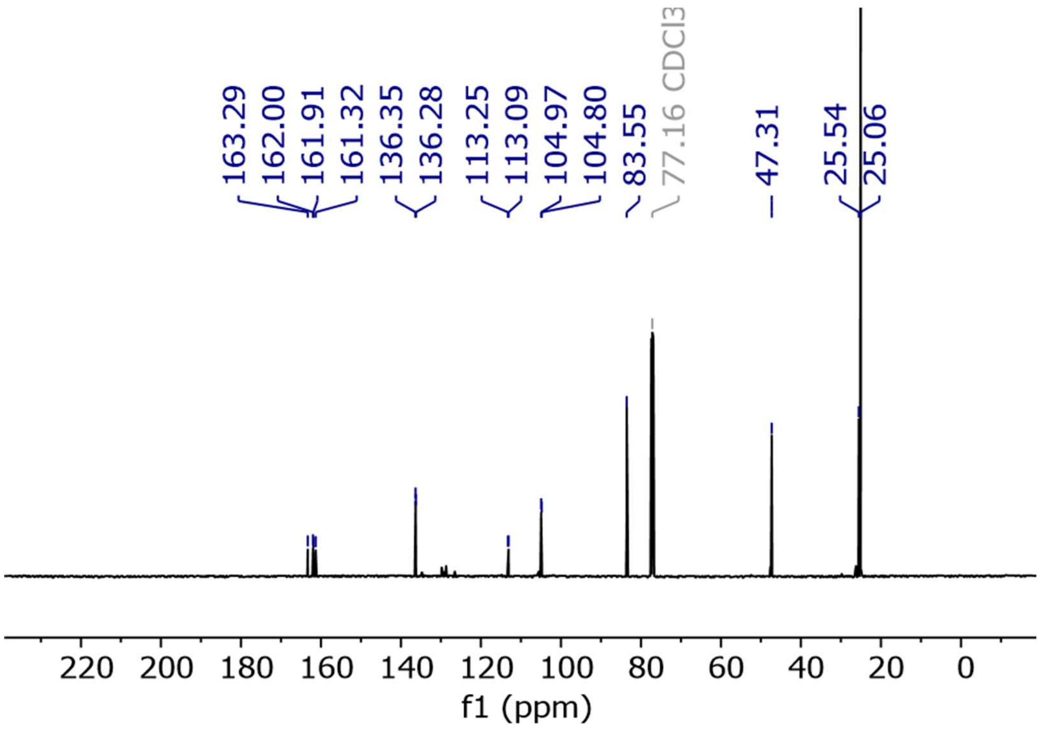

Figure S41: $^{13}C\{^{1}H\}$ NMR spectrum of 4-Fluoro 7-BPin indoline (**4g**) in $CDCl_3$.

NMR Spectra of 4-Bromo 7-BPin Indoline (**4h**)

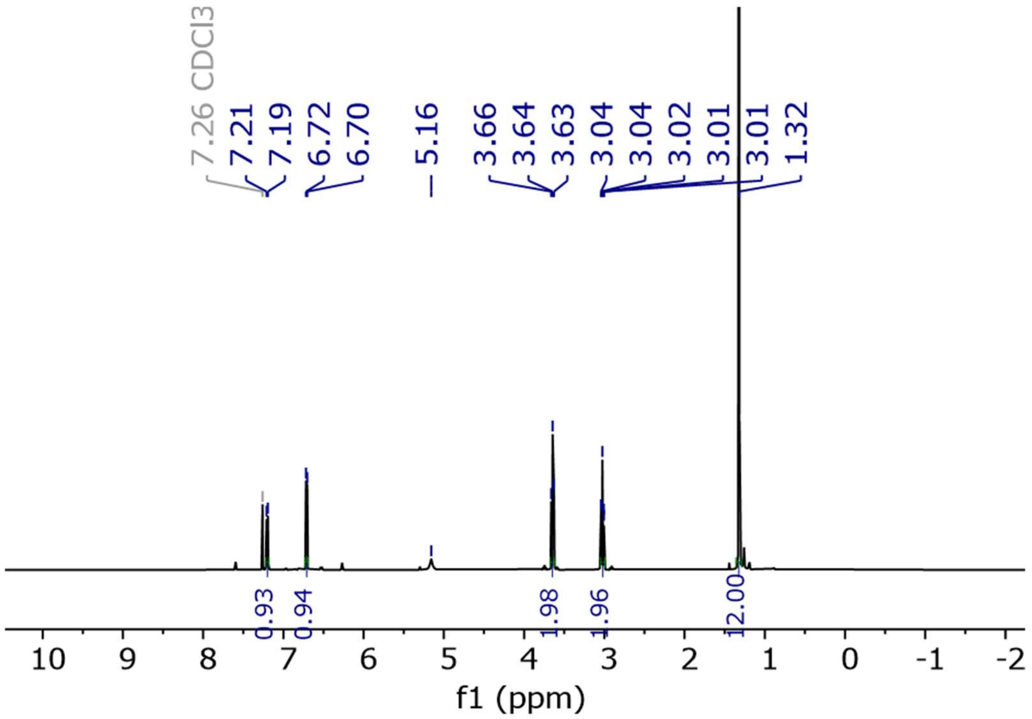

Figure S42: ^{1}H NMR spectrum of 4-Bromo 7-BPin indoline (**4h**) in $CDCl_3$.

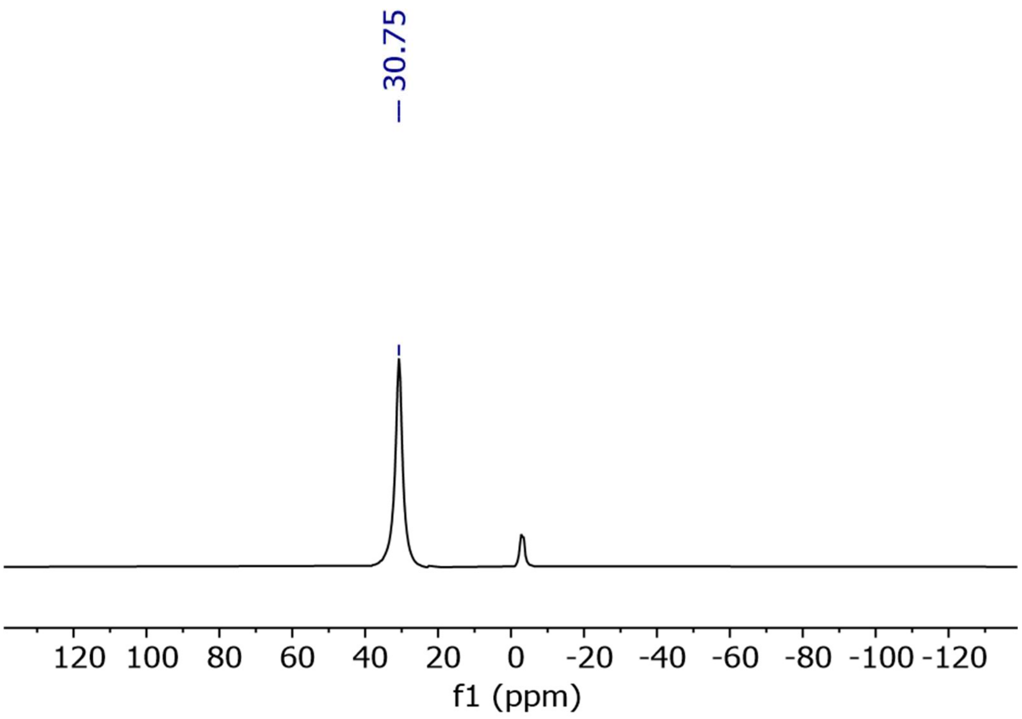

Figure S43: ^{11}B NMR spectrum of 4-Bromo 7-BPin indoline (**4h**) in $CDCl_3$.

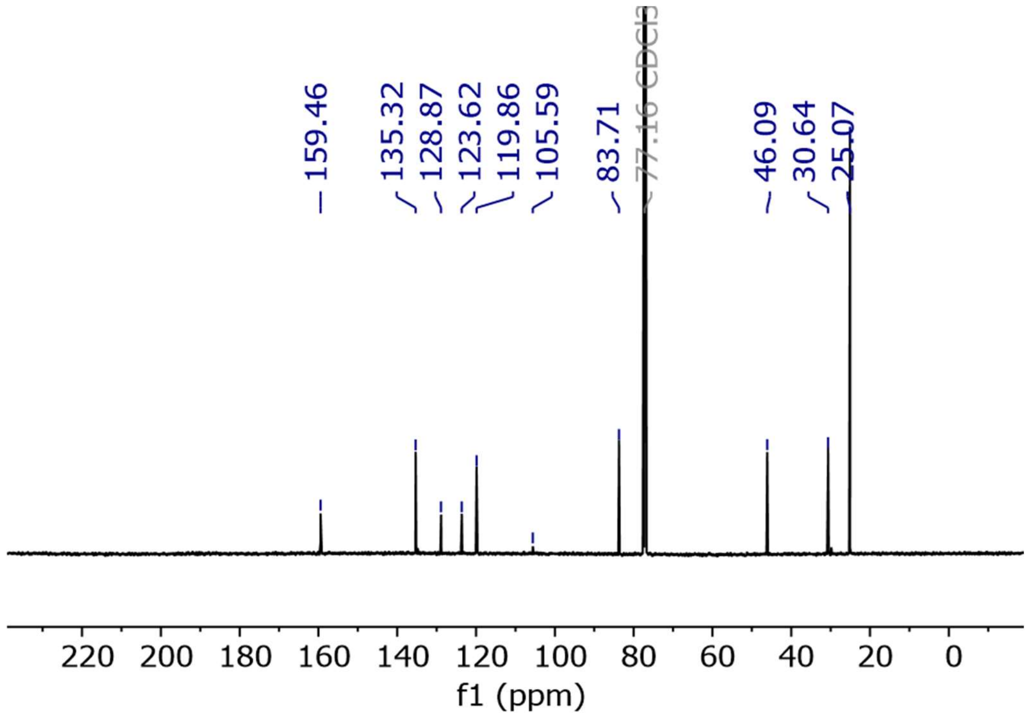

Figure S44: $^{13}C\{^{1}H\}$ NMR spectrum of 4-Bromo 7-BPin indoline (**4h**) in $CDCl_3$.

NMR Spectra of 5-Methoxy 7-BPin Indoline (**4i**)

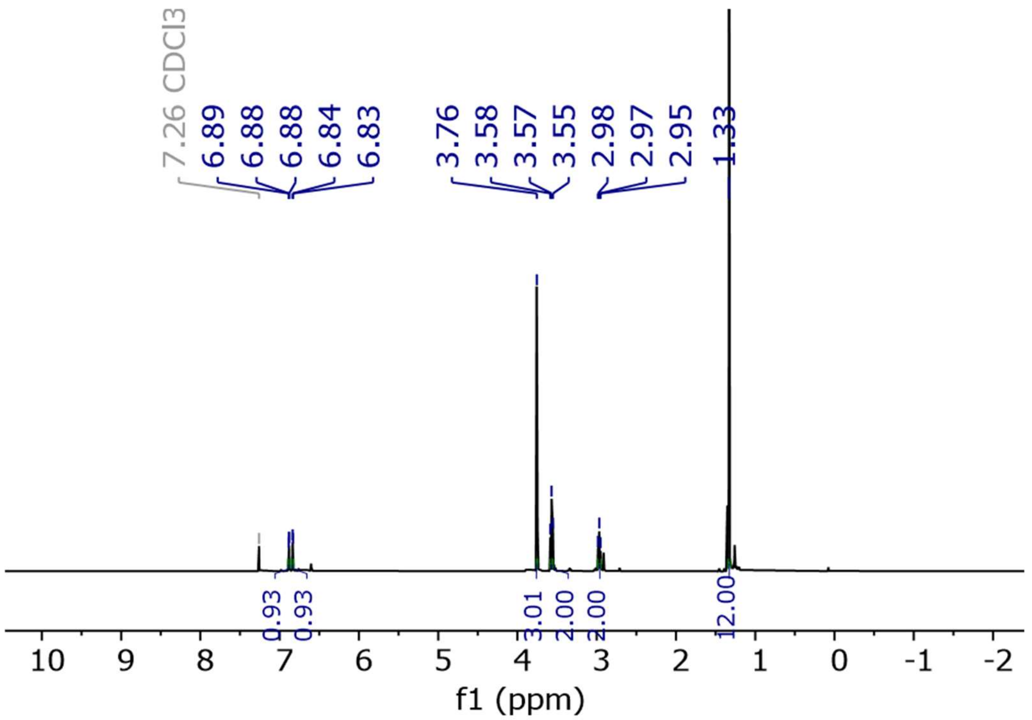

Figure S45: ^{1}H NMR spectrum of 5-Methoxy 7-BPin indoline (**4i**) in $CDCl_3$.

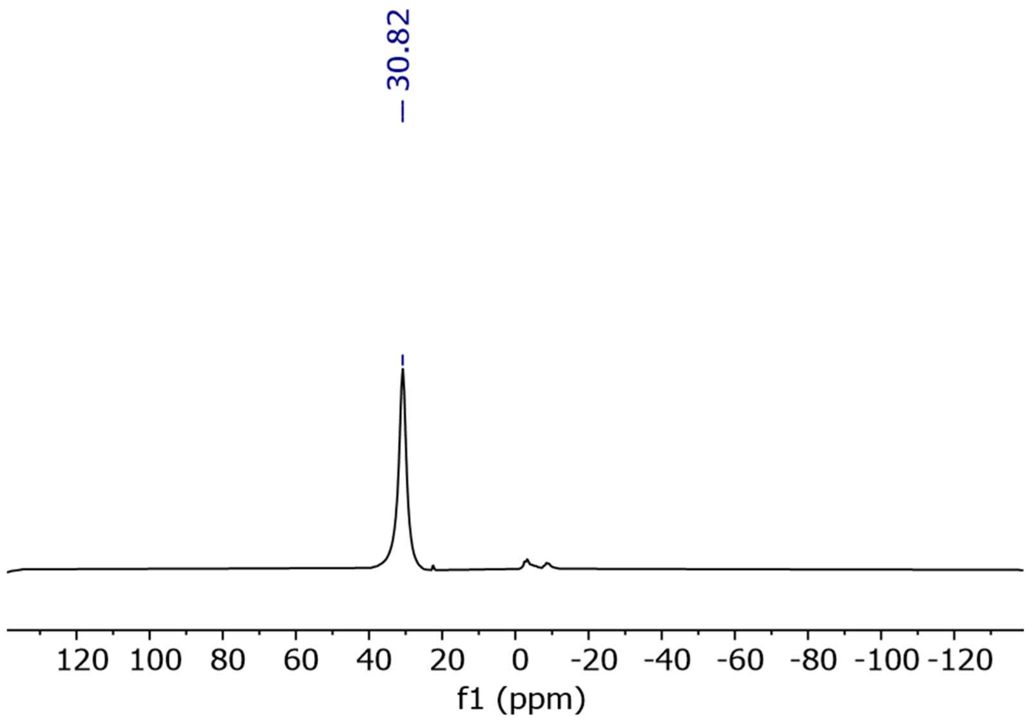

Figure S46: ^{11}B NMR spectrum of 5-Methoxy 7-BPin indoline (**4i**) in $CDCl_3$.

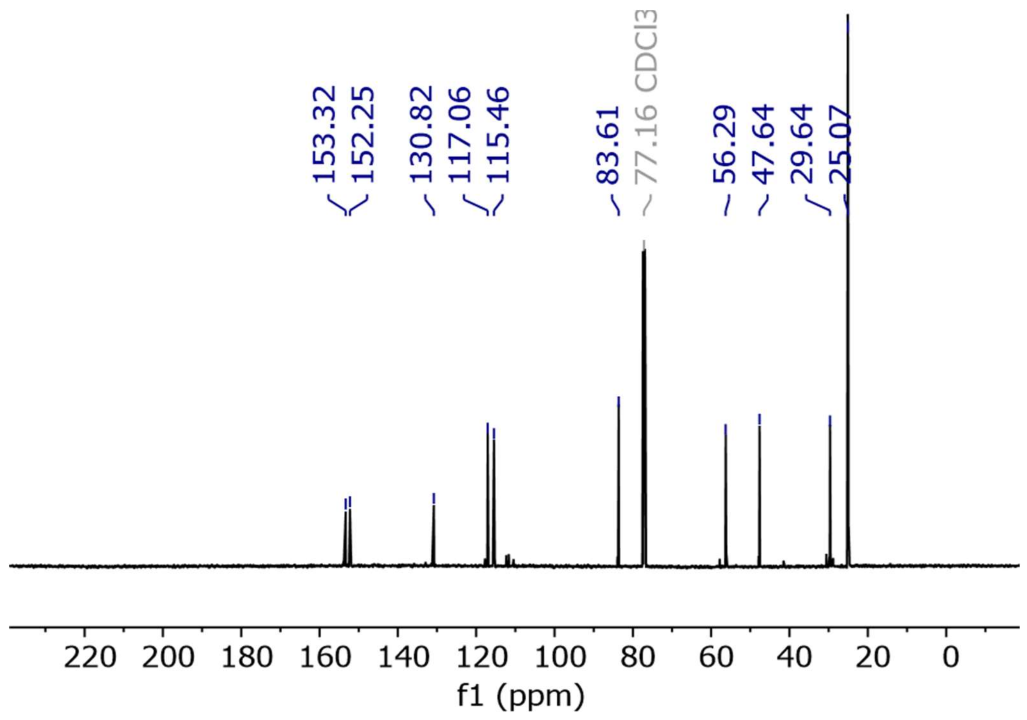

Figure S47: $^{13}C\{^1H\}$ NMR spectrum of 5-Methoxy 7-BPin indoline (**4i**) in $CDCl_3$.

NMR Spectra of 5-Methylthio 7-BPin Indoline (**4j**)

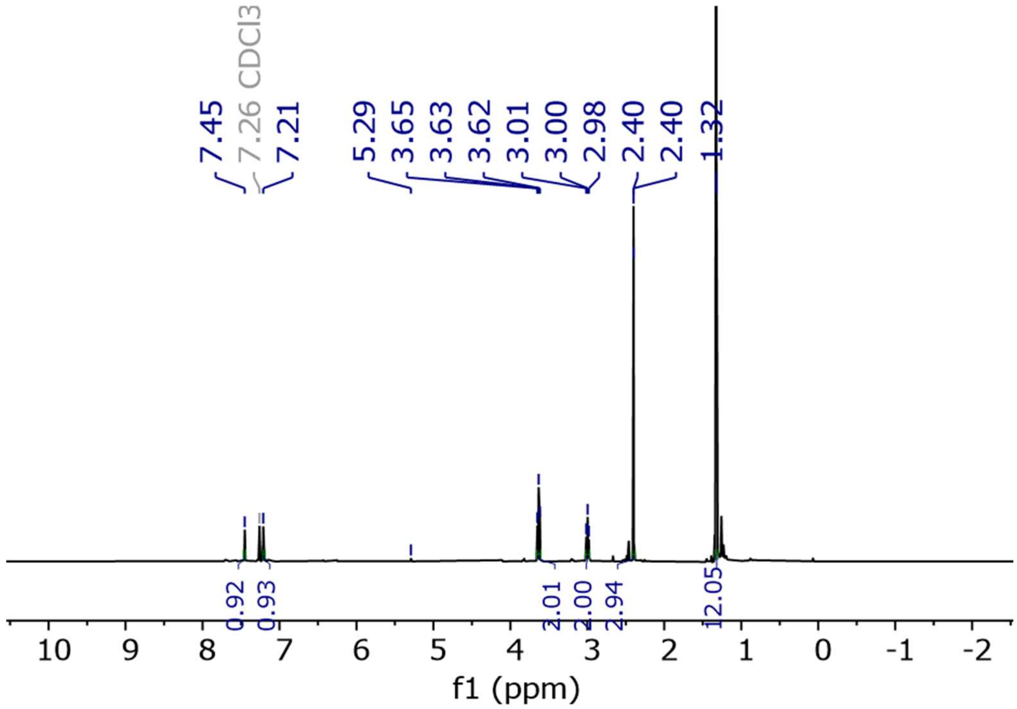

Figure S48: ^{1}H NMR spectrum of 5-Methylthio 7-BPin indoline (**4j**) in $CDCl_3$.

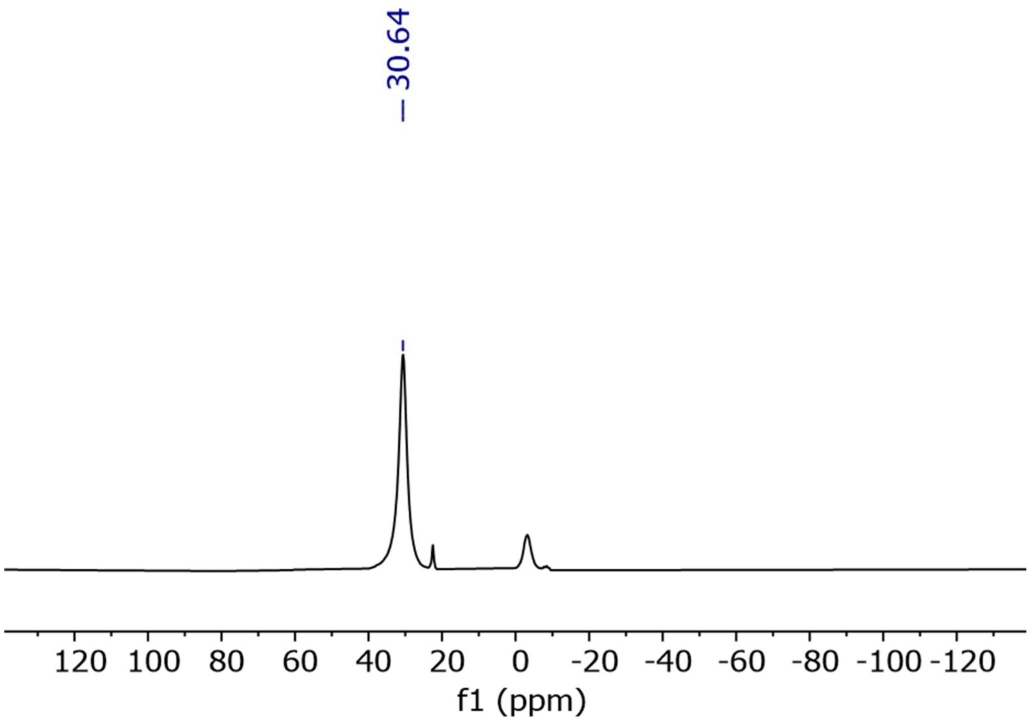

Figure S49: ^{11}B NMR spectrum of 5-Methylthio 7-BPin indoline (**4j**) in $CDCl_3$.

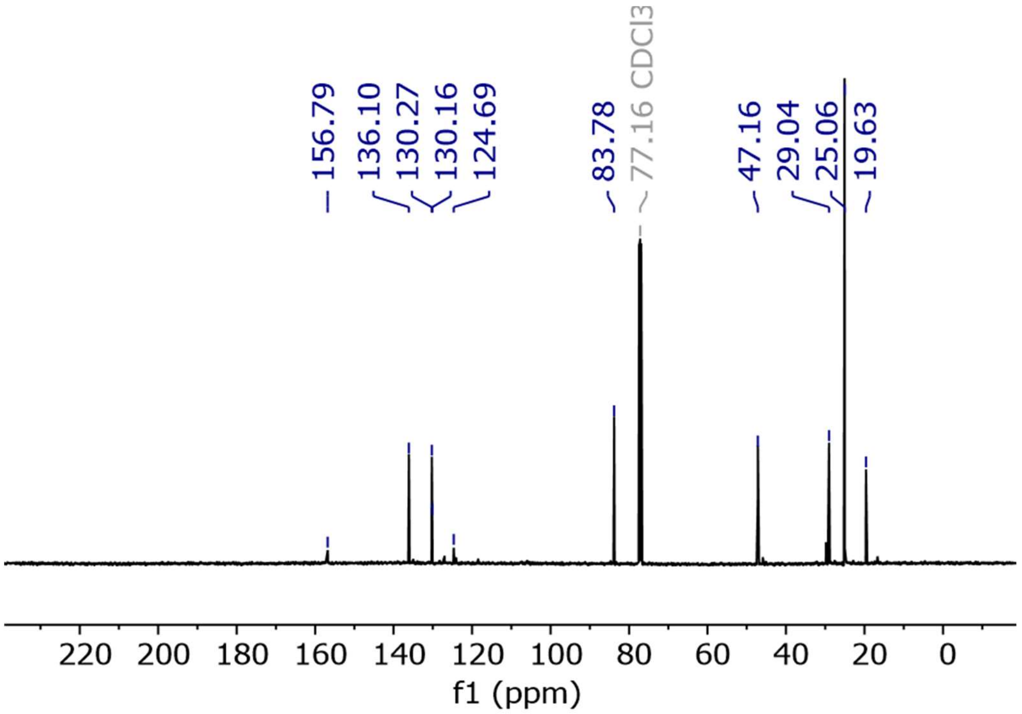

Figure S50: $^{13}C\{^{1}H\}$ NMR spectrum of 5-Methylthio 7-BPin indoline (**4j**) in $CDCl_3$.

<u>NMR Spectra of 2,3-Tetrahydrocyclopentyl 7-BPin Indoline (**4k**)</u>

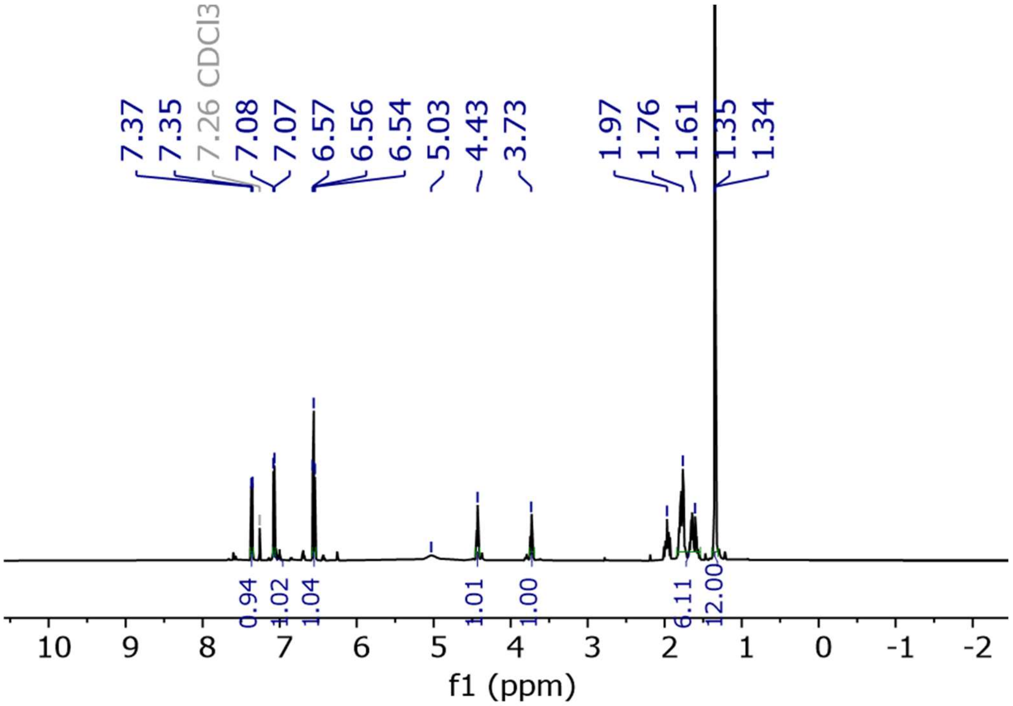

Figure S51: ^{1}H NMR spectrum of 2,3-Tetrahydrocyclopentyl 7-BPin indoline (**4k**) in $CDCl_3$.

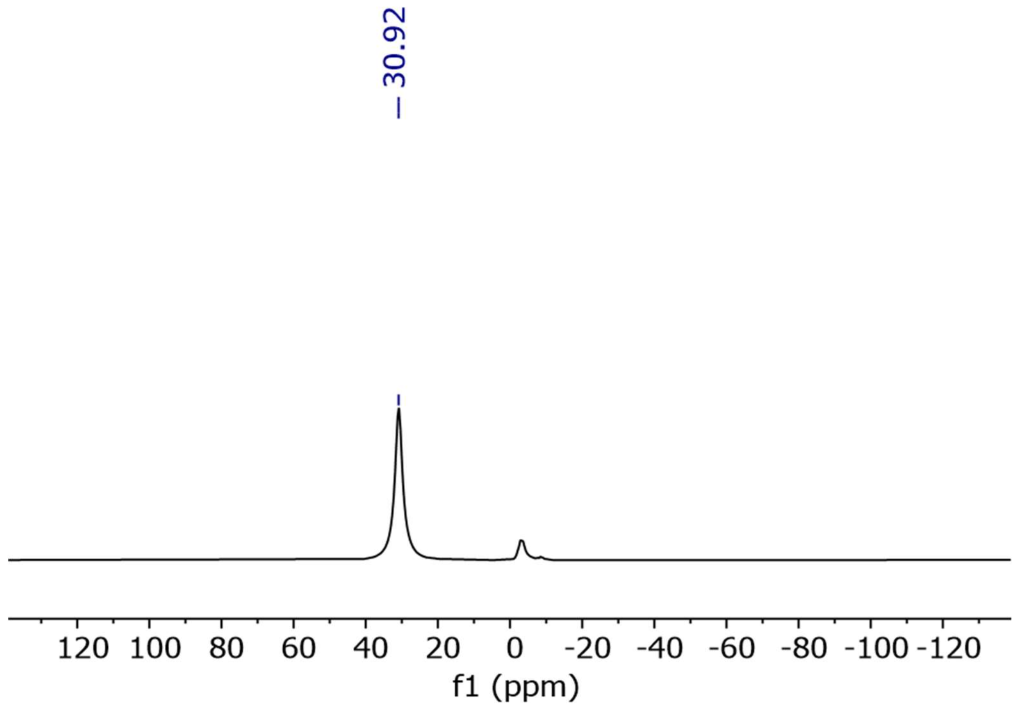

Figure S52: ^{11}B NMR spectrum of 2,3-Tetrahydrocyclopentyl 7-BPin indoline (**4k**) in $CDCl_3$.

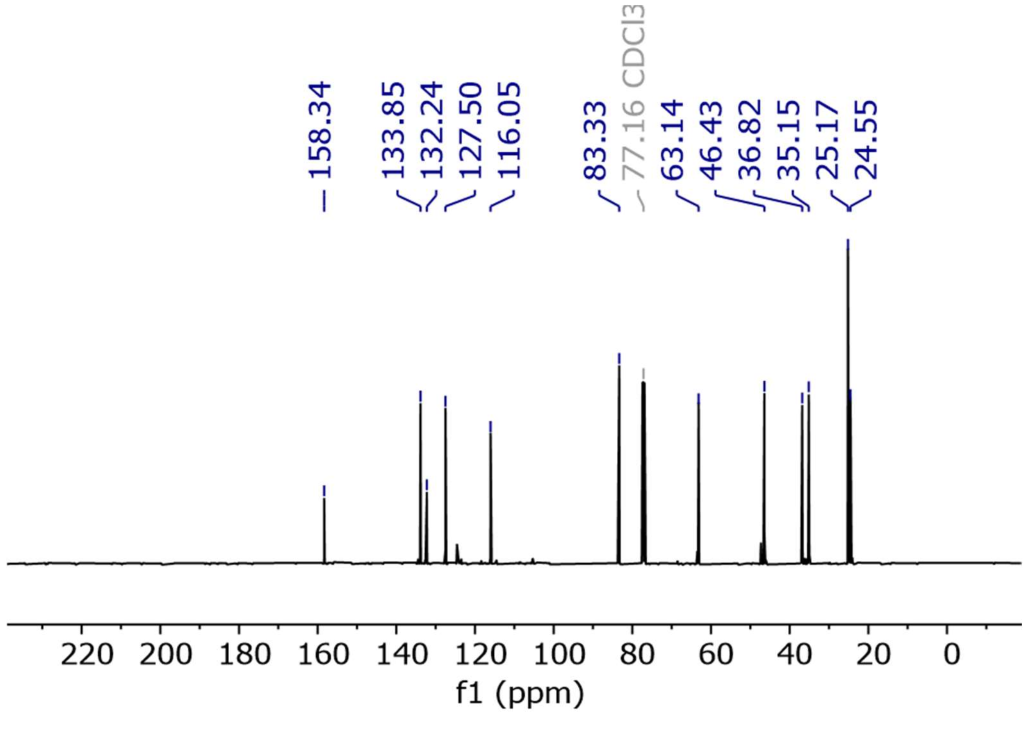

Figure S53: $^{13}C\{^1H\}$ NMR spectrum of 2,3-Tetrahydrocyclopentyl 7-BPin indoline (**4k**) in $CDCl_3$.

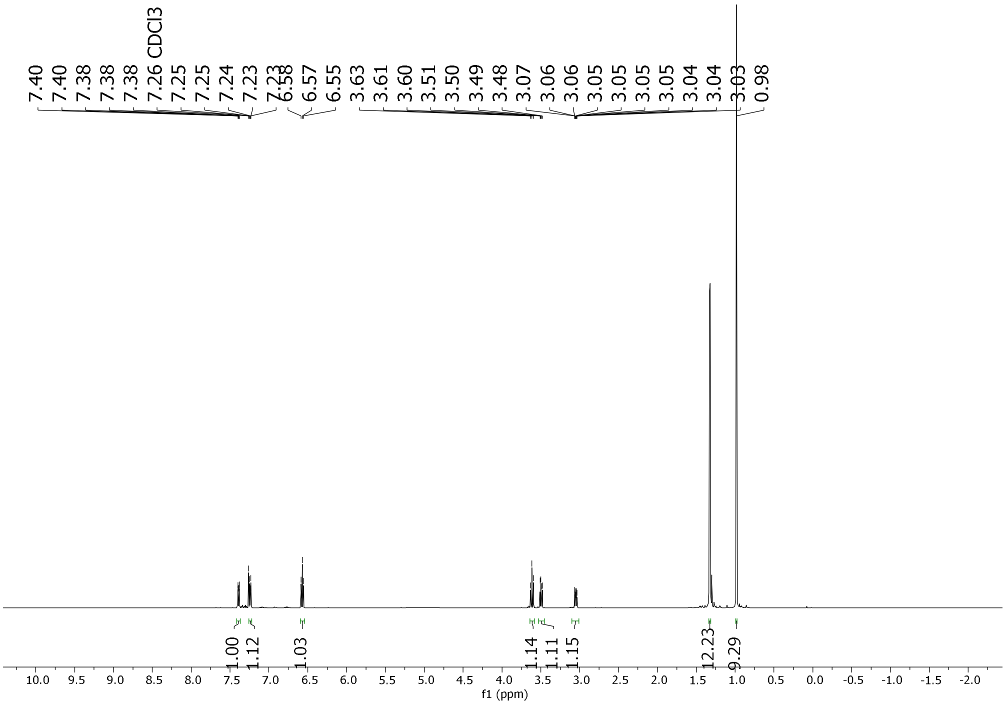

Figure S54. ^{1}H NMR spectrum of 3-tert-butyl-7-Bpin-Indoline (**4l**) in $CDCl_3$.

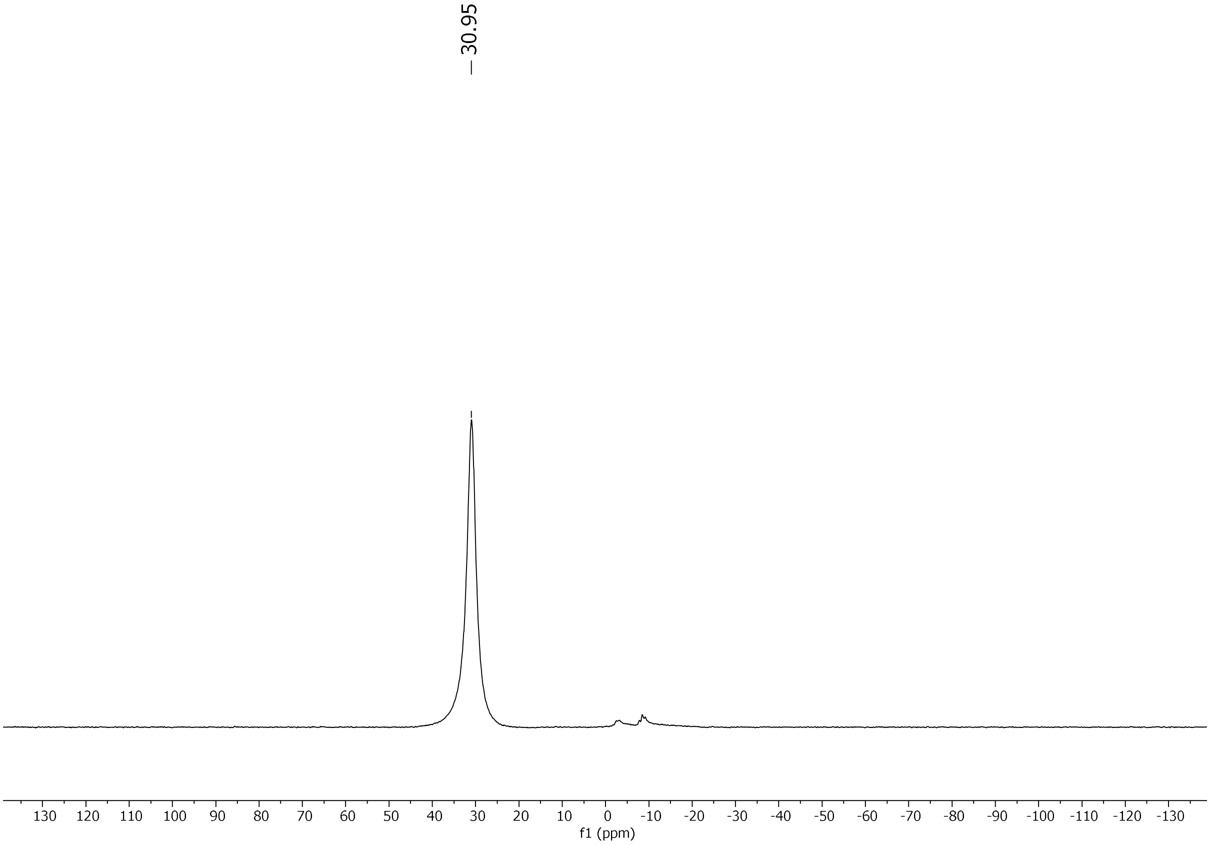

Figure S55. ^{11}B NMR spectrum of 3-tert-butyl-7-Bpin-Indoline (**4l**) in $CDCl_3$.

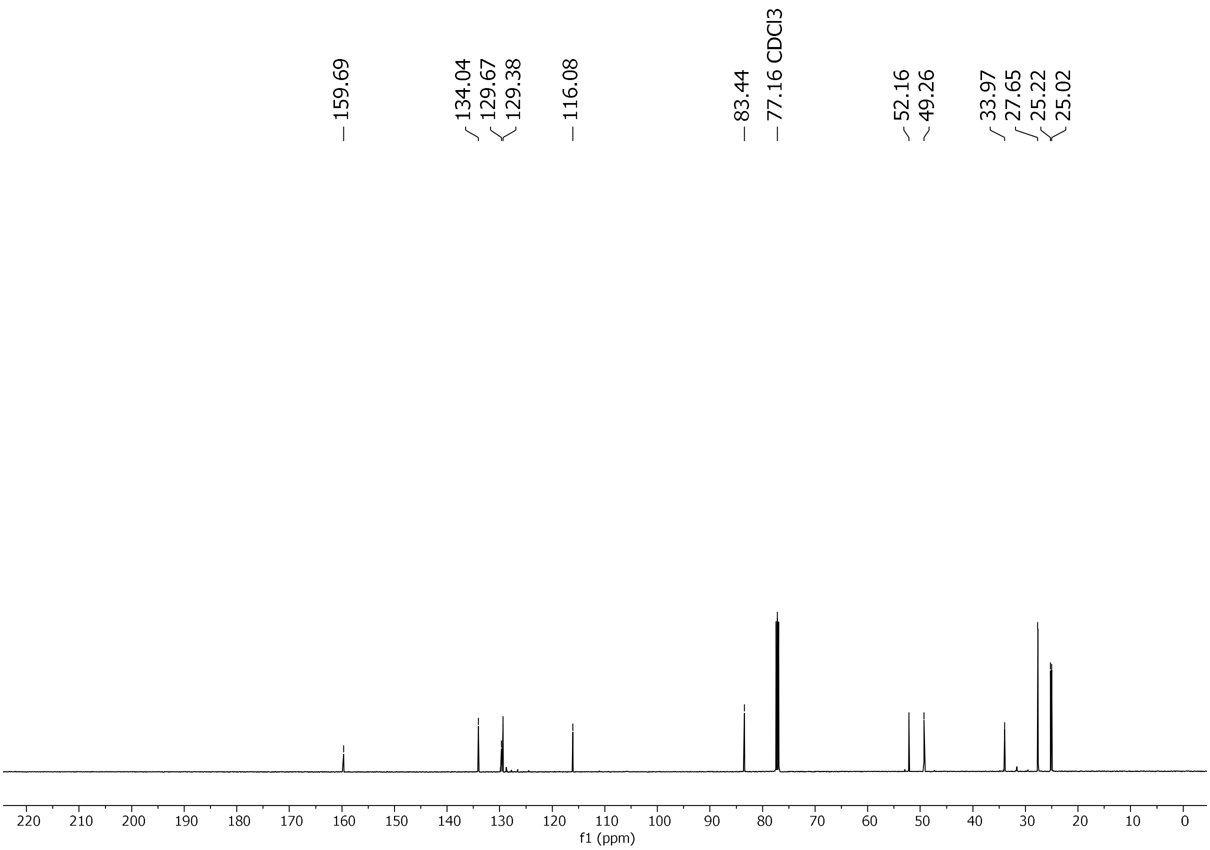

Figure S56. $^{13}C\{^{1}H\}$ NMR spectrum of 3-tert-butyl-7-Bpin-Indoline (**4l**) in $CDCl_3$.

4.0 Mechanistic Studies

4.1 Reaction Profiling with Indole and 5-Cl Indole

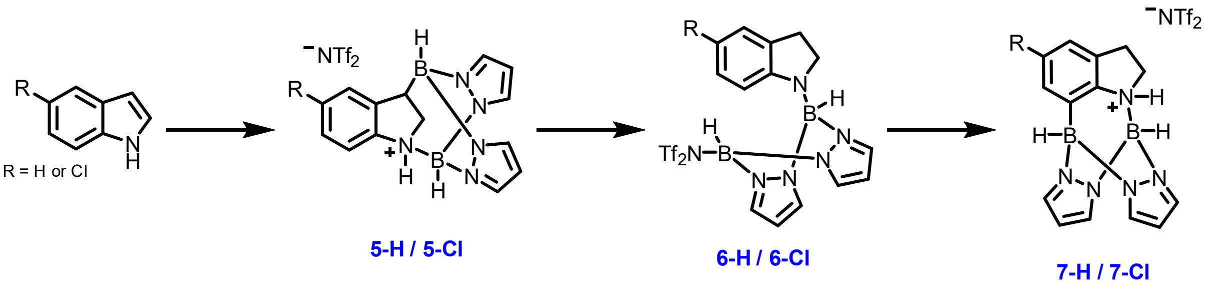

To a J. Young's NMR tube was added indole / 5-chloroindole (0.1 mmol), Pyrazabole **1** (0.55 equiv., 0.055 mmol, 0.009 g), *bis*-NTf_2 Pyrazabole **2** (0.55 equiv., mmol, 0.039 g) and chlorobenzene (0.5 mL). This was mixed by inversion (approx. 1 rotation / s) at room temperature and analysed by NMR at regular time points. Samples were heated without inversion in a heating block.

<u>NMR Profiles of C7 Borylation of Indole with *mono*-NTf_2 Pyrazabole **3**</u>

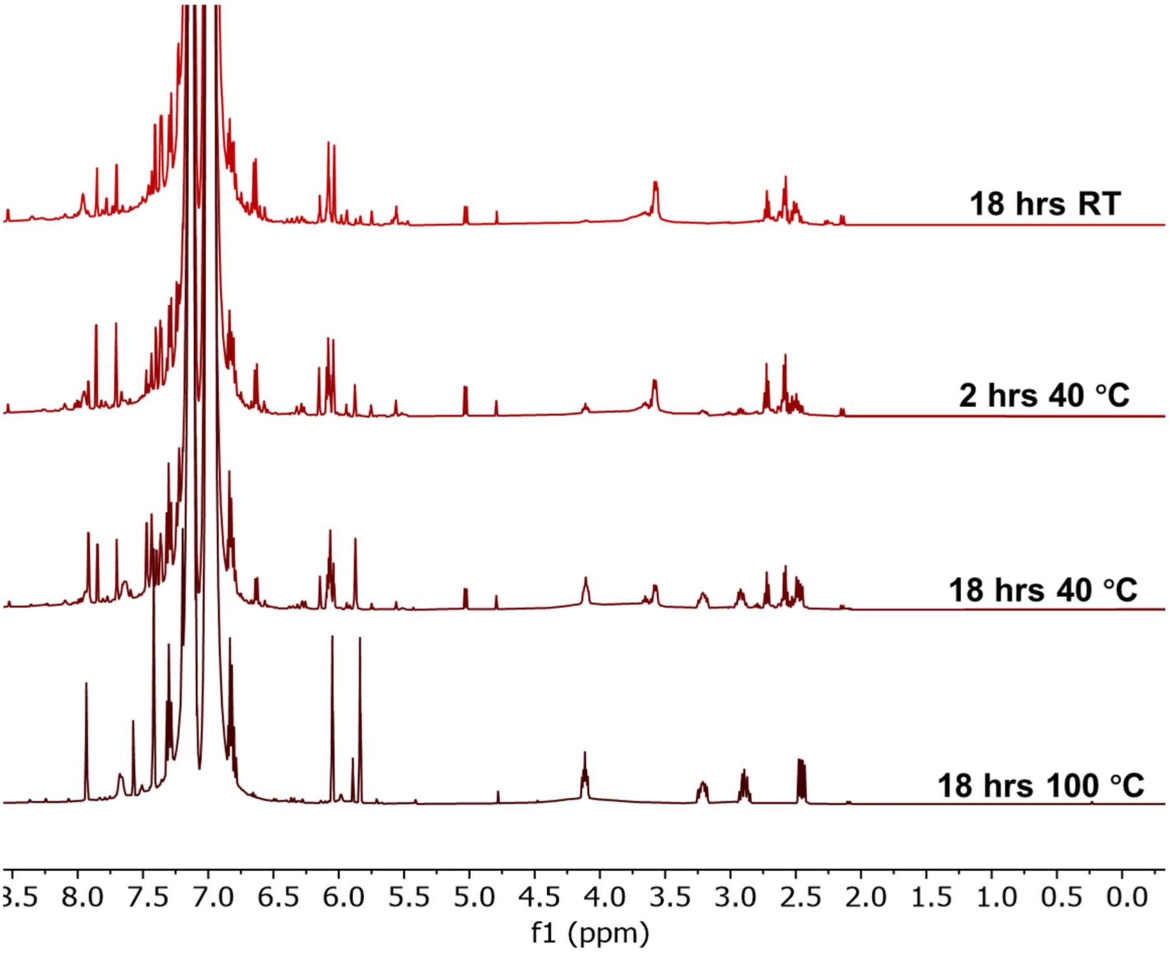

Figure S57: Stacked ^{1}H NMR spectra of the C7-borylation of indole with **3**. Spectra recorded in reaction solvent C_6H_5Cl.

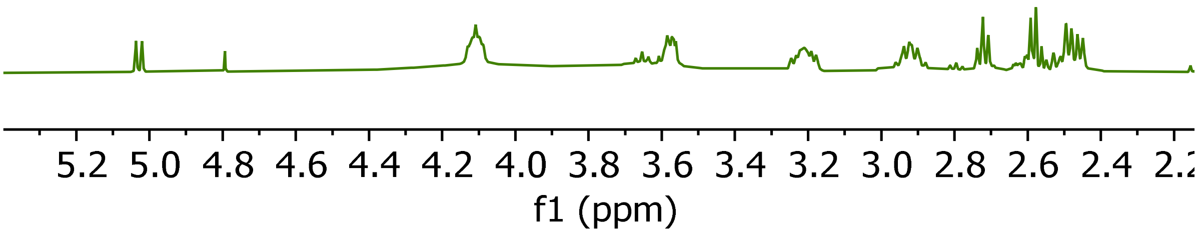

Figure S58: Partial ^{1}H NMR spectra of the C7-borylation of indole with **3** after 18 h at 40°C. Spectra recorded in reaction solvent C_6H_5Cl. This spectrum clearly shows **6-H** (two triplets between 2.5 and 2.7 ppm – vide infra for cleaner spectrum from the reaction of N-H-indoline/**3**/base) and **7-H** are the major species now present.

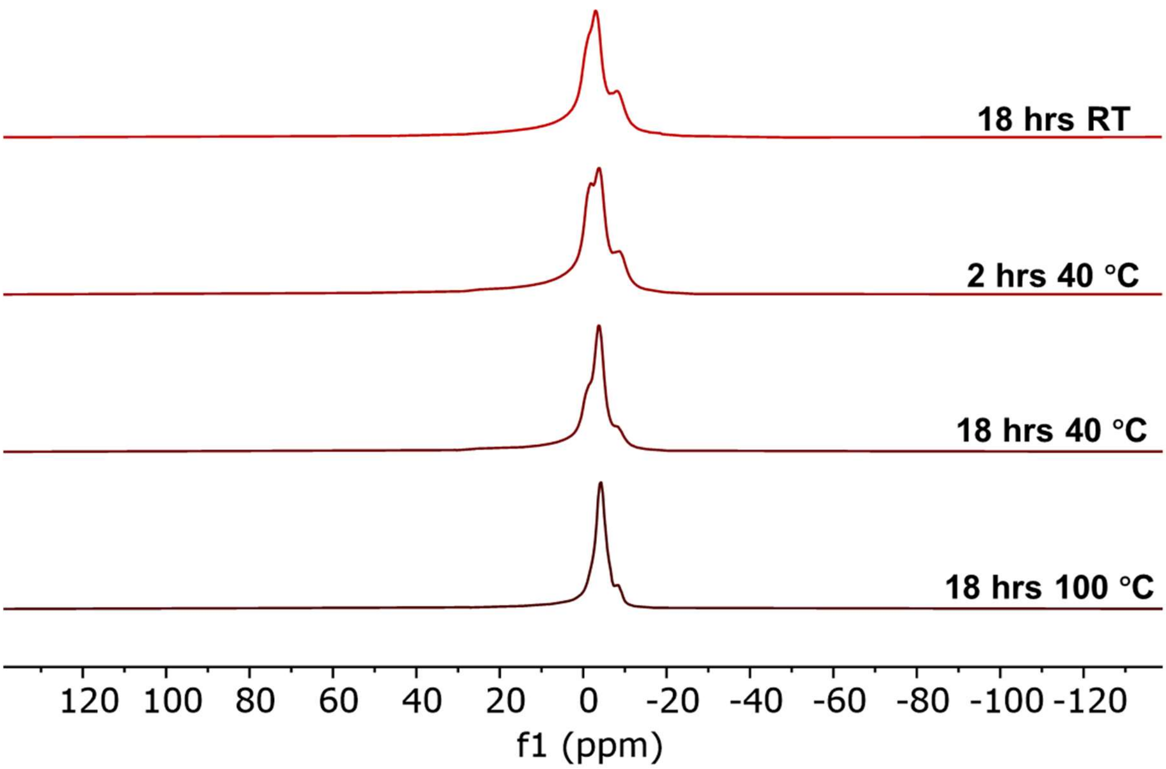

Figure S59: Stacked ^{11}B NMR spectra of the C7-borylation of indole with **3.** Spectra recorded in reaction solvent C_6H_5Cl.

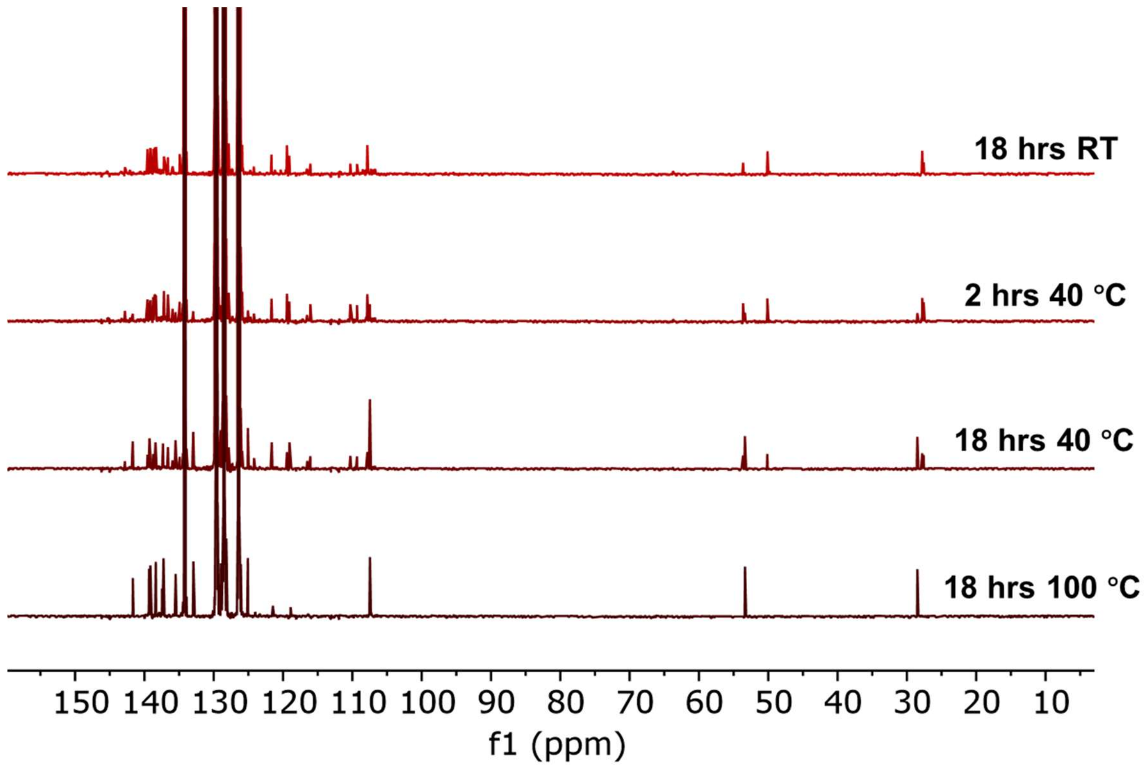

Figure S60: Stacked $^{13}C\{^{1}H\}$ NMR spectra of the C7-borylation of indole with **3**. Spectra recorded in reaction solvent C_6H_5Cl.

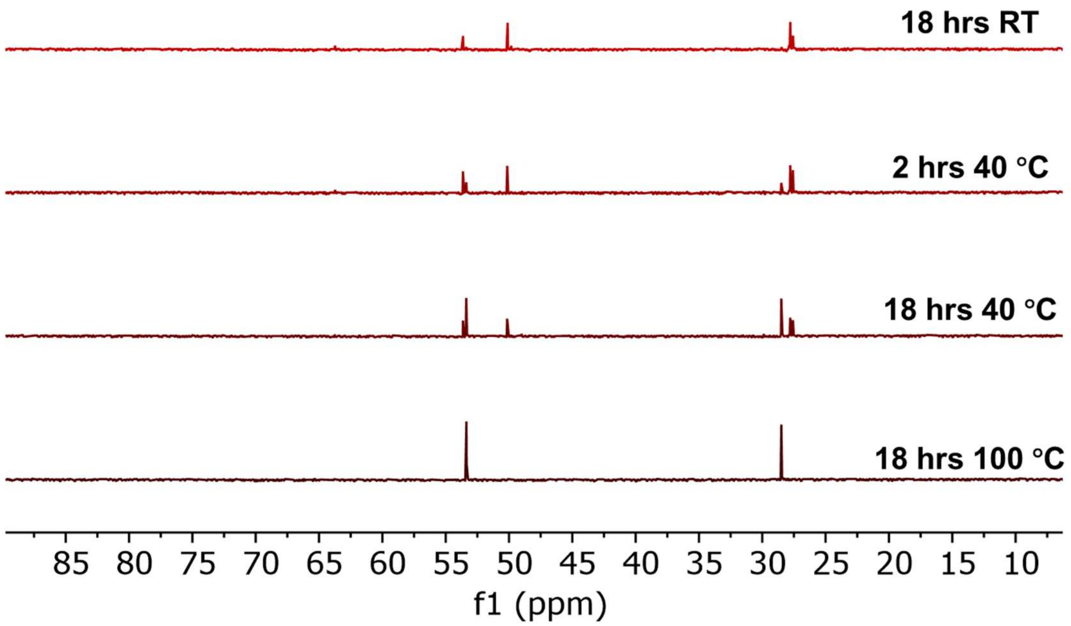

Figure S61: Excerpt (of indoline CH_2 region) of stacked $^{13}C\{^{1}H\}$ NMR spectra of the C7-borylation of indole with **3**. Spectra recorded in reaction solvent C_6H_5Cl.

NMR Profiles of C7 Borylation of 5-Chloroindole with *mono*-NTf_2 Pyrazabole **3**

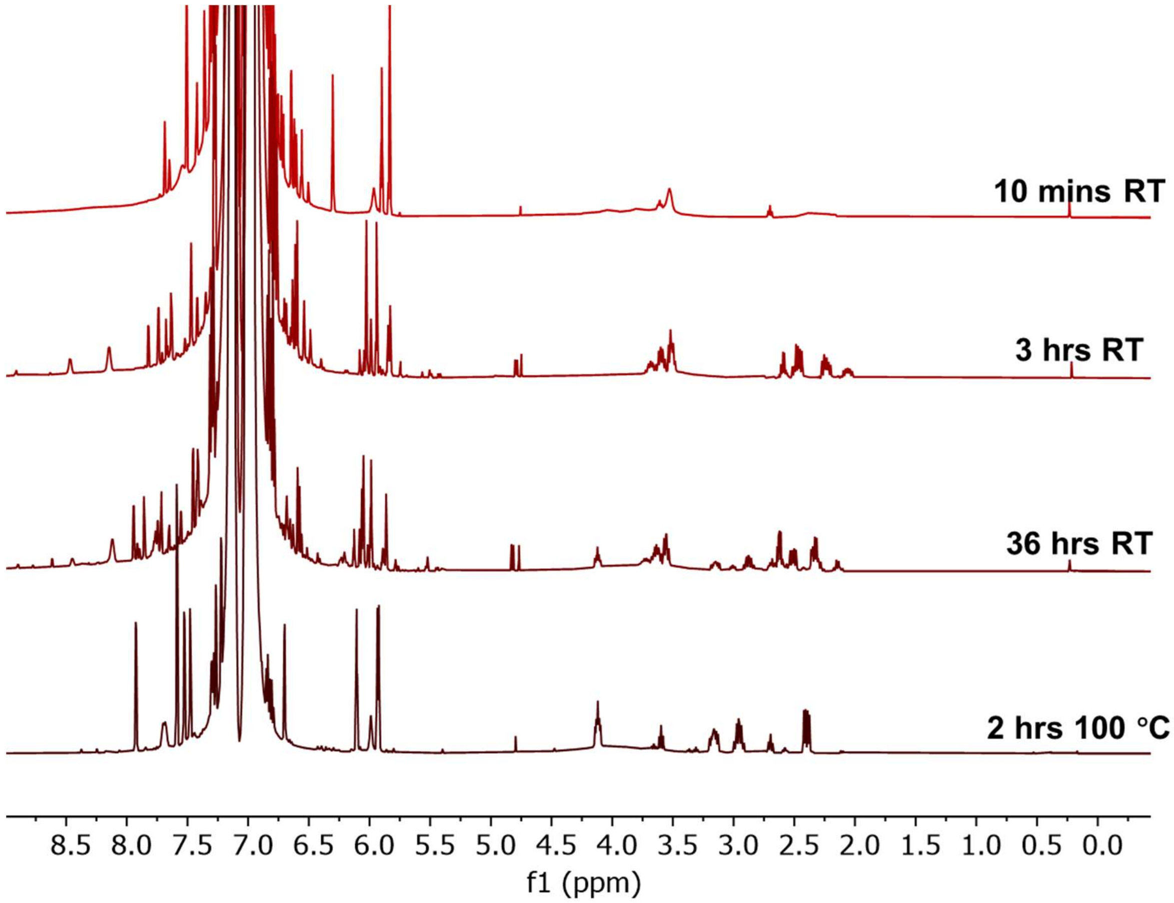

Figure S62: Stacked ^{1}H NMR spectra of the C7-borylation of 5-chloroindole with **3**. Spectra recorded in reaction solvent C_6H_5Cl. *Note: reaction is slower in NMR tubes due to poor solubility of* ***2*** *(and lack of stirring / inversion when heating), hence it is not fully C7-borylated after 2 hours 100 °C (though* ***7-Cl*** *is the major product).*

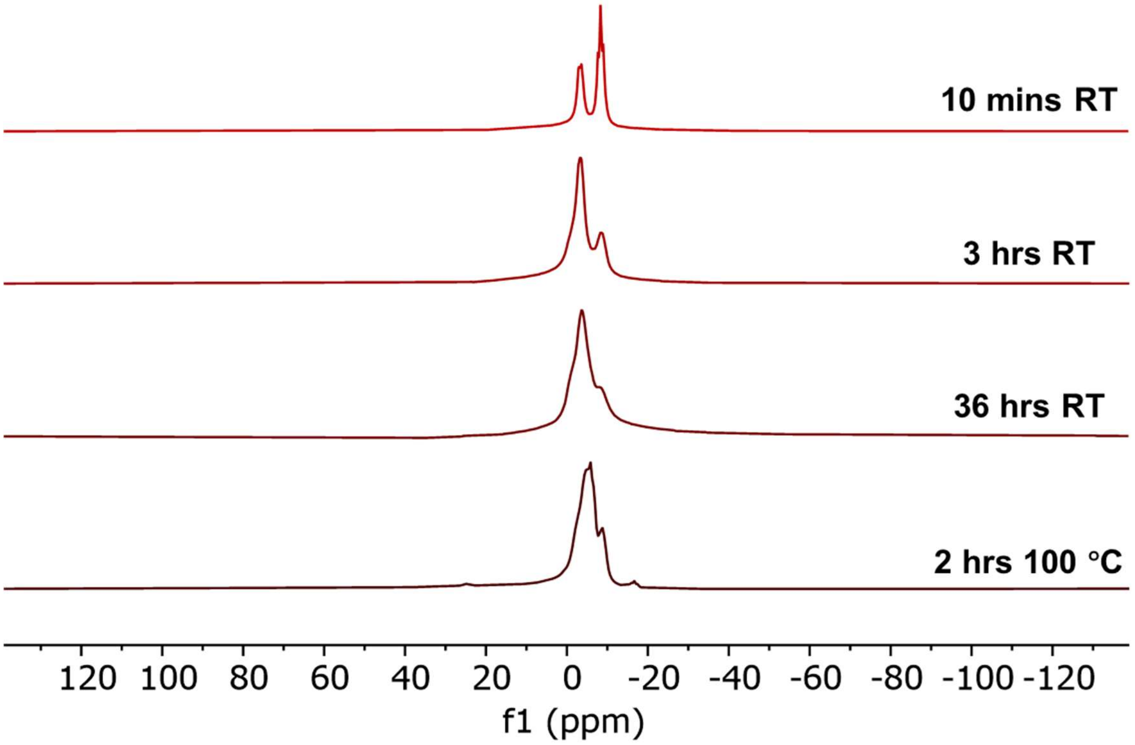

Figure S63: Stacked ^{11}B NMR spectra of the C7-borylation of 5-chloroindole with **3**. Spectra recorded in reaction solvent C_6H_5Cl. *Note: reaction is slower in NMR tubes due to poor solubility of **2** (and lack of mixing), hence not fully C7-borylated after 2 hours 100 °C.*

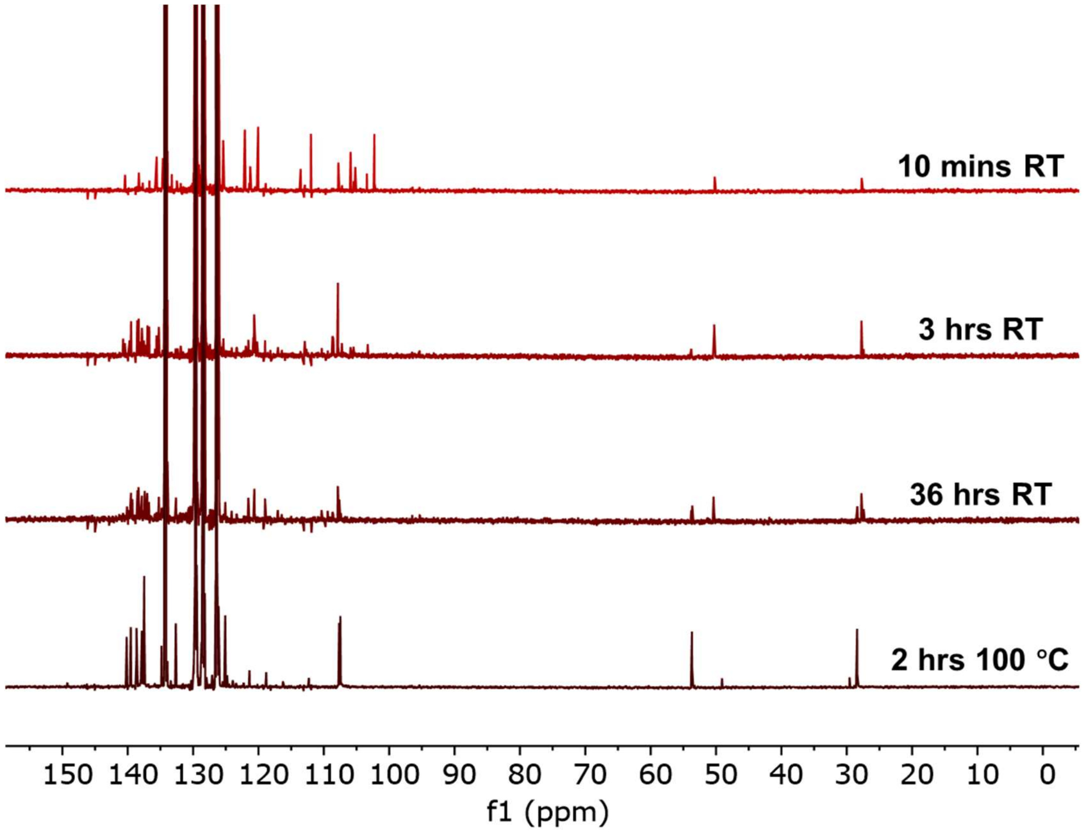

Figure S64: Stacked $^{13}C\{^1H\}$ NMR spectra of the C7-borylation of 5-chloroindole with **3**. Spectra recorded in reaction solvent C_6H_5Cl. *Note: reaction is slower in NMR tubes due to poor solubility of **2**, hence not fully C7-borylated after 2 hours 100 °C.*

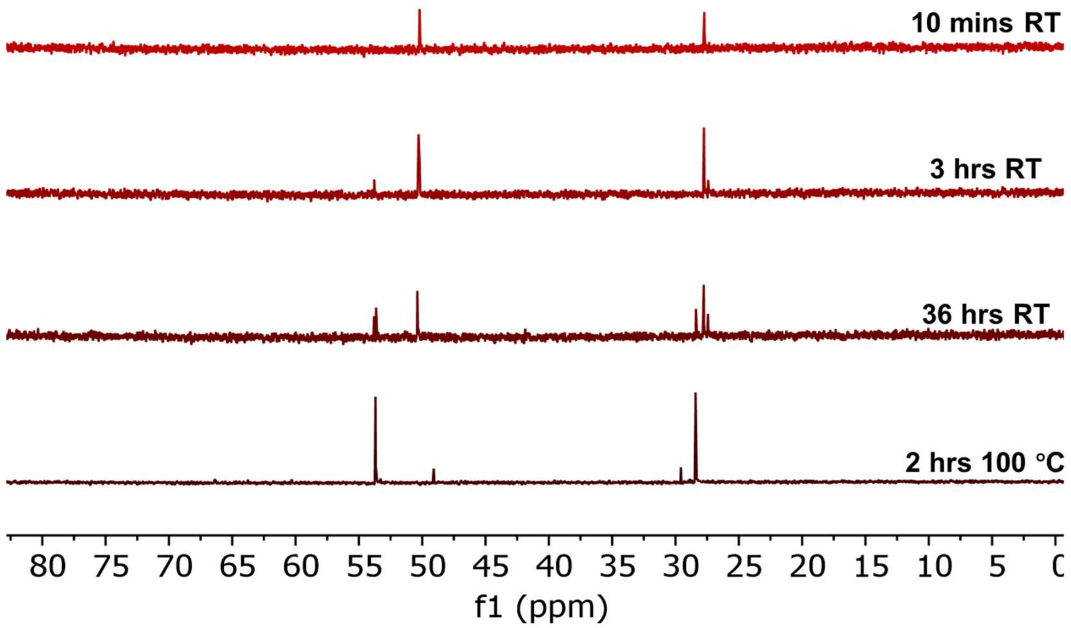

Figure S65: Excerpt of stacked $^{13}C\{^1H\}$ NMR spectra of the C7-borylation of 5-chloroindole with **3**. Spectra recorded in reaction solvent C_6H_5Cl. *Note: reaction is slower in NMR tubes due to poor solubility of **2**, hence not fully C7-borylated after 2 hours 100 °C.*

4.2 Characterisation of C7-Borylated Intermediates 7-H / 7-Cl

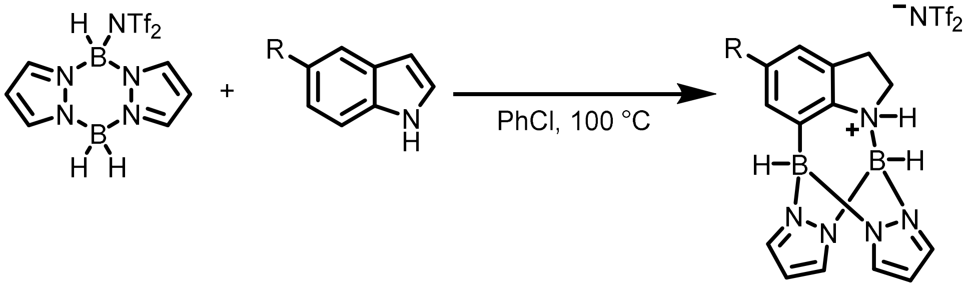

R = H: 7-H
R = Cl: 7-Cl

Compound 7-H

An ampule was charged with pyrazabole **1** (0.018 g, 0.11 mmol, 0.55 equiv.), *bis*-NTf_2 pyrazabole **2** (0.079 g, 0.11 mmol, 0.55 equiv.) and indole (0.023 g, 0.1 mmol, 1 equiv.), then suspended in chlorobenzene (1.5 mL). The reaction was heated to 100 °C in a sealed ampule for 18 hours. The reaction was then concentrated, and the solvent replaced with $CDCl_3$ for NMR characterisation.

^{1}H NMR (500 MHz, $CDCl_3$) δ 8.14 (d, $^3J_{HH}$ = 3 Hz, 1 H, N=C*H*-CH), 7.95 (d, $^3J_{HH}$ = 3 Hz, 1 H, N=C*H*-CH), 7.92 (d, $^3J_{HH}$ = 3 Hz, 1 H, N=C*H*-CH), 7.77 (d, $^3J_{HH}$ = 3 Hz, 1 H, N=C*H*-CH), 7.7 (br, s, 1H N-H), 7.46 (d, $^3J_{HH}$ = 7 Hz, 1 H, ArC*H*), 7.31 (t, $^3J_{HH}$ = 7 Hz, 1 H, ArC*H*), 7.22 (d, $^3J_{HH}$ = 7 Hz, 1 H, ArC*H*), 6.53 (dd, $^3J_{HH}$ = 3 Hz, 3 Hz, 1 H, N=CH-C*H*), 6.45 (dd, $^3J_{HH}$ = 3 Hz, 3 Hz, 1 H, N=CH-C*H*), 4.29 (m, 1H, N-CH_2-CH_2), 3.38 (m, 1H, N-CH_2-CH_2), 3.25 (m, 1H, N-CH_2-CH_2), 2.89 (m, 1H, N-CH_2-CH_2);

^{1}H{^{11}B} NMR (500 MHz, $CDCl_3$) δ 8.14 (d, $^3J_{HH}$ = 3 Hz, 1 H, N=C*H*-CH), 7.95 (d, $^3J_{HH}$ = 3 Hz, 1 H, N=C*H*-CH), 7.92 (d, $^3J_{HH}$ = 3 Hz, 1 H, N=C*H*-CH), 7.77 (d, $^3J_{HH}$ = 3 Hz, 1 H, N=C*H*-CH), 7.7 (br, s, 1H N-H), 7.46 (d, $^3J_{HH}$ = 7 Hz, 1 H, ArC*H*), 7.31 (t, $^3J_{HH}$ = 7 Hz, 1 H, ArC*H*), 7.22 (d, $^3J_{HH}$ = 7 Hz, 1 H, ArC*H*), 6.53 (dd, $^3J_{HH}$ = 3 Hz, 3 Hz, 1 H, N=CH-C*H*), 6.45 (dd, $^3J_{HH}$ = 3 Hz, 3 Hz, 1 H, N=CH-C*H*), 4.35 (br, 2H, B*H*), 4.29 (m, 1H, N-CH_2-CH_2), 3.38 (m, 1H, N-CH_2-CH_2), 3.25 (m, 1H, N-CH_2-CH_2), 2.89 (m, 1H, N-CH_2-CH_2);

^{11}B NMR (160 MHz, $CDCl_3$) δ -4.0 (br, 7-,N-B(Pyrz));

^{11}B{^{1}H} NMR (160 MHz, $CDCl_3$) δ -3.6 (s, 7-,N-B(Pyrz)), -5.2 (s, 7-,N-B(Pyrz));

^{13}C{^{1}H} NMR (126 MHz, $CDCl_3$) δ 141.93, 139.76, 139.67, 138.86, 137.97, 135.77, 133.20, 129.30, 125.37 119.92 (q, $^1J_{CF}$ = 321 Hz, S*C*F_3), 108.07, 107.98, 53.67, 28.79, (*C-B was not visible even via ^{1}H ^{13}C HMBC);*

^{19}F NMR (471 MHz, $CDCl_3$) δ -78.61 (s) ppm.

HRMS (ESI$^+$) *m/z* calcd for $C_{14}H_{16}B_2N_5^+$: 276.1586 [M]$^+$, found 276.1596.

Compound 7-Cl

An ampule was charged with pyrazabole **1** (0.018 g, 0.11 mmol, 0.55 equiv.), *bis*-NTf_2 pyrazabole **2** (0.079 g, 0.11 mmol, 0.55 equiv.) and 5-chloroindole (0.030 g, 0.1 mmol, 1 equiv.), then suspended in chlorobenzene (1.5 mL). The reaction was heated to 100 °C in a sealed ampule for 2 hours. The reaction was then concentrated, and the solvent replaced with $CDCl_3$ for NMR characterisation. Note the broad N-H resonance observed for 7-H is not observed in the spectra of this congener, all other data are closely comparable between 7-H and 7-Cl supporting our formulation as 7-Cl.

^{1}H NMR (500 MHz, $CDCl_3$) δ 8.12 (d, $^3J_{HH}$ = 2.5 Hz, 1H, N=C*H*-CH), 7.94 (d, $^3J_{HH}$ = 2.5 Hz, 1H, N=C*H*-CH), 7.92 (d, $^3J_{HH}$ = 2.5 Hz, 1H, N=C*H*-CH), 7.77 (d, $^3J_{HH}$ = 2.5 Hz, 1H, N=C*H*-CH), 7.41 (d, $^4J_{HH}$ = 1.5 Hz, 1H, ArC*H*), 7.17 (d, $^4J_{HH}$ = 1.5 Hz, 1H, ArC*H*), 6.55 (dd, $^3J_{HH}$ = 2.5 Hz, 2.5 Hz, 1H, N=CH-C*H*), 6.46 (dd, $^3J_{HH}$ = 2.5 Hz, 2.5 Hz, 1H, N=CH-C*H*), 4.29 (m , 1H, N-CH_2-CH_2), 3.36 (m , 1H, N-CH_2-CH_2), 3.26 (m , 1H, N-CH_2-CH_2), 2.88 (m , 1H, N-CH_2-CH_2);

^{1}H{^{11}B} NMR (500 MHz, $CDCl_3$) δ 8.12 (d, $^3J_{HH}$ = 2.5 Hz, 1H, N=C*H*-CH), 7.94 (d, $^3J_{HH}$ = 2.5 Hz, 1H, N=C*H*-CH), 7.92 (d, $^3J_{HH}$ = 2.5 Hz, 1H, N=C*H*-CH), 7.77 (d, $^3J_{HH}$ = 2.5 Hz, 1H, N=C*H*-CH), 7.41 (d, $^4J_{HH}$ = 1.5 Hz, 1H, ArC*H*), 7.17 (d, $^4J_{HH}$ = 1.5 Hz, 1H, ArC*H*), 6.55 (dd, $^3J_{HH}$ = 2.5 Hz, 2.5 Hz, 1H, N=CH-C*H*), 6.46 (dd, $^3J_{HH}$ = 2.5 Hz, 2.5 Hz, 1H, N=CH-C*H*), 4.31 (br, 2H, B*H*), 4.29 (m , 1H, N-CH_2-CH_2), 3.36 (m , 1H, N-CH_2-CH_2), 3.26 (m , 1H, N-CH_2-CH_2), 2.88 (m , 1H, N-CH_2-CH_2);

^{11}B NMR (160 MHz, $CDCl_3$) δ -4.1 (br, 7-,N-*B*(Pyrz));

^{11}B{^{1}H} NMR (160 MHz, $CDCl_3$) δ -3.8 (s, 7-,N-B(Pyrz)), -5.2 (s, 7-,N-B(Pyrz));

^{13}C{^{1}H} NMR (126 MHz, $CDCl_3$) δ 140.43, 139.96, 139.92, 139.11, 138.20, 138.02, 135.15, 133.00, 125.38, 119.84 (q, $^1J_{CF}$ = 321 Hz, S*C*F_3), 108.28, 108.20, 53.94, 28.72.

^{19}F NMR (471 MHz, $CDCl_3$) δ -78.70 (s) ppm.

HRMS (ESI$^+$) *m/z* calcd for $C_{14}H_{15}B_2N_5Cl^+$: 310.1197 [M]$^+$, found 310.1198.

NMR Spectra of Compound **7-H**:

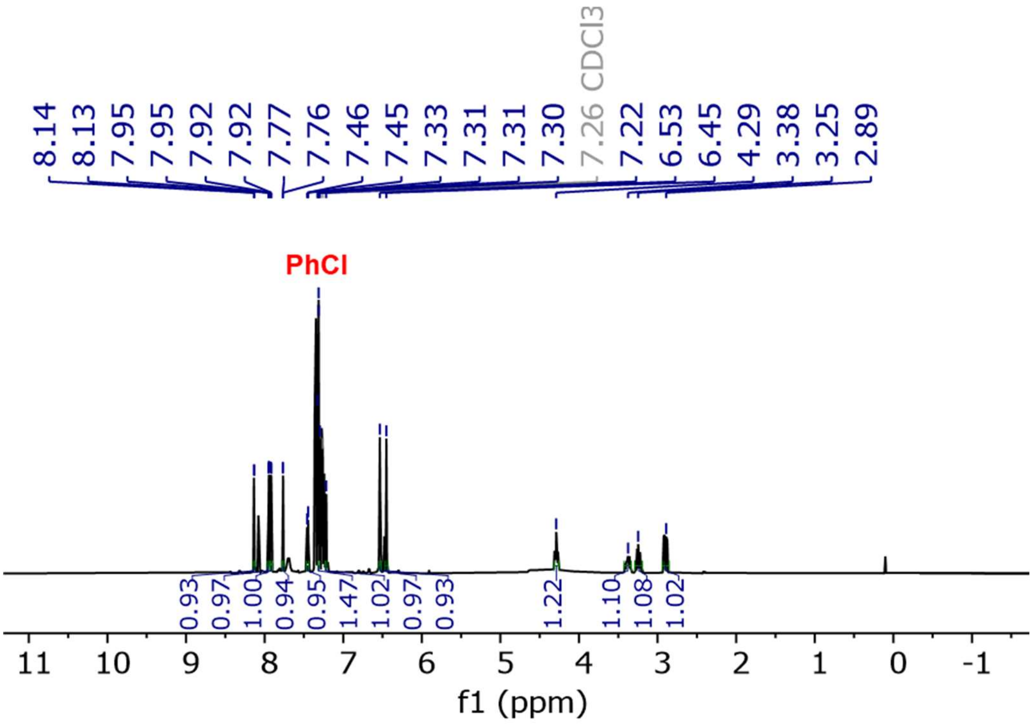

Figure S66: ^{1}H NMR spectrum of C7-borylated intermediate **7-H** in $CDCl_3$. *Note: some C_6H_5Cl remains despite a prolonged period drying under vacuum.*

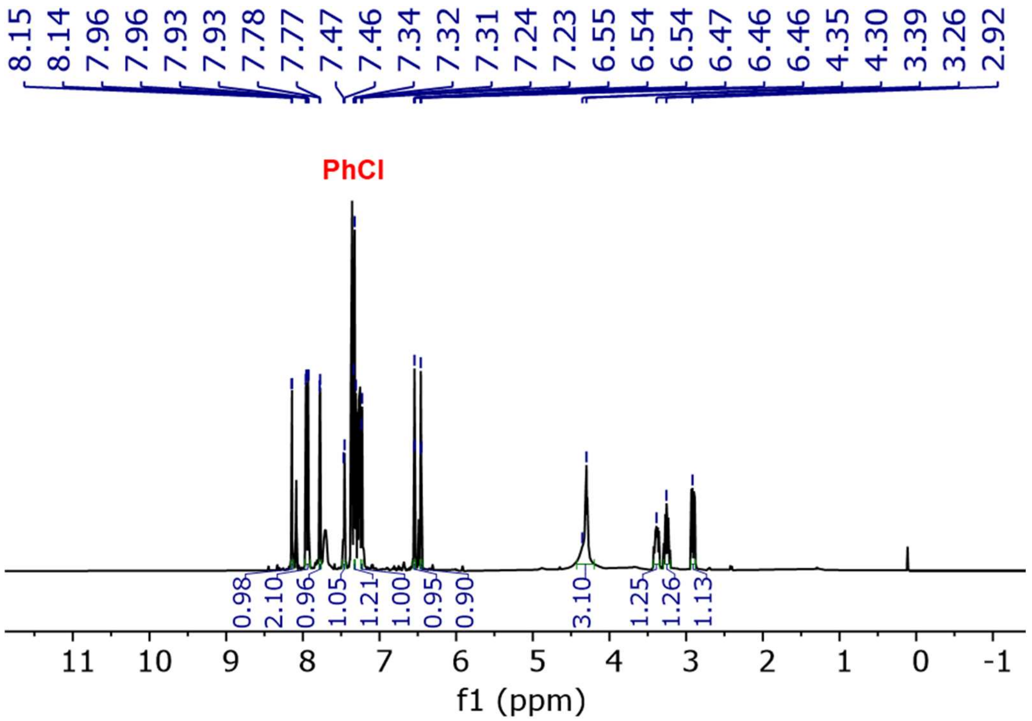

Figure S67: $^{1}H\{^{11}B\}$ NMR spectrum of C7-borylated intermediate **7-H** in $CDCl_3$. *Note: some C_6H_5Cl remains despite a prolonged period drying under vacuum.*

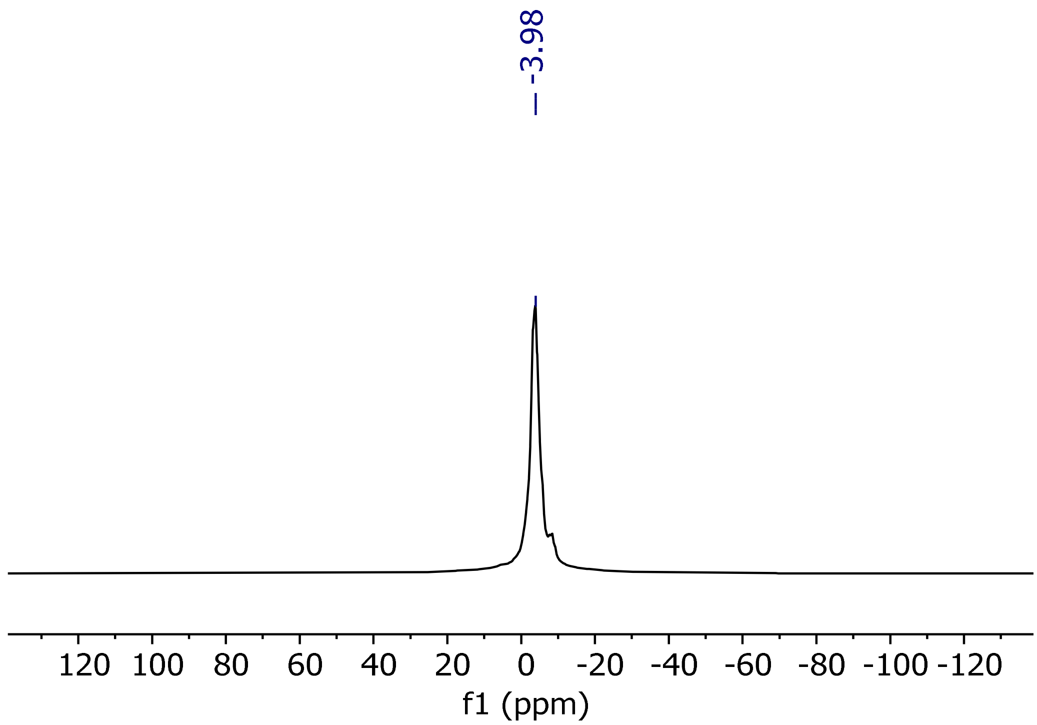

Figure S68: ^{11}B NMR spectrum of C7-borylated intermediate **7-H** in $CDCl_3$.

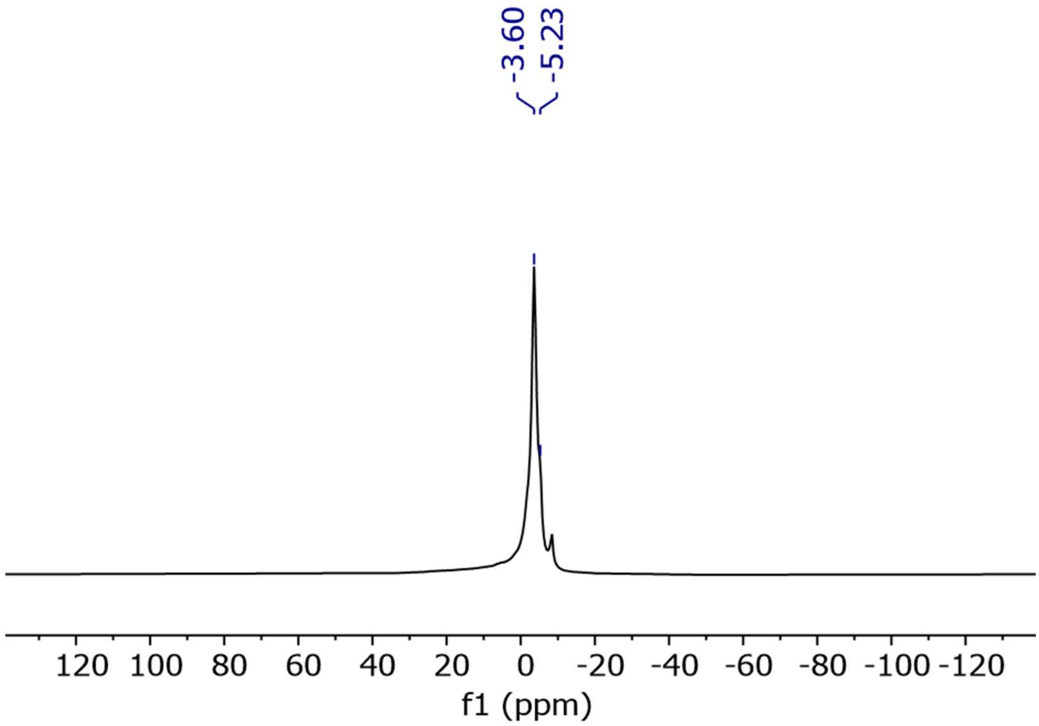

Figure S69: $^{11}B\{^1H\}$ NMR spectrum of C7-borylated intermediate **7-H** in $CDCl_3$.

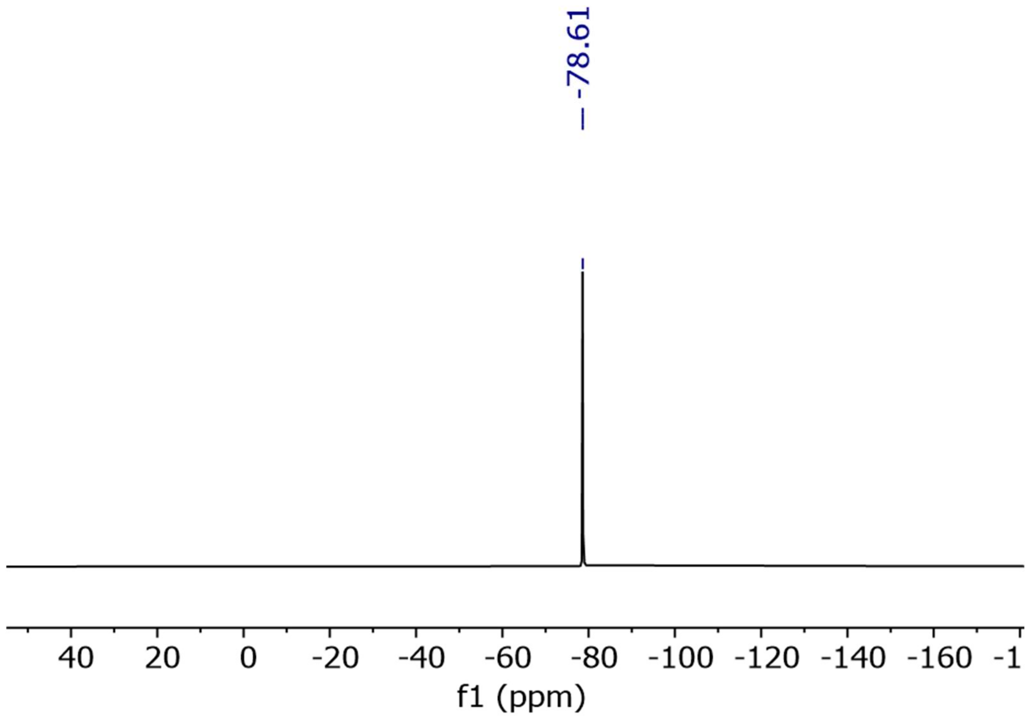

Figure S70: ^{19}F NMR spectrum of C7-borylated intermediate **7-H** in $CDCl_3$.

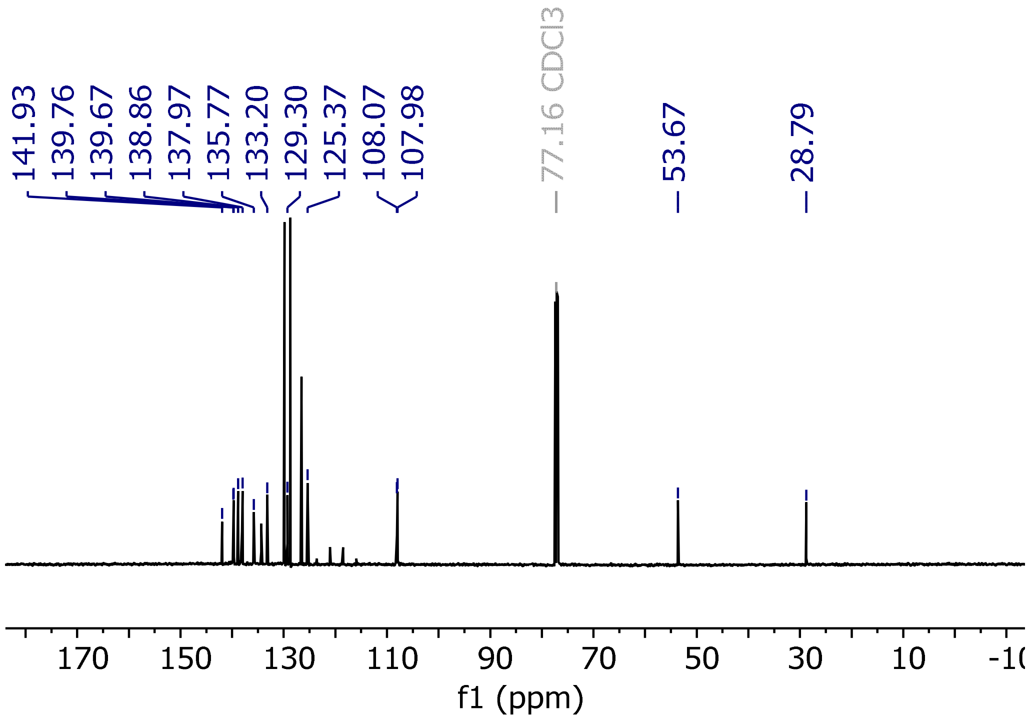

Figure S71: $^{13}C\{^{1}H\}$ NMR spectrum of C7-borylated intermediate **7-H** in $CDCl_3$. *Note: some C_6H_5Cl remains despite a prolonged period drying under vacuum.*

NMR Spectra of [5-Cl 7-,N-B(Pyrazabole) Indoline][NTf_2] **7-Cl**:

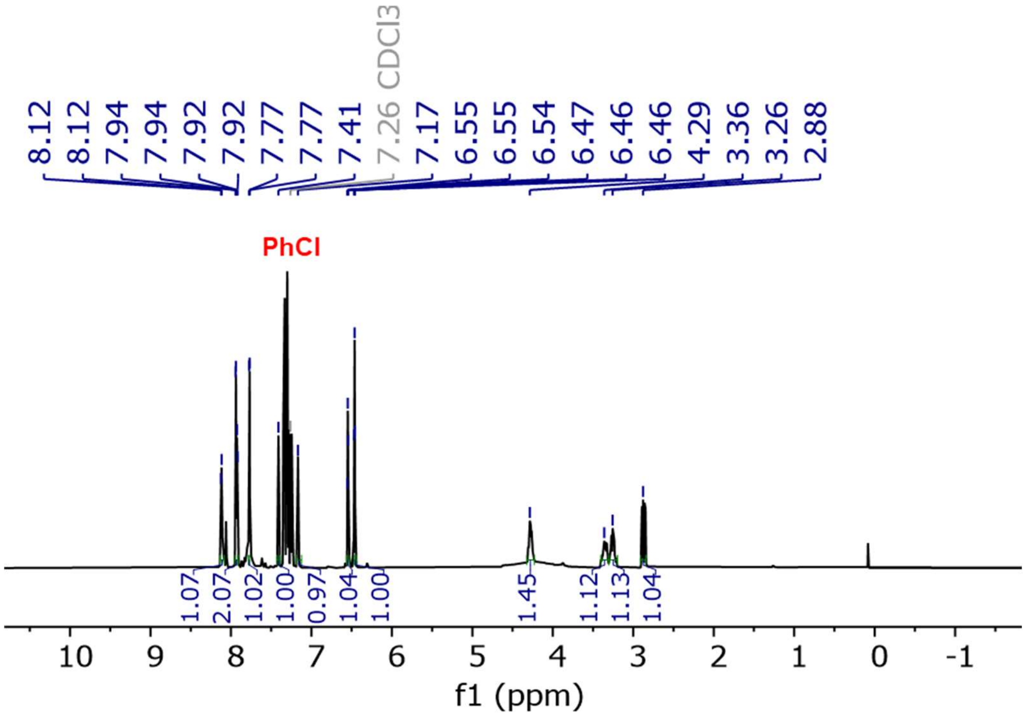

Figure S72: ^{1}H NMR spectrum of C7-borylated intermediate **7-Cl** in $CDCl_3$. *Note: some C_6H_5Cl remains despite a prolonged period drying under vacuum.*

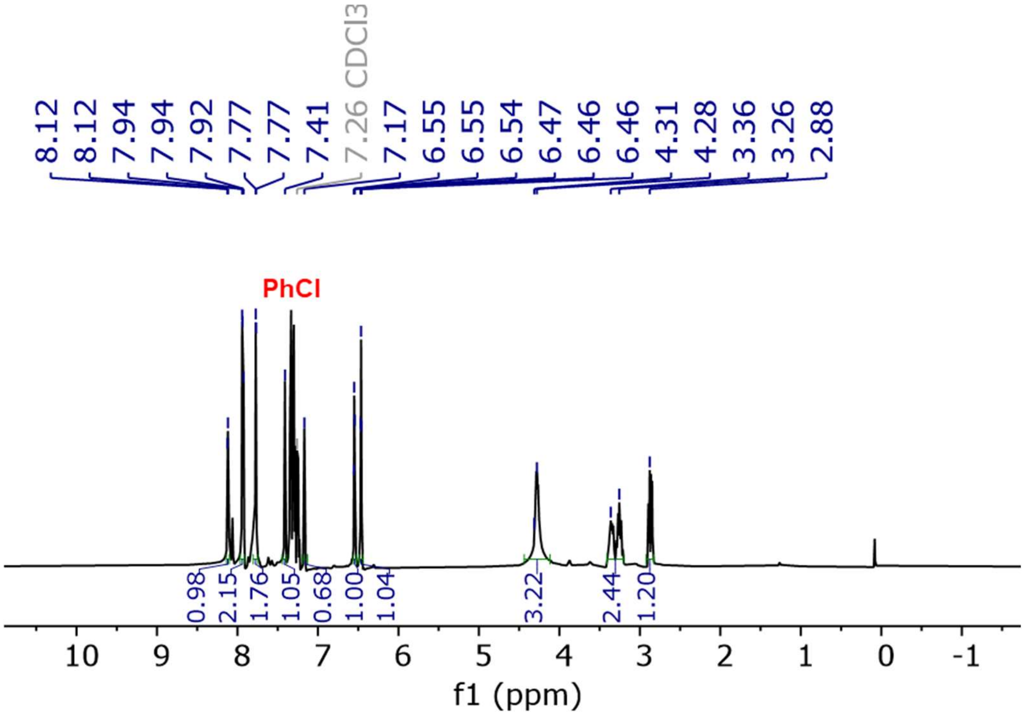

Figure S73: $^{1}H\{^{11}B\}$ NMR spectrum of C7-borylated intermediate **7-Cl** in $CDCl_3$. *Note: some C_6H_5Cl remains despite a prolonged period drying under vacuum.*

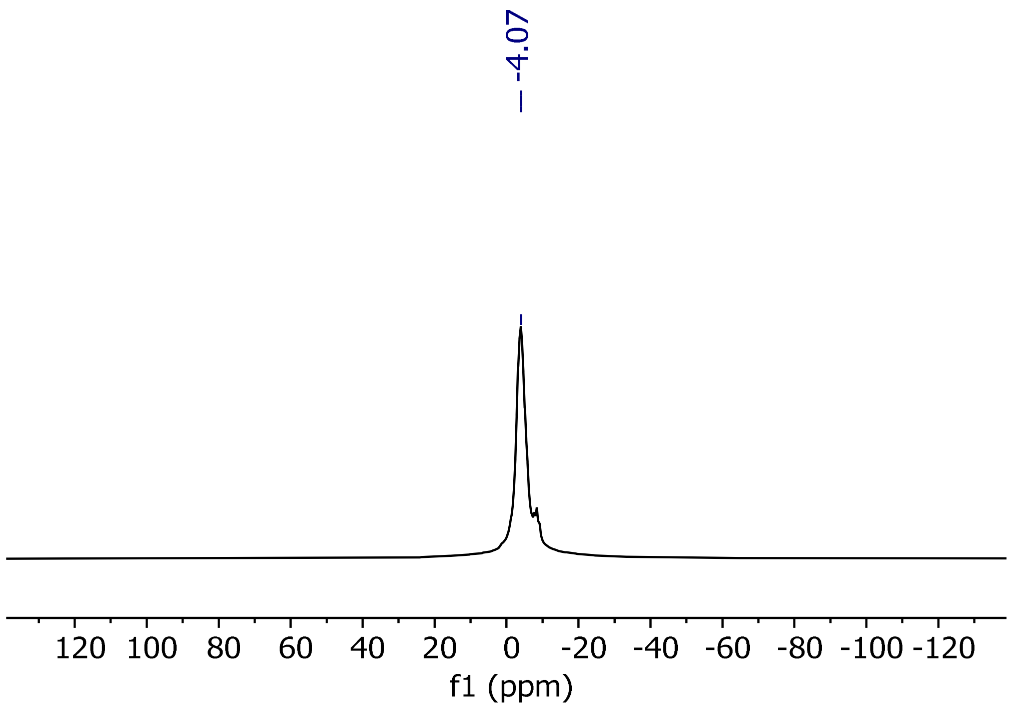

Figure S74: ^{11}B NMR spectrum of C7-borylated intermediate **7-Cl** in $CDCl_3$.

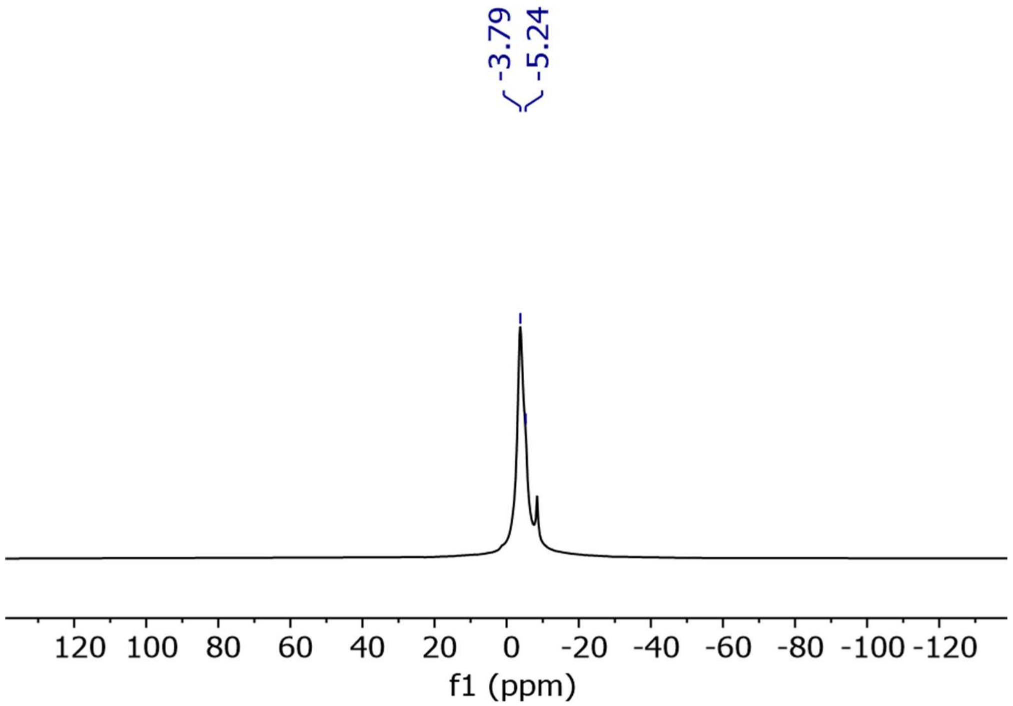

Figure S75: $^{11}B\{^{1}H\}$ NMR spectrum of C7-borylated intermediate **7-Cl** in $CDCl_3$.

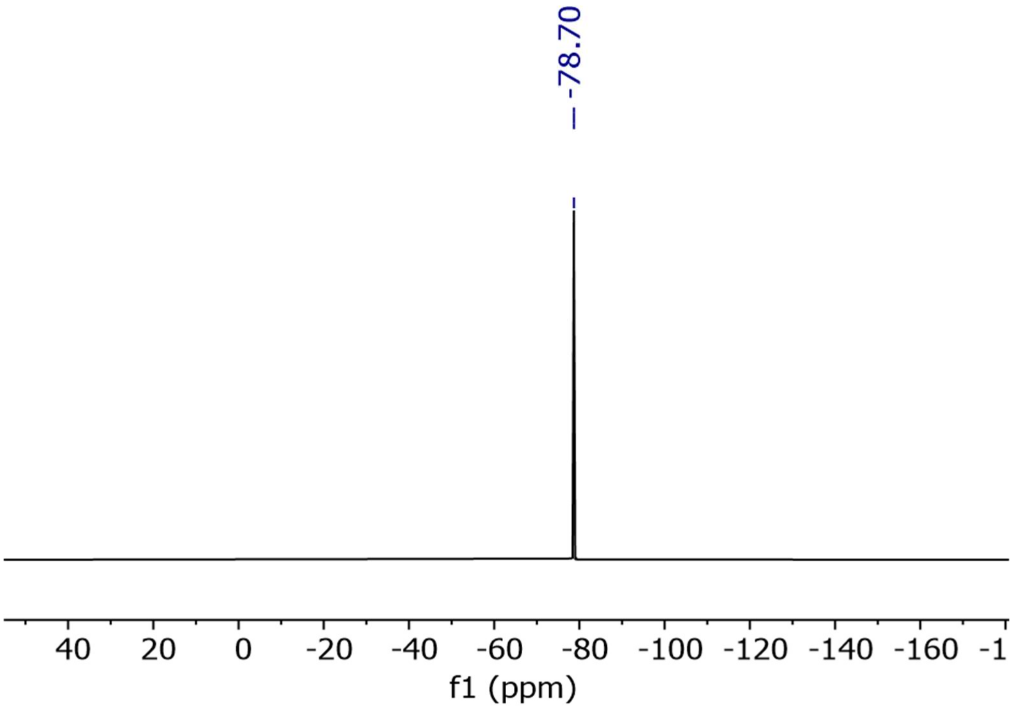

Figure S76: ^{19}F NMR spectrum of C7-borylated intermediate **7-Cl** in $CDCl_3$.

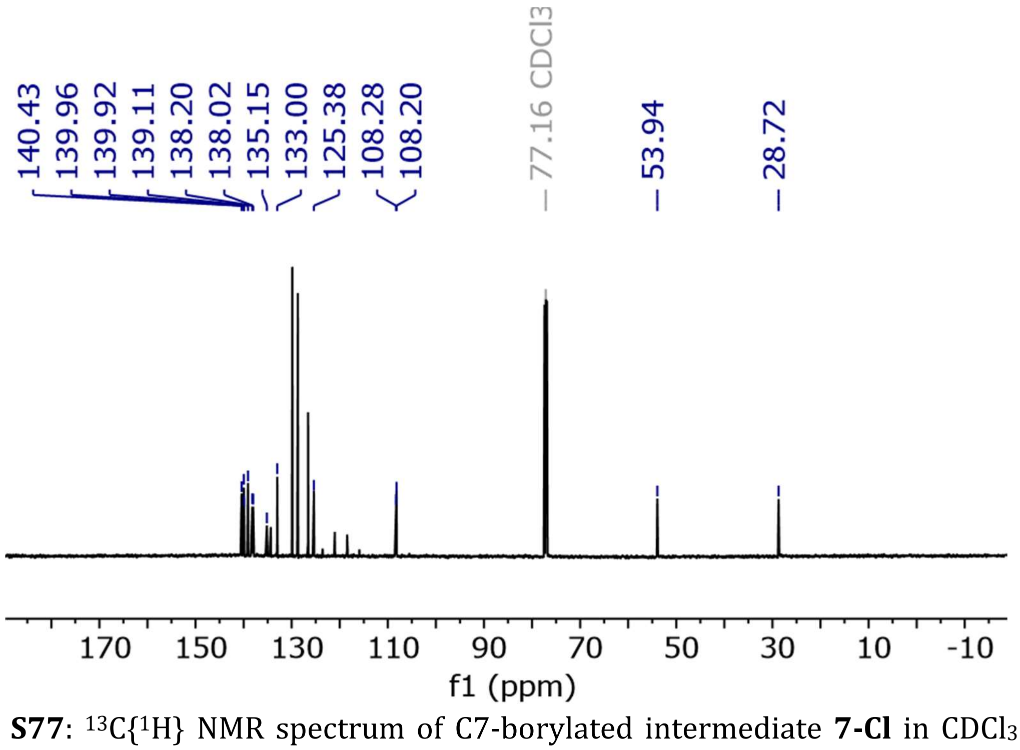

Figure S77: $^{13}C\{^{1}H\}$ NMR spectrum of C7-borylated intermediate **7-Cl** in $CDCl_3$. *Note: some C_6H_5Cl remains despite a prolonged period drying under vacuum.*

4.3 Addition of Exogenous Base to C7-Borylayed Intermediate 7-H

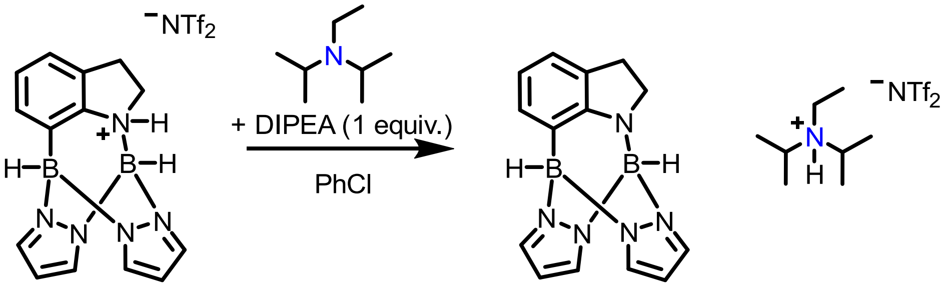

To a sample of **7-H** (0.1 mmol, 0.056 g) in PhCl (0.5 mL) was added N,N-Diisopropylethylamine (1 equiv., 0.017 mL). NMR recorded.

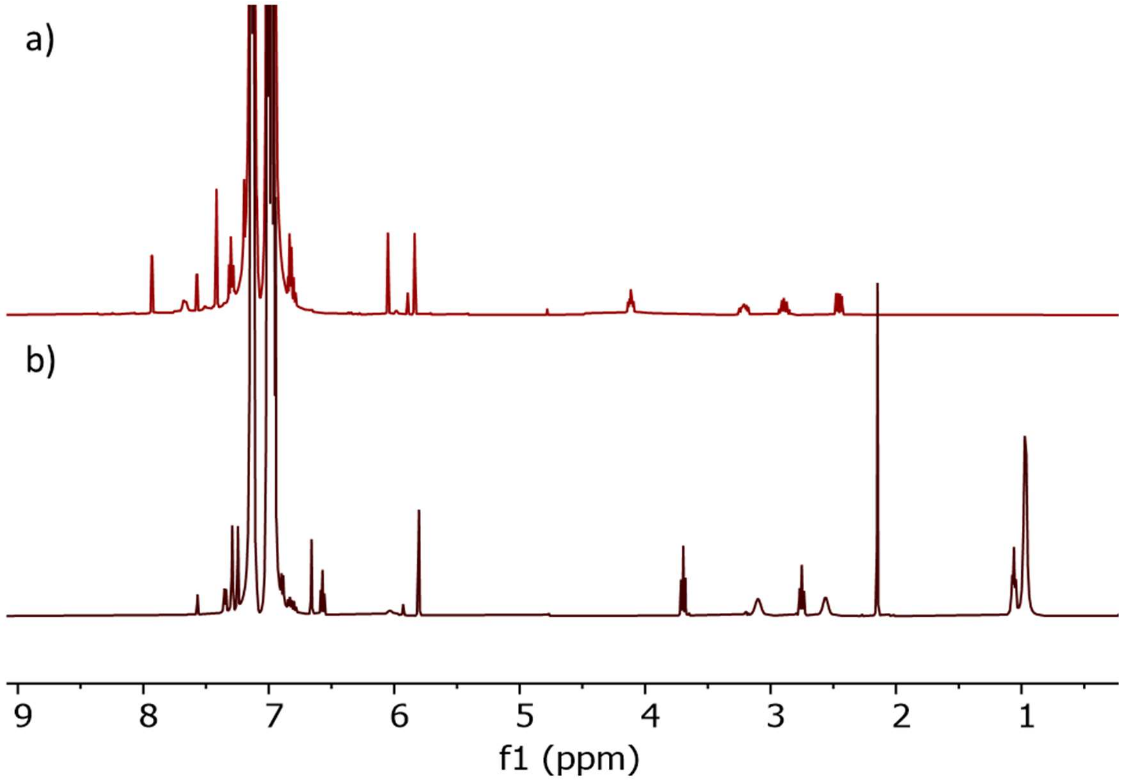

Figure S78: ^{1}H NMR spectra of C7-borylated intermediate **7-H** before (top, a) and after (bottom, b) addition of N,N-diisopropylethylamine (1 equiv.). Spectra recorded in reaction solvent C_6H_5Cl. *Note: peaks at 2.29 and 6.81 ppm are due to 1,3,5-trimethylbenzene (added as internal standard).*

4.4 Reactivity in the presence of a hindered base

<u>In-situ NMR spectra of **7-H** made using **2** / excess hindered base (2,6-ditertbutyl-4-methylpyridine) after heating to 110°C (note the reaction is extremely slow at r.t.)</u>

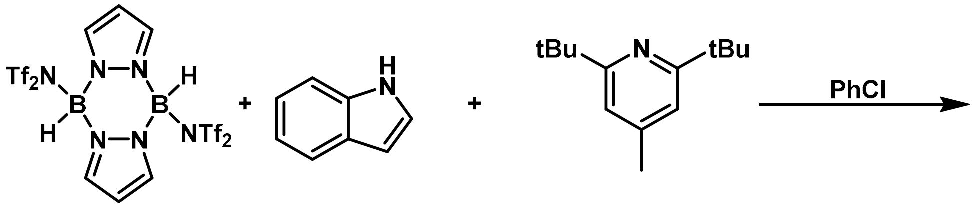

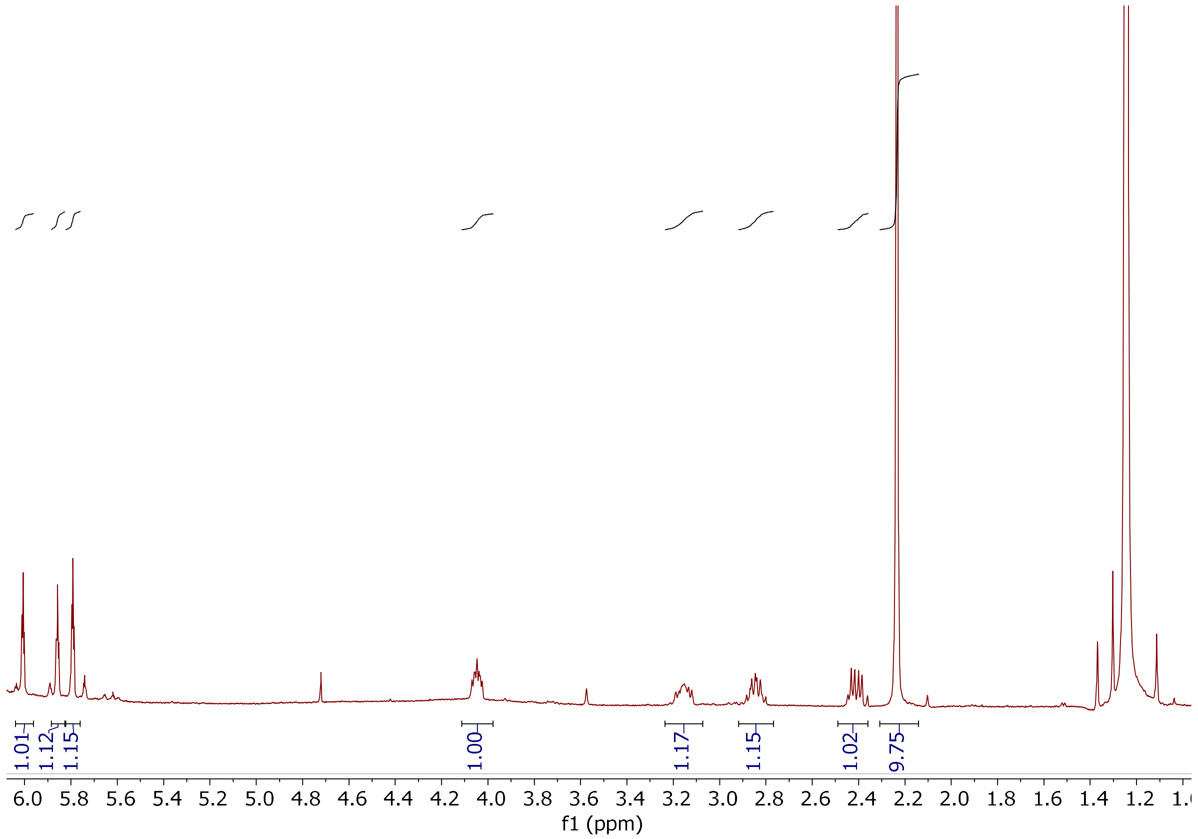

Figure S79: Partial ^{1}H NMR spectrum in PhCl (after heating 18 h at 110°C). This shows the diagnostic resonances for the formation of **7-H** (or a very close analogue with different substituents on B e.g. H/NTf_2 exchange). Note the identical indoline region to that for **7-H** shown in Fig. S54 – 55. The intense singlets in the aliphatic region are due to the hindered base.

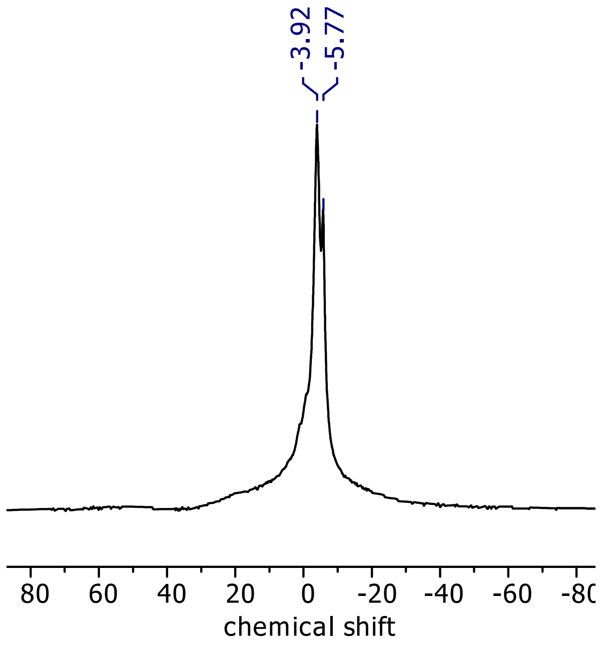

Figure S80: ^{11}B NMR spectrum in PhCl.

Synthesis of *bis*-Indole Pyrazabole 8

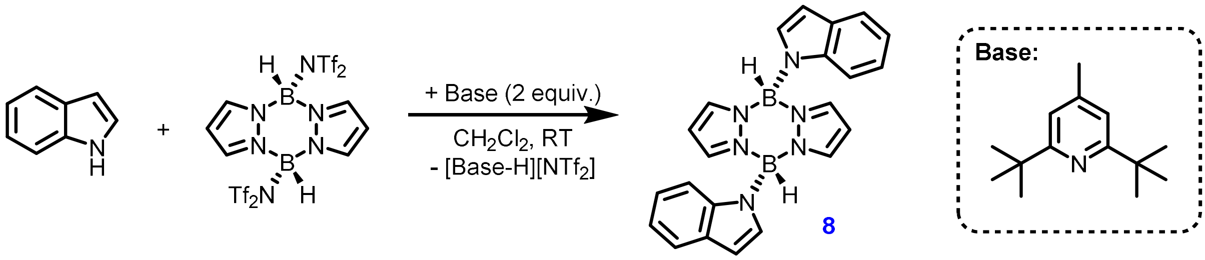

A solution of *bis*-NTf_2 pyrazabole **2** (0.296 g, 0.409 mmol, 1 equiv.), indole (0.096 g, 0.818 mmol, 2 equiv.) and 2,6-di-tert-butyl-4-methyl-pyridine (0.168 g, 0.818 mmol, 2 equiv.) in dichloromethane (2 mL) was stirred at room temperature for 5 minutes. The volatiles were removed *in vacuo*, and the residue was extracted with toluene (2 x 5 mL). The extracts were combined and reduced to a third of the initial volume and left at room temperature. After 18 hours, the supernatant was filtered off the colourless crystals, and again reduced to a third of the initial volume to obtain a second batch. The isolated crystals from both batches were dried *in vacuo* to give the product as a colourless crystalline solid (0.056 g, 0.14 mmol, 34 %). X-ray quality crystals were grown by layering a dichloromethane solution with pentane.

^{1}H NMR (400 MHz, C_6D_6) δ 7.83 (dt, $^3J_{HH}$ = 7 Hz, $^4J_{HH}$ = 1 Hz, 2H, ArC*H*), 7.29-7.19 (m, 6, ArC*H*), 7.03 (d, $^3J_{HH}$ = 3 Hz, 2H, NC*H*=CH), 6.79 (d, $^3J_{HH}$ = 3 Hz, 4H, Pyrazole N=C*H*-CH), 6.72 (d, $^3J_{HH}$ = 3 Hz, 2H, NCH=C*H*), 5.39 (t, $^3J_{HH}$ = 3 Hz, 2H, Pyrazole N=CH-C*H*);

^{1}H{^{11}B} NMR (400 MHz, C_6D_6) δ 7.83 (dt, $^3J_{HH}$ = 7 Hz, $^4J_{HH}$ = 1 Hz, 2H, ArC*H*), 7.29-7.19 (m, 4H, ArC*H*), 7.03 (d, $^3J_{HH}$ = 3 Hz, 2H, NC*H*=CH), 6.79 (d, $^3J_{HH}$ = 3 Hz, 4H, Pyrazole N=C*H*-CH), 6.72 (d, $^3J_{HH}$ = 3 Hz, 2H, NCH=C*H*), 5.39 (t, $^3J_{HH}$ = 3 Hz, 2H, Pyrazole N=CH-C*H*) 5.13 (s, 2H, B*H*);

^{11}B NMR (128 MHz, C_6D_6) δ -4.0(br, Pyrz*B*H(Indole));

^{11}B{^{1}H} NMR (128 MHz, C_6D_6) δ -3.7 (s, Pyrz*B*H(Indole));

^{13}C{^{1}H} NMR (126 MHz, C_6D_6) δ 139.7, 136.5, 131.6, 130.5, 121.2, 121.0, 119.8, 112.0, 106.9, 103.7 ppm.

HRMS (EI$^+$) *m/z* calcd for $C_{22}H_{20}B_2N_6$: 390.1930 [M]$^{\cdot+}$, found 390.1938.

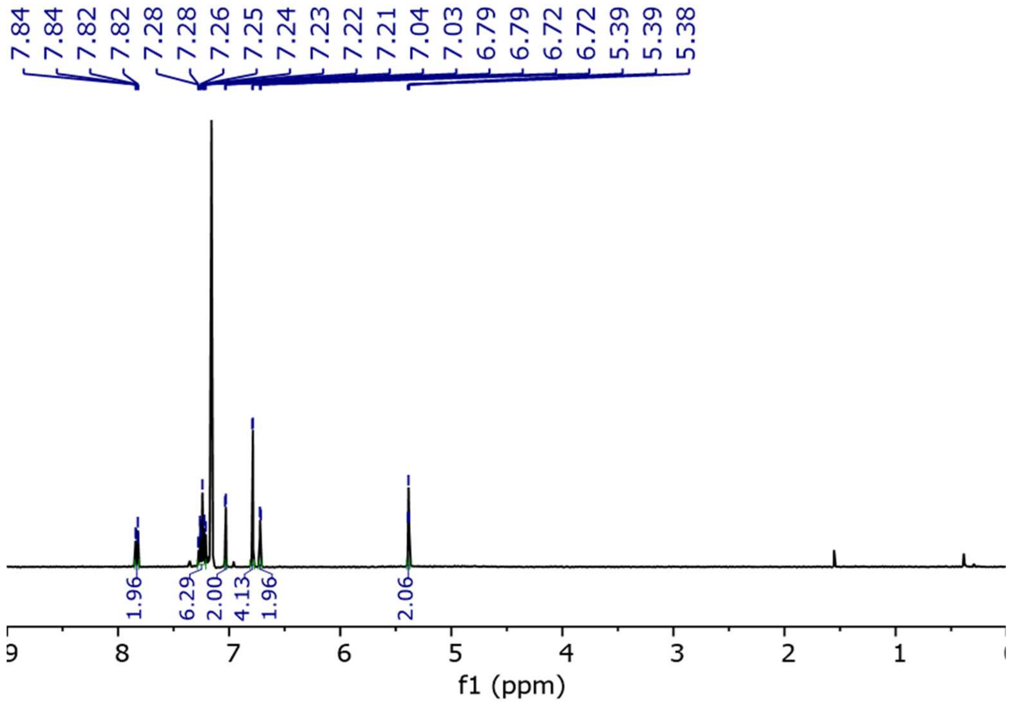

Figure S81: ^{1}H NMR spectrum of *bis*-indole pyrazabole (**8**) in C_6D_6.

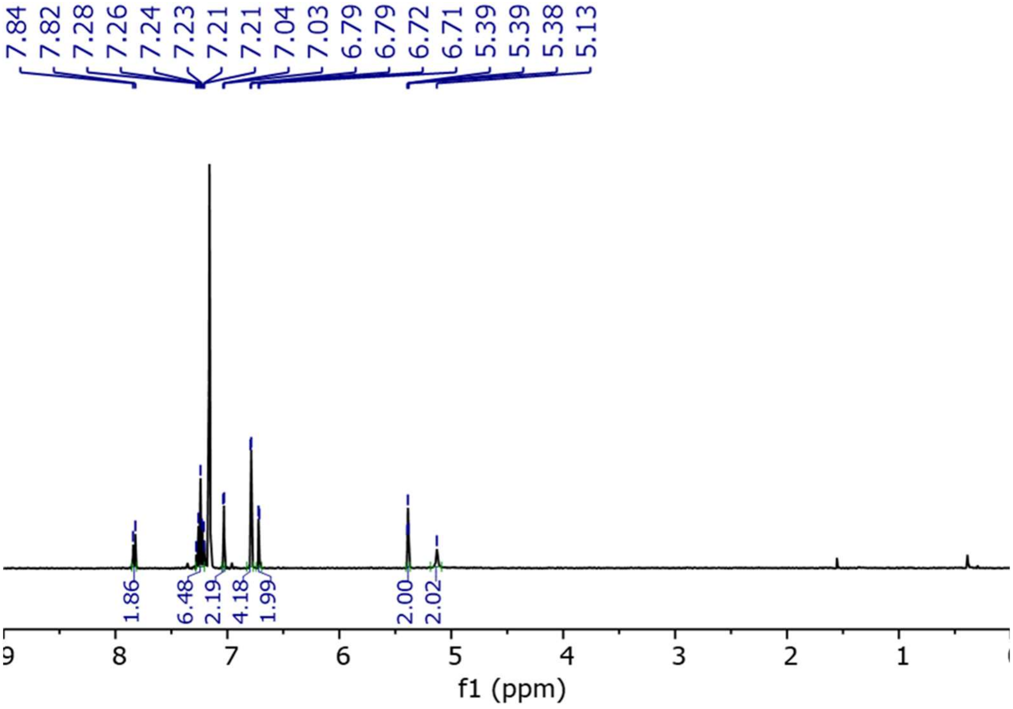

Figure S82: $^{1}H\{^{11}B\}$ NMR spectrum of *bis*-indole pyrazabole (**8**) in C_6D_6.

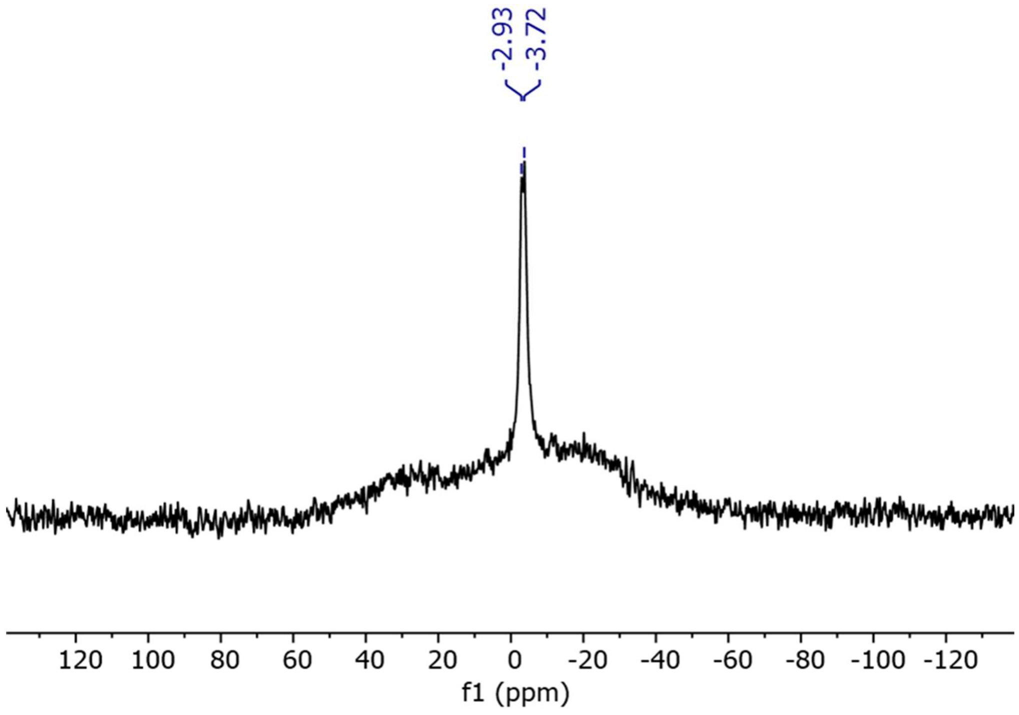

Figure S83: ^{11}B NMR spectrum of *bis*-indole pyrazabole (**8**) in C_6D_6.

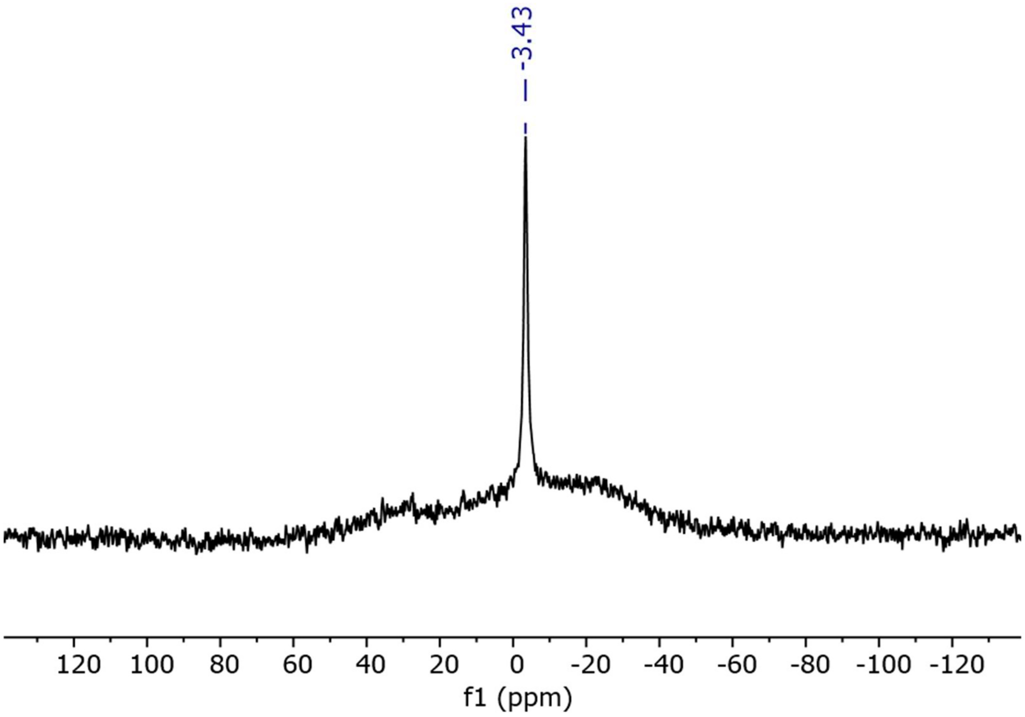

Figure S84: $^{11}B\{^1H\}$ NMR spectrum of *bis*-indole pyrazabole (**8**) in C_6D_6.

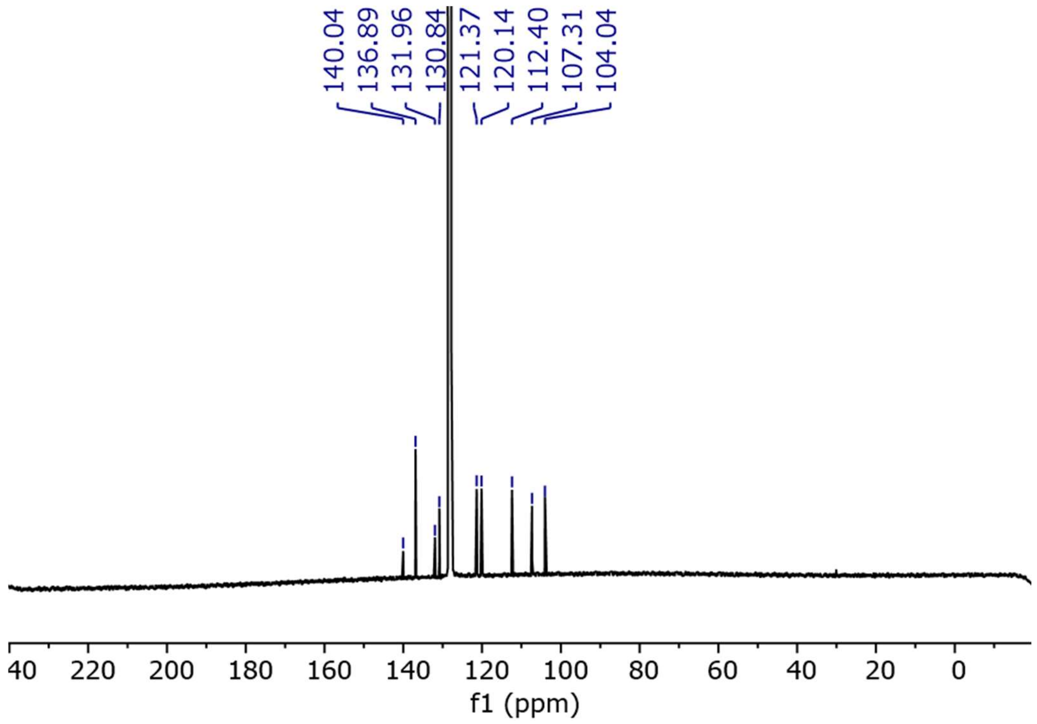

Figure S85: $^{13}C\{^1H\}$ NMR spectrum of *bis*-indole pyrazabole (**8**) in C_6D_6.

4.5 Reaction of N-H Indoline and 2 in the Presence of a Exogenous Base

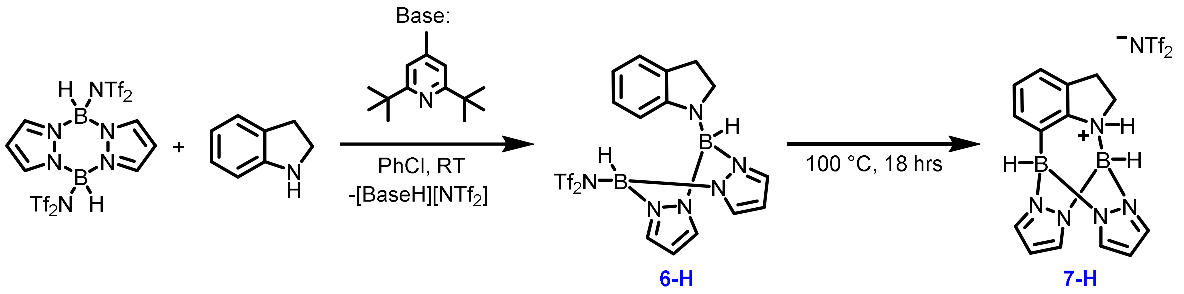

A J. Young's NMR tube was charged with *bis*-NTf_2 pyrazabole **2** (0.079 g, 0.110 mmol, 1.1 equiv.), indoline (0.011 mL, 0.10 mmol, 1 equiv.) and 2,6-di-tert-butyl-4-methyl-pyridine (0.020 g, 0.10 mmol, 1 equiv.), and dissolved in chlorobenzene (0.5 mL). The solution was left at room temperature (with rotation and periodic analysis by NMR spectroscopy) to form **6-H**, then heated to 100 °C overnight. NMR spectra were recorded and the major product (relative to 1,3,5-trimethyl benzene internal standard) matched the C7-borylated intermediate **7-H**.

In situ NMR Spectra of N-Pyrazabole Indoline Intermediate (**6-H**)

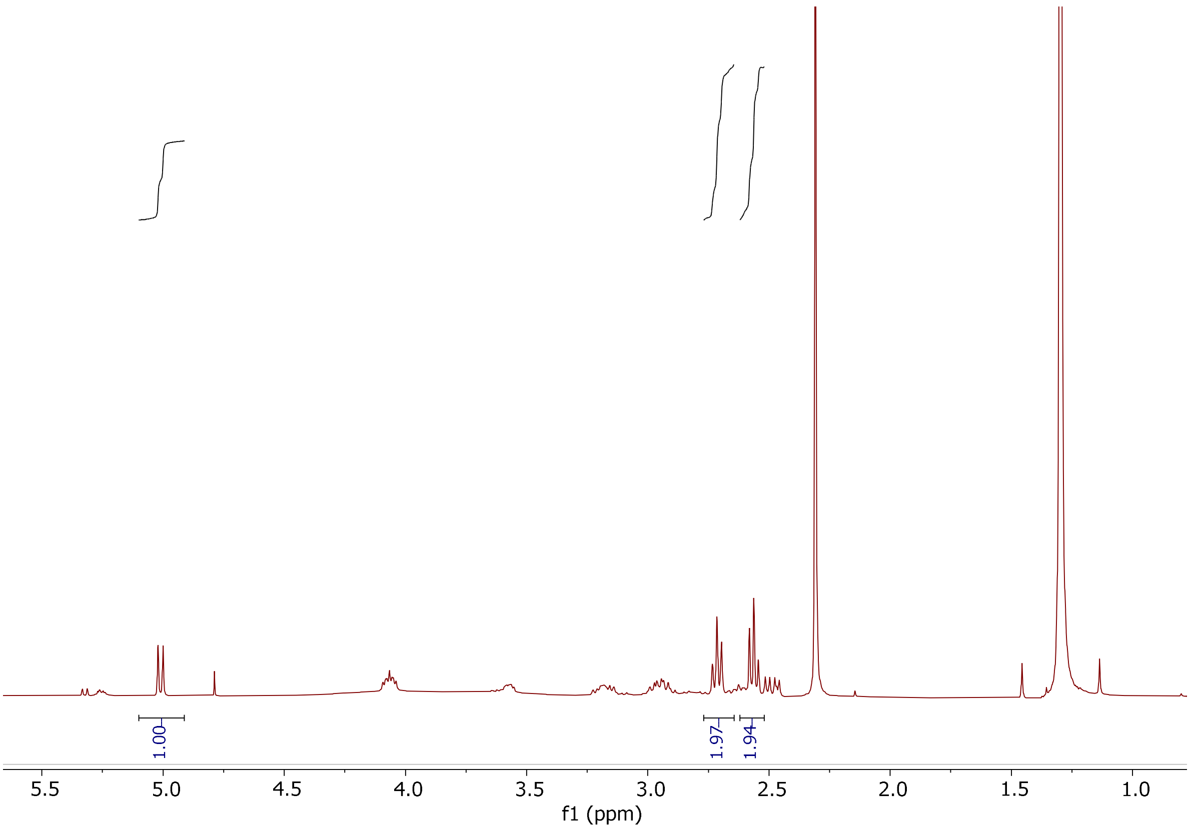

Figure S86: ^1H NMR spectrum of key region of the reaction of N-H indoline, **2** and base in PhCl after 18 hours at room temperature. Note the two triplets of 2H intensity between 2.5 and 2.7 ppm are also observed in the N-H-indole / **3** reaction after 18 h at rt. The doublet at ca. 5 ppm is assigned to a pyrazabole resonance and is also observed in Fig S54. Other key resonances indicate formation of **7-H** is occurring slowly at room temperature, while the intense singlets are due to the base.

NMR Spectra of C7-Borylated Intermediate (**7-H**) (Reaction after 18 hours 100 °C).

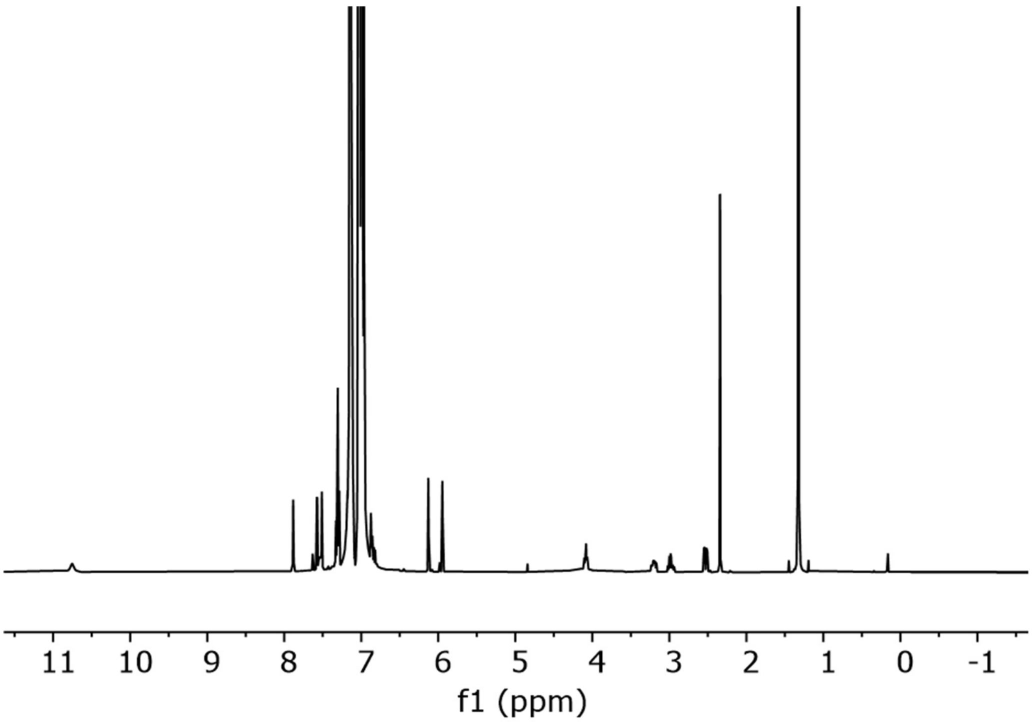

Figure S87: ^{1}H NMR spectrum of reaction of N-H indoline, **2** and base after 18 hours 100 °C, with **7-H** as the major product. Spectrum recorded in reaction solvent C_6H_5Cl. *Note: the sharp singlets in the aliphatic region are due to protonated base, as is the broad singlet at 10.7 ppm*

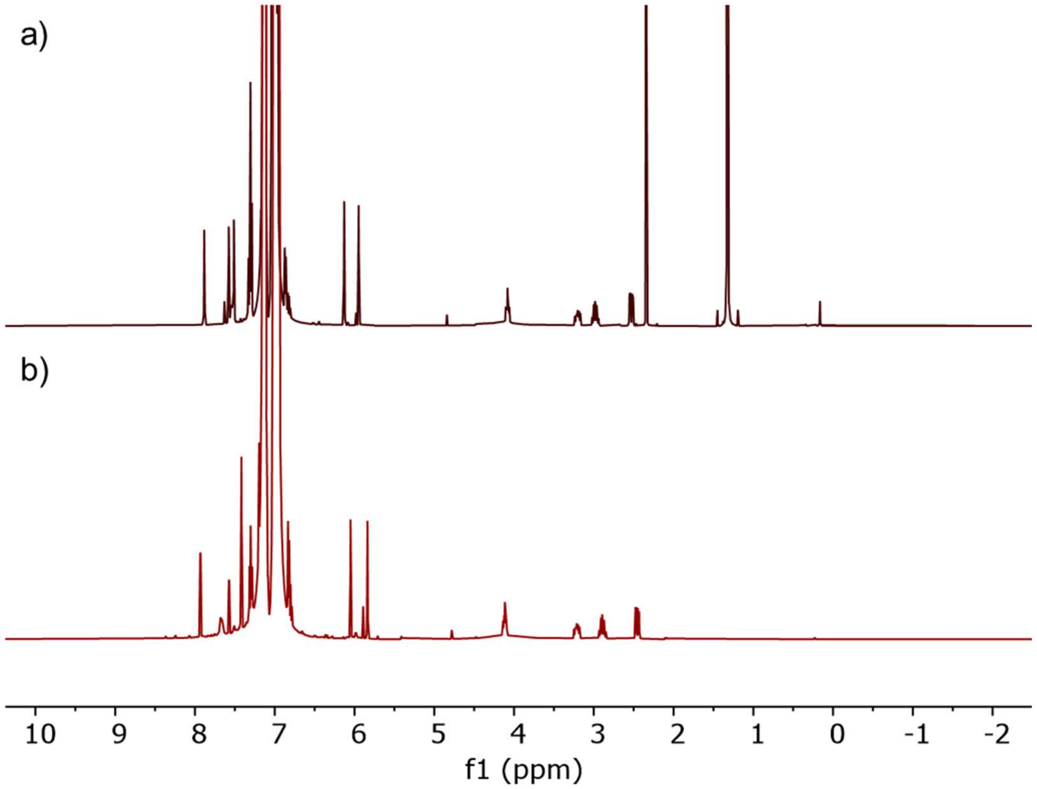

Figure S88: ^{1}H NMR spectrum of reaction of N-H indoline, **2** and base after 18 hours at 100 °C (top, a) and reaction of N-H-indole and **3** after 18 hours 100 °C (bottom, b) with **7-H** as the major product in both. Spectra recorded in reaction solvent C_6H_5Cl.

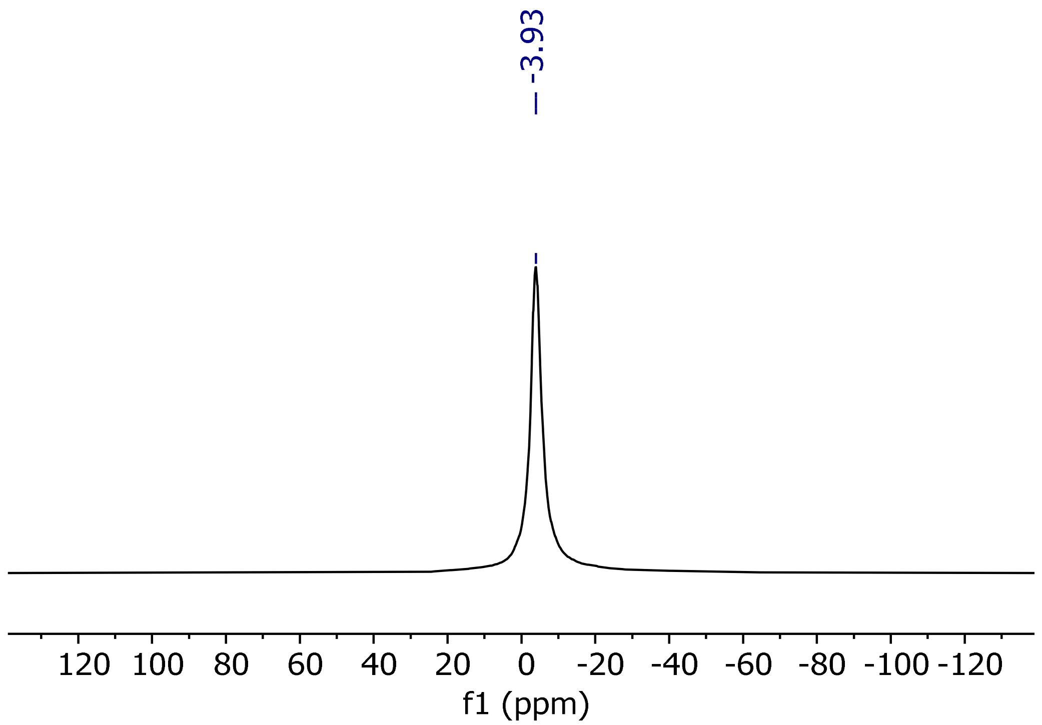

Figure S89: ^{11}B NMR spectrum of reaction of N-H indoline, **2** and base after 18 hours 100 °C, with **7-H** as the major product. Spectrum recorded in reaction solvent C_6H_5Cl.

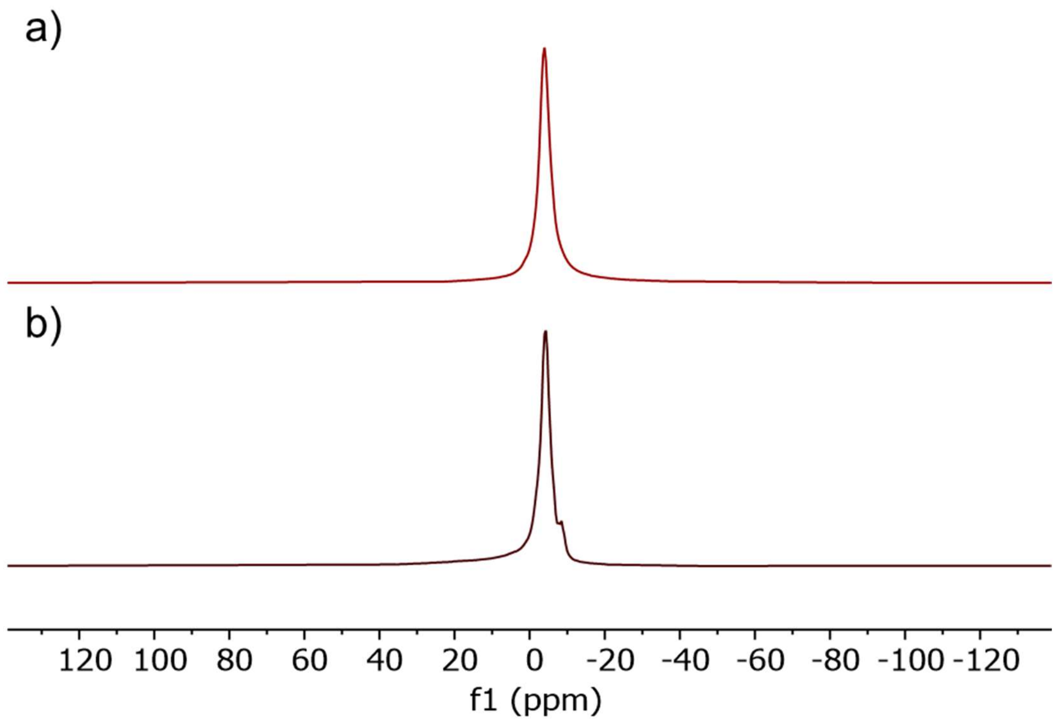

Figure S90: ^{11}B NMR spectrum of reaction of N-H indoline, **2** and base after 18 hours 100 °C (top, a) and reaction of indole and **3** after 18 hours 100 °C (bottom, b) with **7-H** as the major product in both. Spectra recorded in reaction solvent C_6H_5Cl.

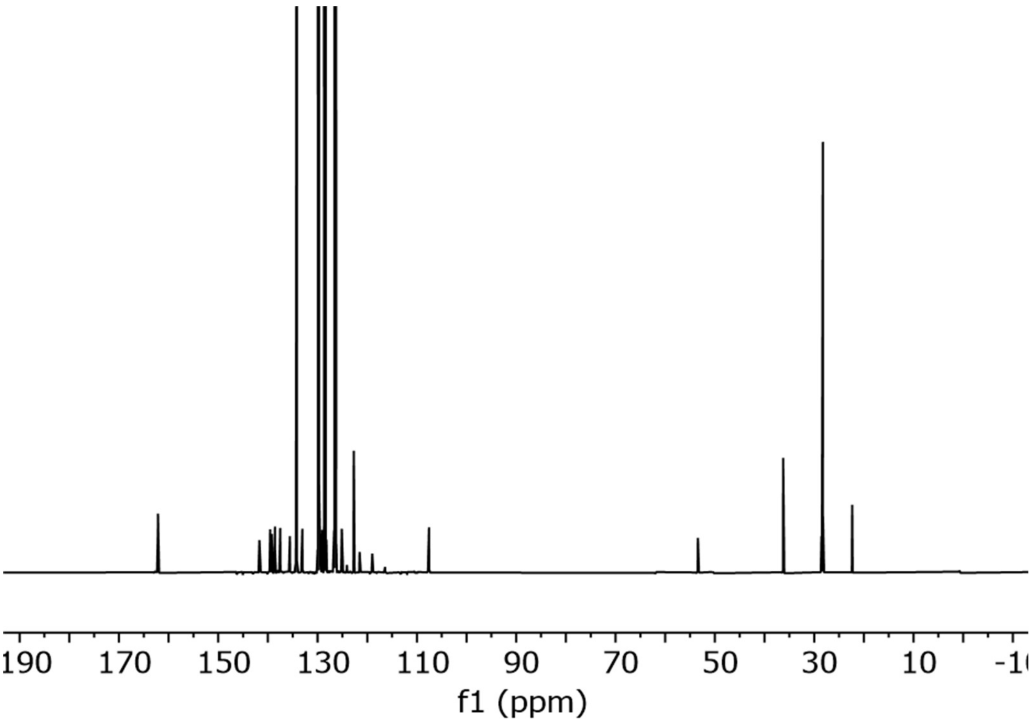

Figure S91: $^{13}C\{^1H\}$ NMR of reaction of N-H indoline, **2** and base after 18 hours 100 °C, with **7-H** as the major product. Spectrum recorded in reaction solvent C_6H_5Cl.

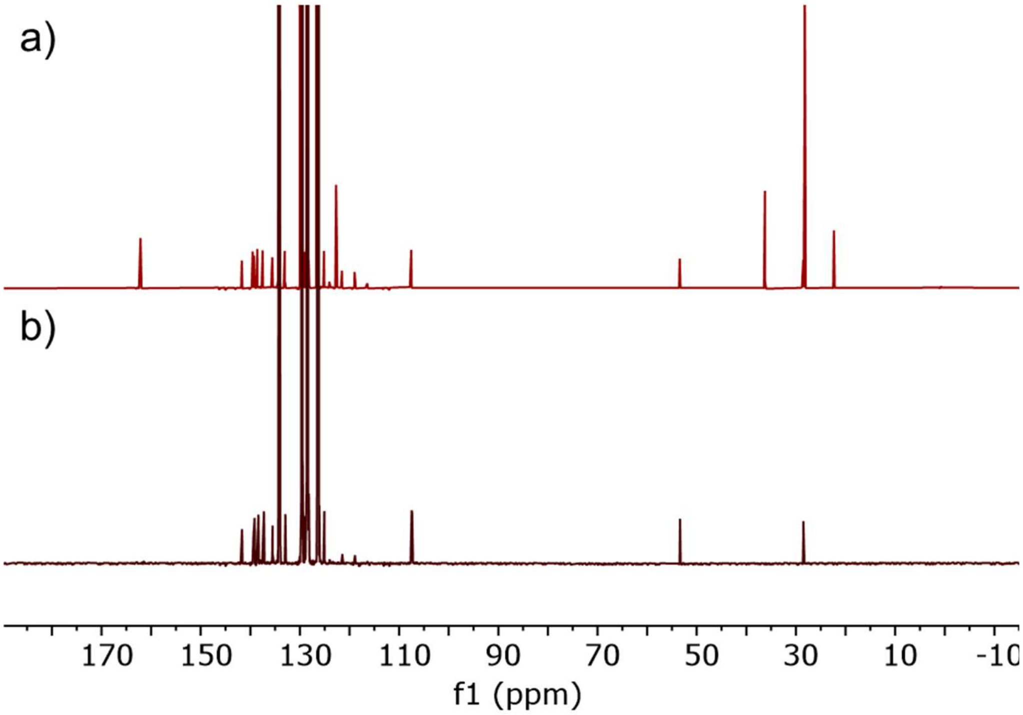

Figure S92: $^{13}C\{^1H\}$ NMR spectrum of reaction of N-H indoline, **2** and base after 18 hours 100 °C (top, a) and reaction of indole and **3** after 18 hours 100 °C (bottom, b) with **7-H** as the major product in both. Spectra recorded in reaction solvent C_6H_5Cl.

5.0 Reactivity of N-R Indoles with Pyrazaboles

5.1 Reactivity with N-Me Indole

Formation of compound **9**

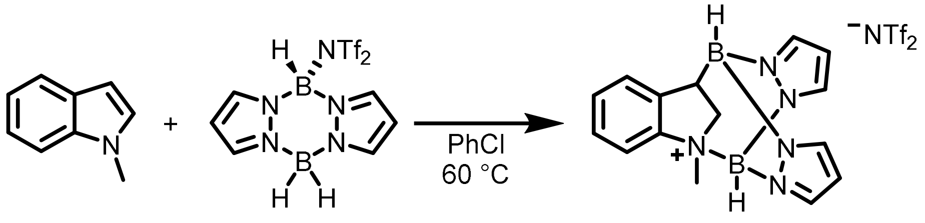

9

A solution of **3** was prepared by reacting pyrazabole **1** (0.202 g, 1.28 mmol, 1 equiv.) with $HNTf_2$ (0.359 g, 1.28 mmol, 1 equiv.) in PhCl (5 mL), and stirred at room temperature until gas evolution (H_2) ceased. Following addition of N-Me Indole (0.160 mL, 1.29 mmol, 1 equiv.), the reaction was heated to 60 °C for 10 minutes. The volatiles were removed *in vacuo*, resulting in a viscous residue. The residue was washed with pentane (7 x 5 mL) until the product was obtained as an off-white powder (0.633 g, 1.11 mmol, 87%). X-ray quality crystals were grown via chlorobenzene / hexane vapour diffusion at -30 °C.

^{1}H NMR (400 MHz, CD_2Cl_2) δ 8.17 (d, $^{3}J_{HH}$ = 3 Hz, 1H, N=C*H*CH), 8.06 (d, $^{3}J_{HH}$ = 3 Hz, 1H, N=C*H*CH), 7.46 (d, $^{3}J_{HH}$ = 3 Hz, 1H, N=C*H*CH), 7.44 (d, $^{3}J_{HH}$ = 3 Hz, 1H, N=C*H*CH), 7.17 – 7.05 (m, 4H, ArC*H*), 6.69 (t, $^{3}J_{HH}$ = 3 Hz, 1H, N=CHC*H*), 6.04 (t, $^{3}J_{HH}$ = 3 Hz, 1H, N=CHC*H*), 3.41 (dd, $^{3}J_{HH}$ = 9 Hz, $^{2}J_{HH}$ = 5 Hz, 1H, NCH_2-C(H)B), 3.29 (s, 3H, NCH_3), 3.04 (dd, $^{3}J_{HH}$ = 9 Hz, $^{2}J_{HH}$ = 5 Hz, 1H, NCH_2-C(*H*)B), 2.54 (d, $^{3}J_{HH}$ = 9 Hz, 1H, NCH_2-C(H)B);

^{1}H{^{11}B} NMR (500 MHz, CD_2Cl_2) δ 8.17 (d, $^{3}J_{HH}$ = 3 Hz, 1H, N=C*H*CH), 8.06 (d, $^{3}J_{HH}$ = 3 Hz, 1H, N=C*H*CH), 7.46 (d, $^{3}J_{HH}$ = 3 Hz, 1H, N=C*H*CH), 7.44 (d, $^{3}J_{HH}$ = 3 Hz, 1H, N=C$_{H}$CH), 7.19 – 7.04 (m, 4H, ArC*H*), 6.69 (t, $^{3}J_{HH}$ = 3 Hz, 1H, N=CHC*H*), 6.04 (t, $^{3}J_{HH}$ = 3 Hz, 1H, N=CHC*H*), 4.45 – 3.74 (br m, 2H, B*H*), 3.41 (dd, $^{3}J_{HH}$ = 9 Hz, $^{2}J_{HH}$ = 5 Hz, 1H, NCH_2-C(H)B), 3.29 (s, 3H, NCH_3), 3.04 (dd, $^{3}J_{HH}$ = 9 Hz, $^{2}J_{HH}$ = 5 Hz, 1H, NCH_2-C(*H*)B), 2.54 (d, $^{3}J_{HH}$ = 9 Hz, 1H, NCH_2-C(H)B);

^{11}B NMR (128 MHz, CD_2Cl_2) δ 0.9 (d, $^{1}J_{BH}$ = 133 Hz), -3.3 (d, $^{1}J_{BH}$ = 121 Hz);

^{11}B{^{1}H} NMR (128 MHz, CD_2Cl_2) δ 0.9 (s), -3.3 (s);

^{13}C{^{1}H} NMR (126 MHz, CD_2Cl_2) δ 146.2, 141.8, 140.1, 139.8, 139.7, 138.5, 129.0, 126.9, 125.3, 120.4 (q, $^{1}J_{CF}$ = 321 Hz, SCF_3), 116.3, 109.0, 108.0, 73.1, 47.5, 32.5;

^{19}F NMR (376 MHz, CD_2Cl_2) δ -79.4 (s) ppm.

Attempts to collect air sensitive mass spectrometry data on this sample did not yield any useful ions representing this product.

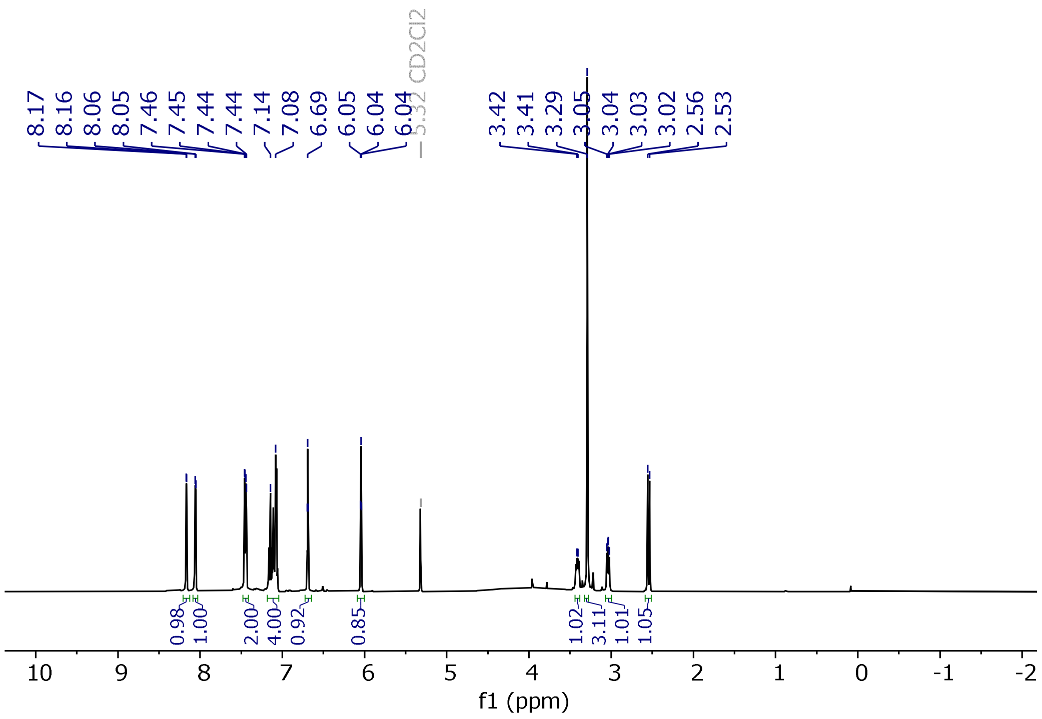

Figure S93: ^{1}H NMR spectrum of hydroborated product **9** in CD_2Cl_2.

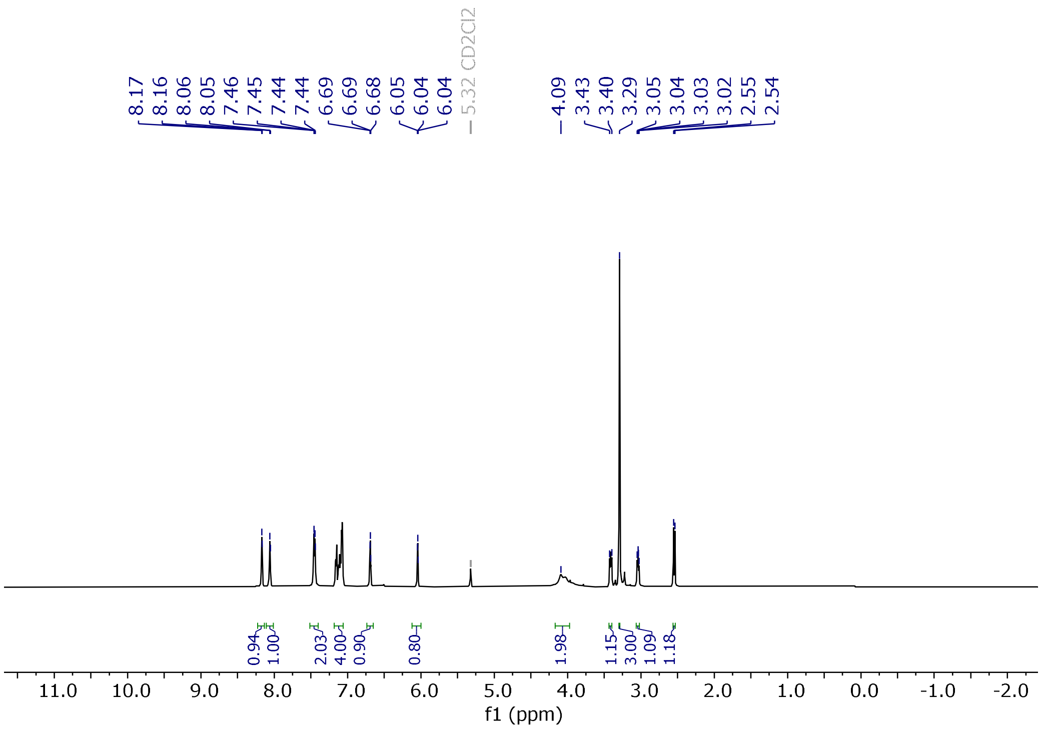

Figure S94: ^{1}H{^{11}B} NMR spectrum of hydroborated product **9** in CD_2Cl_2.

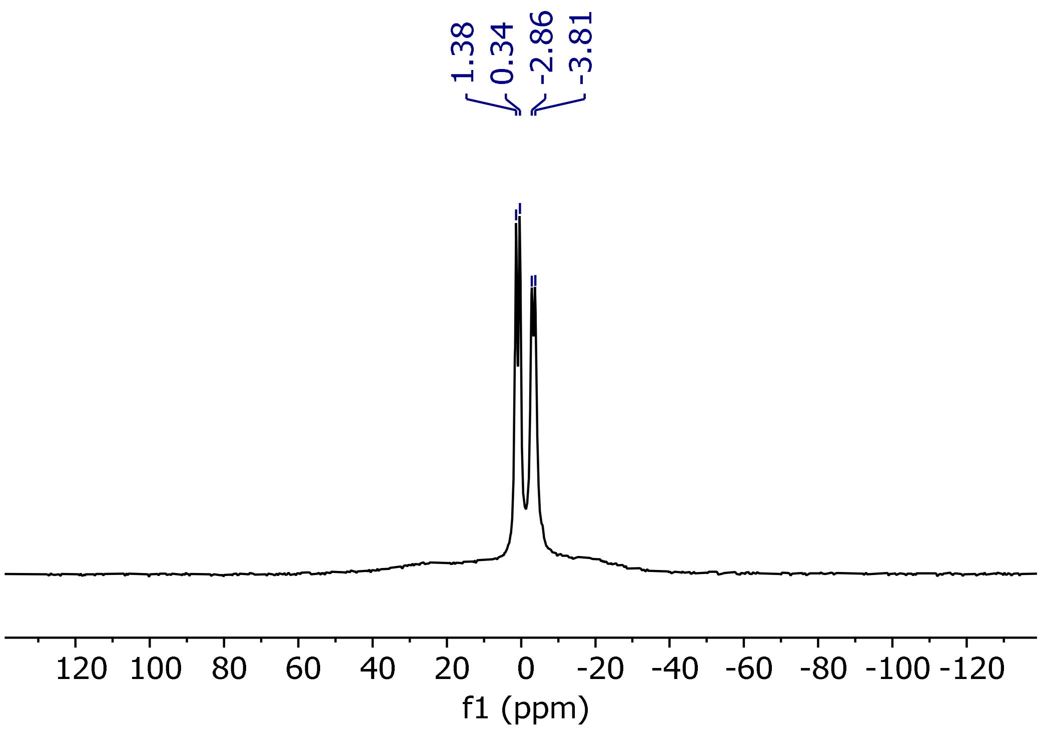

Figure S95: ^{11}B NMR spectrum of hydroborated product **9** in CD_2Cl_2.

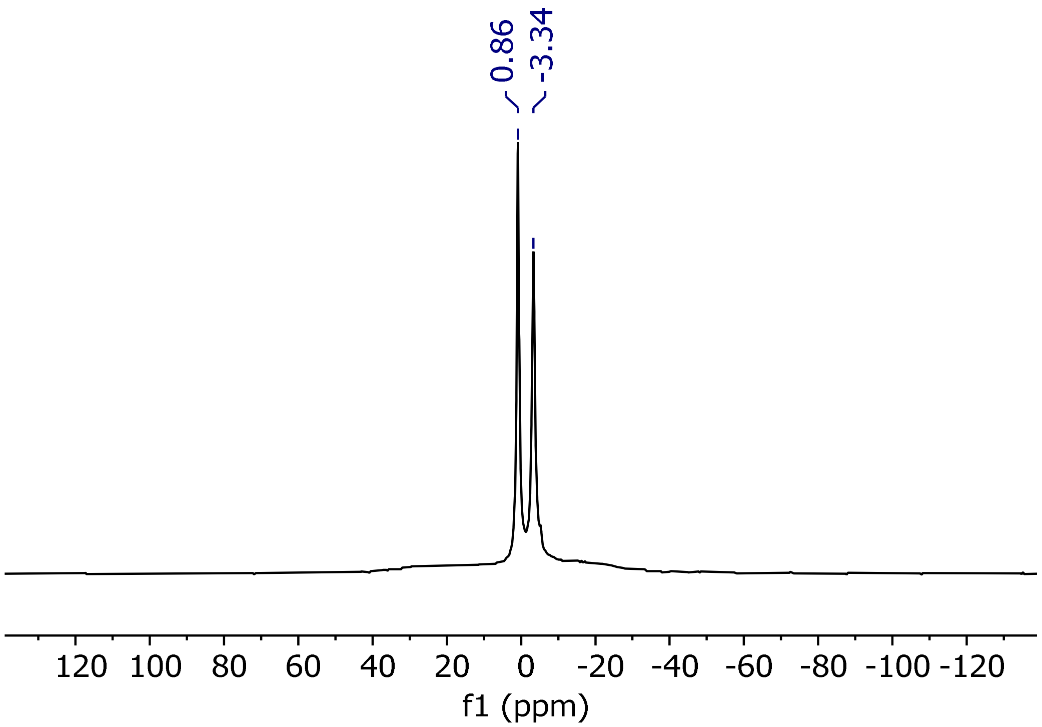

Figure S96: $^{11}B\{^1H\}$ NMR spectrum of hydroborated product **9** in CD_2Cl_2.

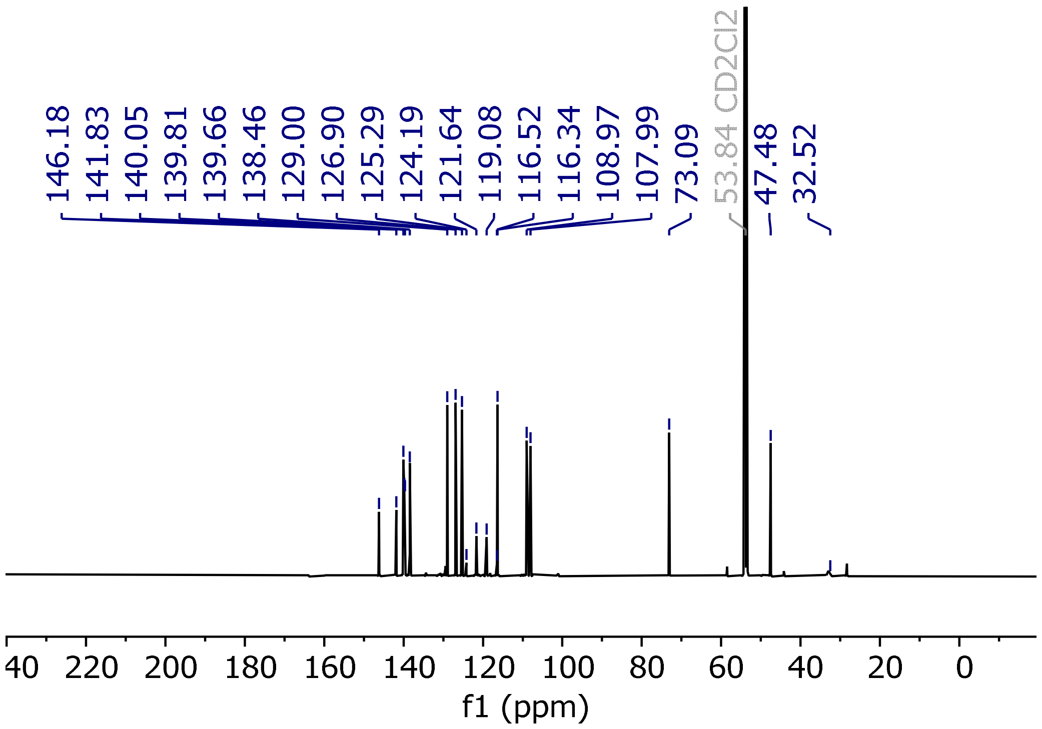

Figure S97 $^{13}C\{^1H\}$ NMR spectrum of hydroborated product **9** in CD_2Cl_2.

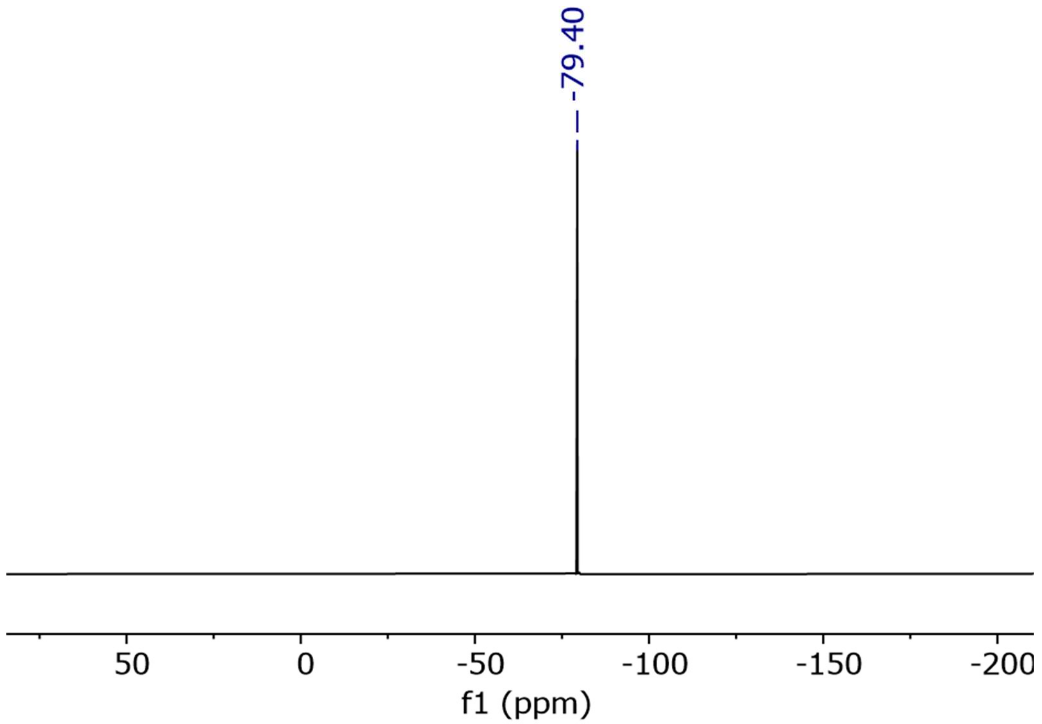

Figure S98: ^{19}F NMR spectrum of hydroborated product **9** in CD_2Cl_2.

Attempts to convert **9** by heating for prolonged periods with/without exogenous base (DBP)

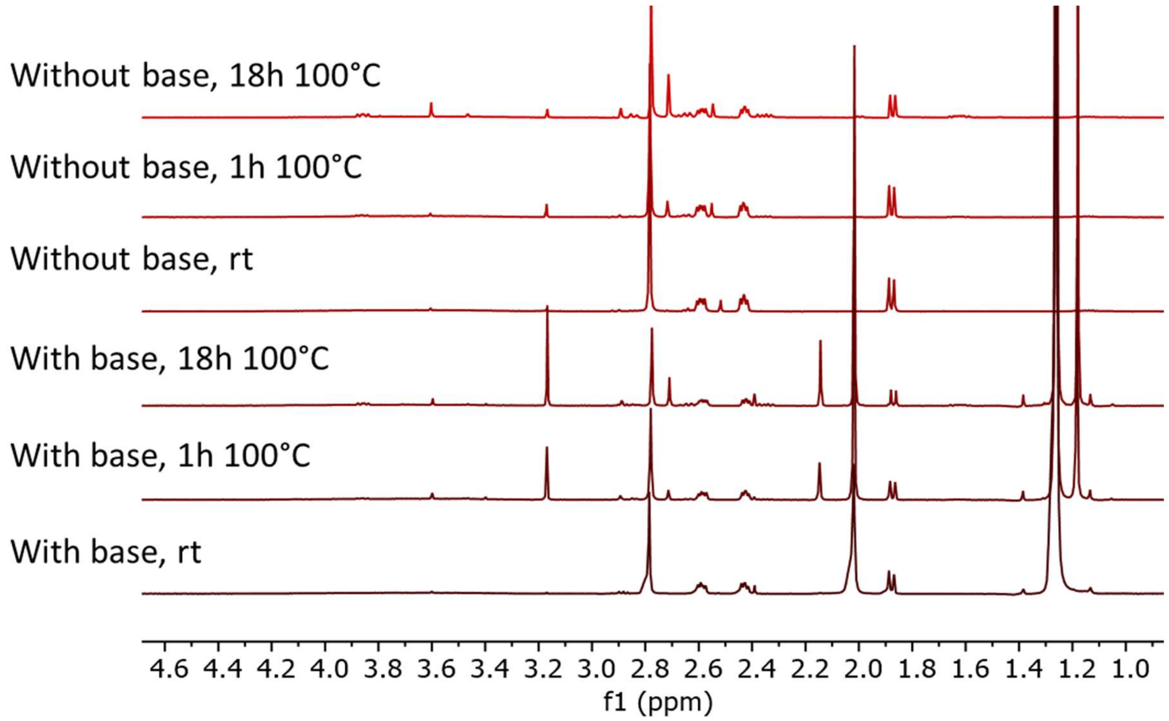

Figure S99: ^{1}H NMR spectrum of **9** in PhCl heated for prolonged periods. This shows minimal change to the indoline region after heating at 100°C, precluding significant N-B dissociation and C4-borylation.

Attempts to hydroborate **N-Me-indole** with **1**

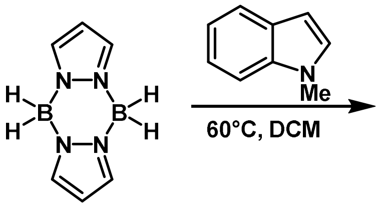

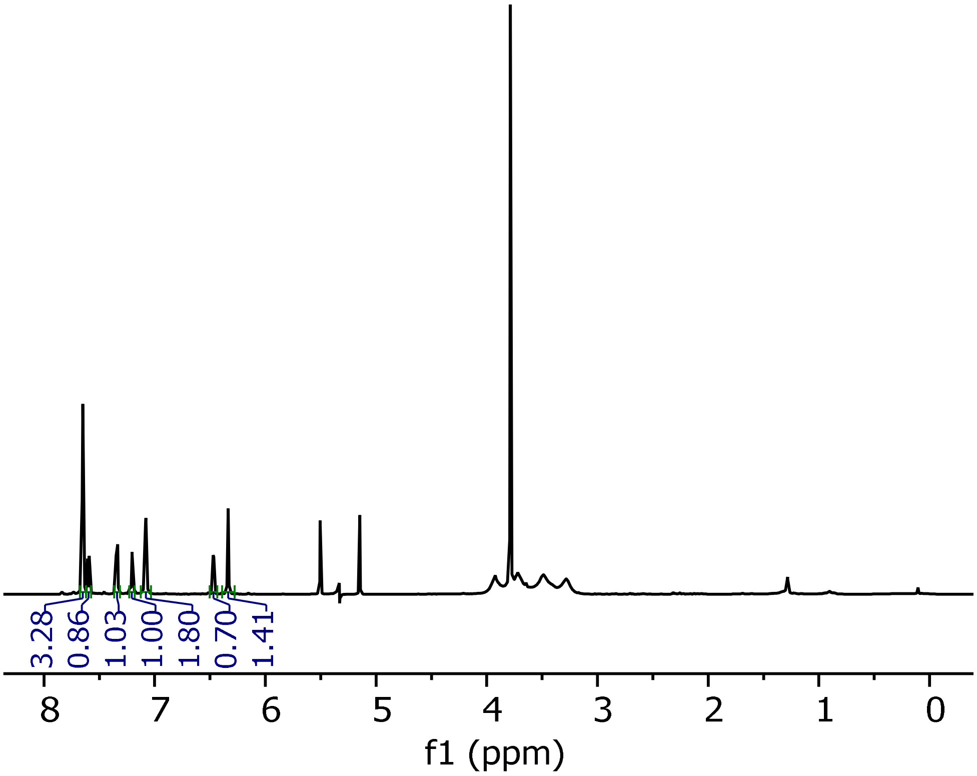

Figure S100: ^{1}H NMR spectrum of **1** + N-Me-indole after heating overnight at 60°C (in protio DCM with solvent suppression).

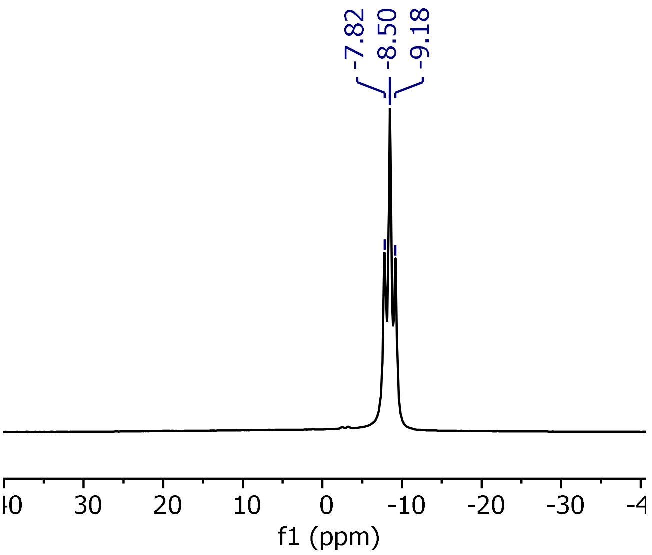

Figure S101: ^{11}B NMR spectrum of **1** + N-Me-indole after heating overnight at 60°C (in protio DCM with solvent suppression).

5.3 Synthesis of N-TMS Indole

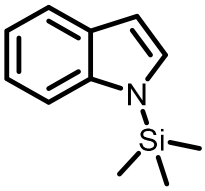

To a solution of indole (2.0 g, 17.06 mmol) in THF (5 mL) was added *n*-BuLi (11.8 mL, 1.6 M in hexanes, 19 mmol, 1.1 equiv.) dropwise at 0 °C. After stirring at 0 °C for 1 hour, chlorotrimethylsilane (2.6 mL, 20 mmol, 1.2 equiv.) was added dropwise. The resulting suspension was warmed to room temperature. The volatiles were removed *in vacuo* and the resulting slurry was extracted with pentane (2 x 5 mL). The combined pentane phases were concentrated, yielding the product as a colourless oil (3.025 g, 15.98 mmol, 94%). Analytical data are in accordance with literature values.[2]

^{1}H NMR (500 MHz, CH_2Cl_2) δ 7.56 (d, $^3J_{HH}$ = 7.5 Hz, 1H), 7.46 (d, $^3J_{HH}$ = 7.5 Hz, 1H), 7.16-7.00 (m, 4H), 6.53 d, $^3J_{HH}$ = 3 Hz, 1H), 0.49 (s, 9H) ppm.

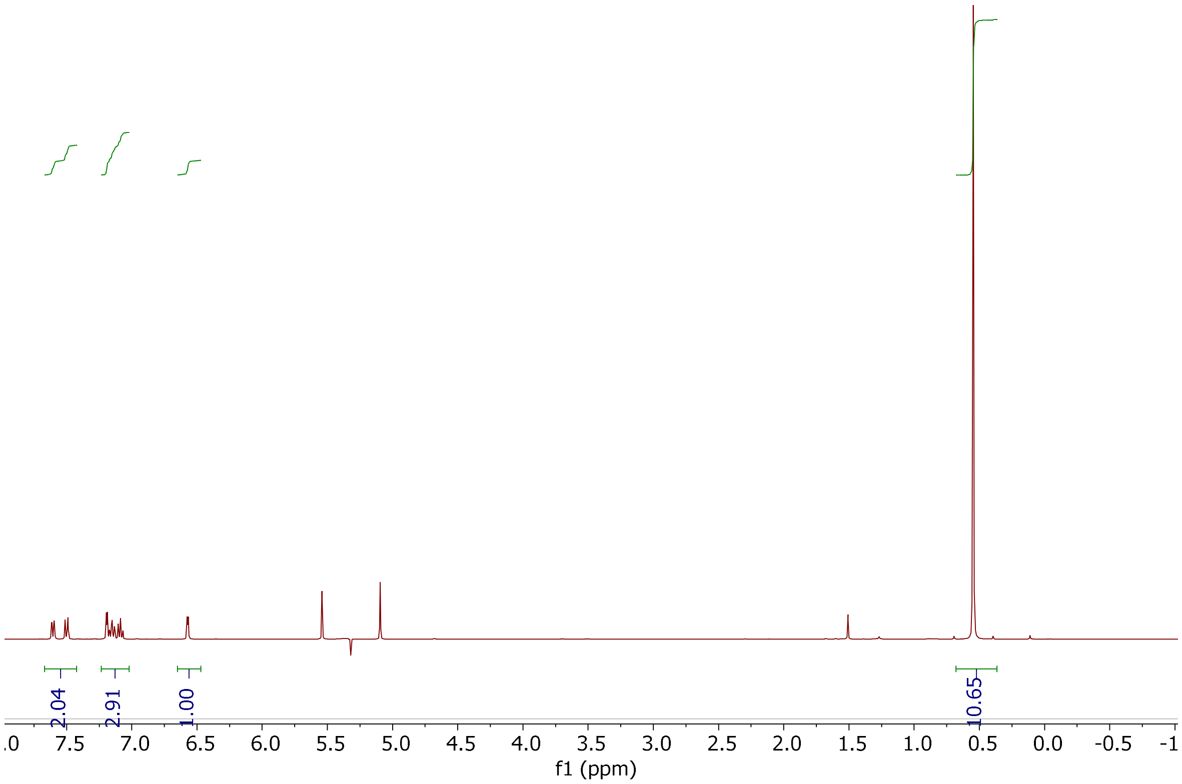

Figure S102: ^{1}H NMR spectrum of N-TMS-indole in CH_2Cl_2 (solvent suppression)

5.2 Synthesis of N-TIPS Indole

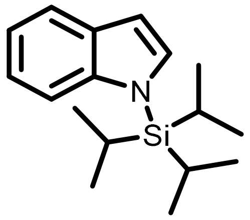

To a solution of indole (0.607 g, 5.18 mmol) in THF (20 mL) was added *n*-BuLi (2.9 mL, 1.6 M in hexanes, 4.6 mmol, 0.9 equiv.) dropwise at 0 °C. After stirring at 0 °C for 1 hour, chlorotrimethylsilane (0.88 mL, 4.1 mmol, 0.8 equiv.) was added dropwise. The resulting suspension was warmed to room temperature. After addition of water (20 mL), the reaction mixture was extracted with EtOAc (3 x 20 mL), and the combined organic phases dried *in vacuo*. The resulting residue was taken up in heptane (20 mL) and washed with MeOH (2 x 10 mL). The organic phase was dried over $MgSO_4$, filtered and concentrated, yielding the product as a viscous colourless oil (0.810 g, 2.73 mmol, 67%). Analytical data are in accordance with literature values.[3]

^{1}H NMR (500 MHz, $CDCl_3$) δ 7.63 (d, $^3J_{HH}$ = 7.5 Hz, 1H), 7.51 (d, $^3J_{HH}$ = 8 Hz, 1H), 7.25 (d, $^3J_{HH}$ = 3 Hz, 1H), 7.12 (m, 2H), 6.62 (d, $^3J_{HH}$ = 3Hz, 1H), 1.71 (sept, $^3J_{HH}$ = 8z, 3H), 1.15 (d, $^3J_{HH}$ = 8 Hz, 18 H) ppm.

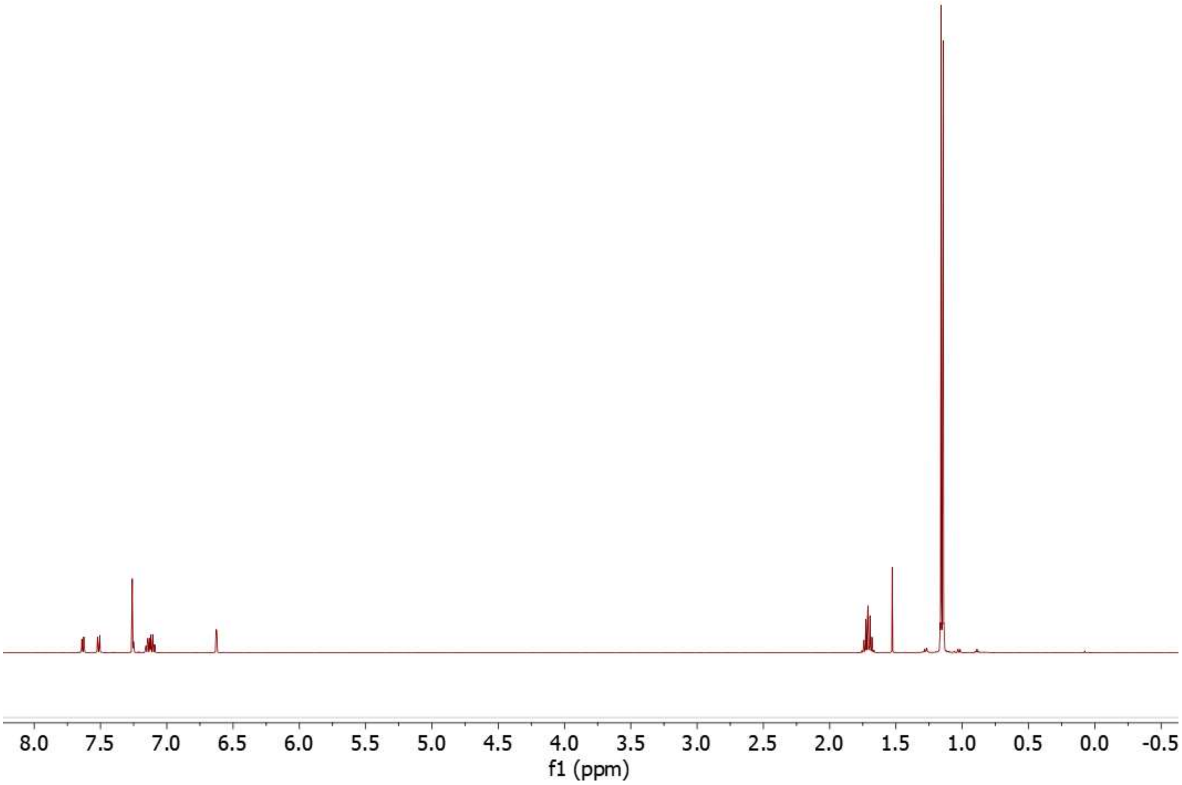

Figure S103: ^{1}H NMR spectrum of N-TIPS-indole in $CDCl_3$

5.4 Hydroboration of N-TMS Indole to 10

Combining a 1:1 mixture of **3** and TMS-indole led, after 1 h stirring at room temperature, to the clean hydroboration product analogous to **9**. The assignment is based on comparison to product **9** which has a closely comparable NMR spectrum (excluding the TMS Vs Me change).

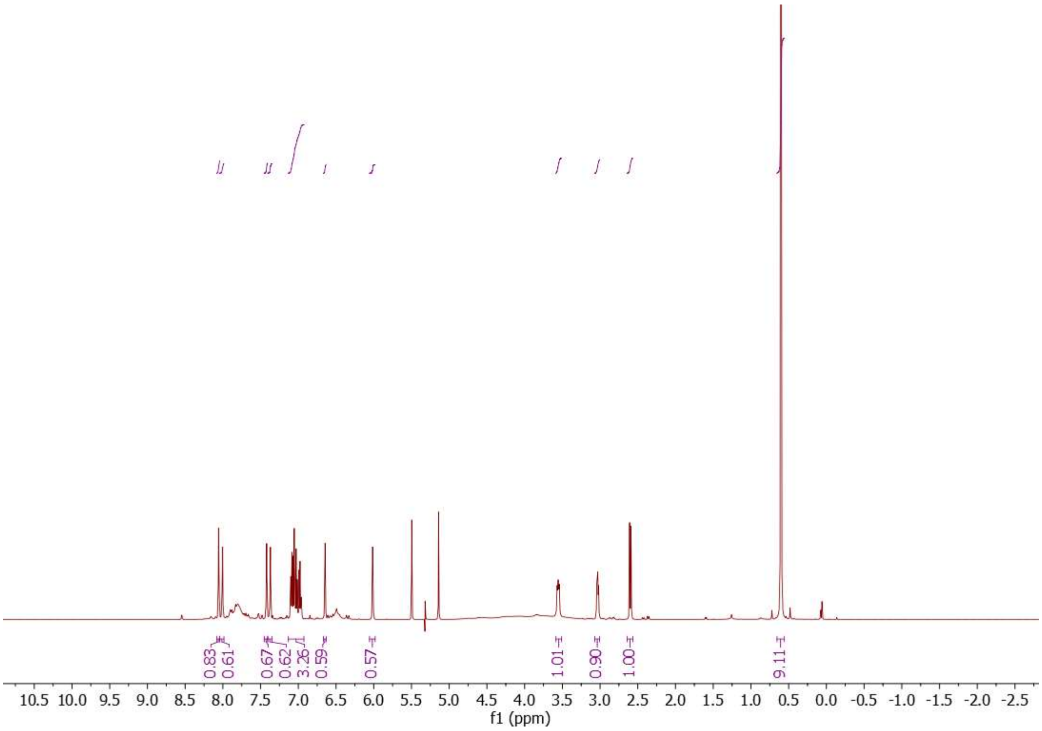

Figure S104: ^1H NMR spectrum of N-TMS-indole + **3** after 1 h in CH_2Cl_2 (with solvent suppression)

5.6 C7-Borylation of N-SiR$_3$ Indole to form 7-H

On heating with **3** with TIPS-indole in PhCl no intermediates are observed, the spectra obtained after 7 h heating at 100°C indicate formation of **7-H** along with formation of TIPS-H by-product.

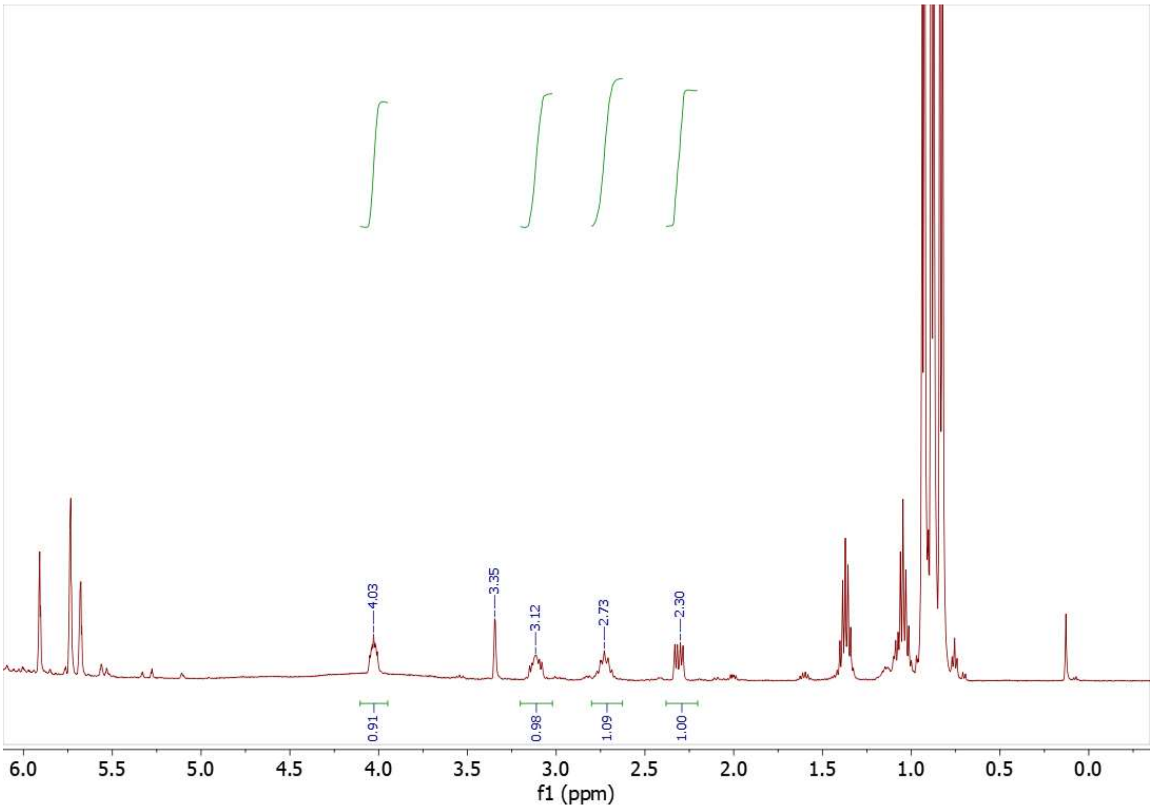

Figure S105: ^{1}H NMR spectrum of N-TIPS-indole + **3** after 7 h at 100°C in PhCl. The peak picked multiplets are identical to that for **7-H** in the same solvent and are attributable to the four diastereotopic indolines resonances. The singlet at 3.35 ppm is due to TIPSH

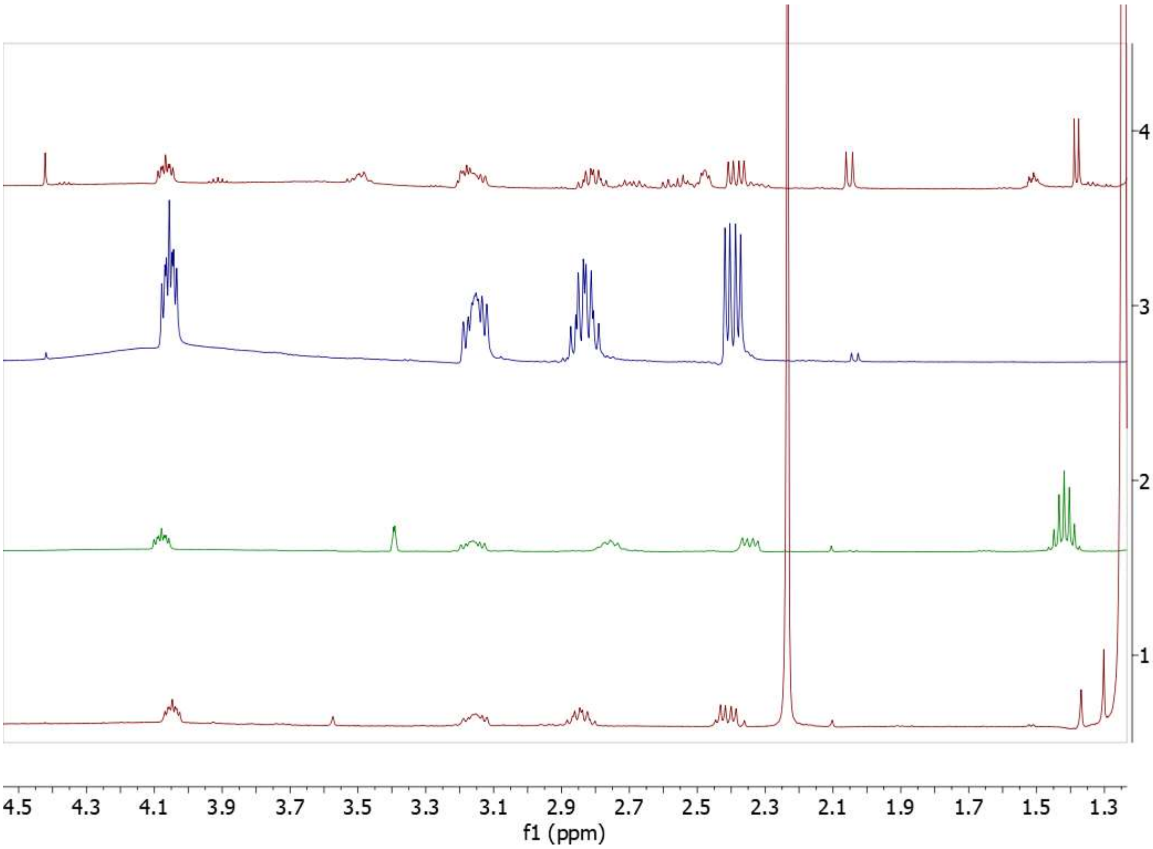

Figure S106: Stack plot showing in-situ ^{1}H NMR spectra for **7-H** made via the four different routes, each spectra contains the four diagnostic resonances for the diastereotopic indoline resonances for **7-H**.

Top red, made from TMS-indole and **3** on heating

Blue made from N-H-indole and **3** on heating

Green, made from TIPS indole and **3** on heating.

Bottom red made from N-H-indoline, **2** and DBP base on heating.

6.0 Computational Data

All calculations were performed using the Gaussian09 programme.[4] Geometry optimisations were completed with the DFT method using the M06-2X functional[5] and the 6-311G(d,p) basis set. All geometry optimizations were full, with no restrictions. Stationary points were characterized as minima by vibrational analysis. Solvent effects of the dichloromethane were introduced using the self consistent field approach, by means of the integral equation formalism polarizable continuum model (IEFPCM).[6]

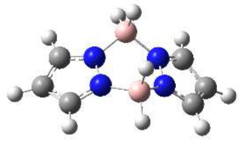

Pyrazabole

N	1.23386600	0.67183500	0.22239100
N	-1.23386600	-0.67183300	0.22240900
C	2.38290700	1.10153200	-0.30571100
C	3.15958000	-0.00003100	-0.65096200
C	2.38300900	-1.10148600	-0.30530300
C	-2.38291700	-1.10153500	-0.30566700
C	-3.15954100	0.00002300	-0.65104300
C	-2.38304100	1.10148400	-0.30524000
H	2.57096700	2.15815100	-0.40745800
H	4.13683000	0.00001100	-1.10089700
H	2.57106700	-2.15815000	-0.40661200
H	-2.57097700	-2.15815600	-0.40739800
H	-4.13675400	-0.00002500	-1.10105700
H	-2.57111800	2.15814700	-0.40652200
N	1.23396700	-0.67169800	0.22272000
N	-1.23396800	0.67170000	0.22272000
B	-0.00002500	-1.44860000	0.79876700
H	0.00017000	-2.57026500	0.37746900
B	0.00002800	1.44860900	0.79874800
H	-0.00017800	2.57027000	0.37743900

H	-0.00021100	-1.35957400	1.99967700
H	0.00021700	1.35959200	1.99965800

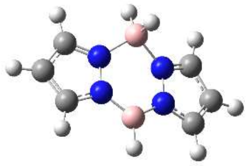

Pyrazabole-monocation

N	1.22664600	0.67482000	-0.14391600
N	-1.24409500	-0.68404100	-0.03774800
C	2.49733700	1.14274600	0.04949700
C	3.32045400	0.07500100	0.27185600
C	2.48320900	-1.05767900	0.20486100
C	-2.48293200	-1.05774100	0.20536300
C	-3.32042900	0.07482000	0.27198800
C	-2.49760900	1.14263100	0.04904200
H	2.69012600	2.20340100	0.03099600
H	4.37829100	0.08966900	0.46634000
H	2.72149400	-2.10491300	0.30969900
H	-2.72100300	-2.10496600	0.31075600
H	-4.37822400	0.08926400	0.46672800
H	-2.69058700	2.20324400	0.03004900
N	1.24421500	-0.68411200	-0.03778600
N	-1.22681000	0.67488200	-0.14452600
B	0.00006500	-1.53999000	-0.47898500
H	0.00003300	-1.60256500	-1.67728200
B	-0.00006800	1.42497700	-0.22253400
H	-0.00011000	-2.58362300	0.09760600
H	0.00012100	2.59604800	-0.29510900

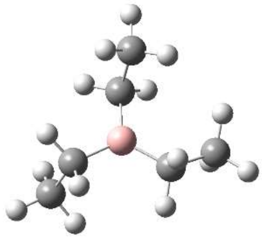

BEt_3

B	-0.17944300	-0.02768800	0.12516200
C	0.43991900	1.15871500	-0.70255500
H	-0.32736500	1.78678600	-1.16613300
H	1.11944500	0.82127900	-1.49091100
C	-1.65698100	0.09275000	0.65787800
H	-1.83840100	-0.52171600	1.54544000
H	-1.91464600	1.12827400	0.90621700
C	0.66389600	-1.30726200	0.48539100
H	0.90779800	-1.18210100	1.55335100
H	0.00875400	-2.18809300	0.47185700
C	1.94373400	-1.57025300	-0.31214000
H	2.48017200	-2.44939000	0.05191100
H	1.71979000	-1.73461300	-1.36932100
H	2.62709300	-0.71866300	-0.25585300
C	-2.58685000	-0.38670700	-0.48091000
H	-3.63676800	-0.32663100	-0.18760800
H	-2.45787900	0.22109100	-1.38040100
H	-2.37835000	-1.42575600	-0.75054500
C	1.23937900	2.01624200	0.30890300
H	1.69985500	2.87760500	-0.17873000
H	0.59213100	2.39394000	1.10522500
H	2.03700900	1.43552000	0.78028200

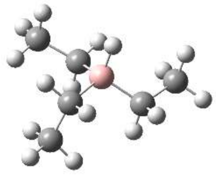

$HBEt_3$

B	0.19453800	0.00676400	0.56755000
C	-0.30335400	1.26465700	-0.37302100
H	-0.39036200	0.93462500	-1.42169800
H	0.45711800	2.05925100	-0.38698400
C	-0.98829000	-1.13291400	0.72084100
H	-0.61327700	-1.97308000	1.32605700
H	-1.84599600	-0.73477500	1.28349100
C	1.56832200	-0.66862800	-0.03853200
H	1.85046400	-1.54982400	0.55944700
H	1.38033000	-1.05715800	-1.05275700
C	2.77397400	0.27760400	-0.10653800
H	3.68490700	-0.19334200	-0.49622700
H	2.55757100	1.13966300	-0.74588500
H	3.00971600	0.67303300	0.88802500
C	-1.51170700	-1.69341900	-0.61015400
H	-2.27802200	-2.46858000	-0.48695400
H	-1.95173800	-0.89883400	-1.22315800
H	-0.69903300	-2.13241200	-1.19839100
C	-1.63939300	1.88539500	0.05252700
H	-1.93104700	2.75962500	-0.54250500
H	-2.45213000	1.15507900	-0.02265800
H	-1.59904400	2.20208100	1.10106100
H	0.45054400	0.45466100	1.71065300

7.0 Crystallographic Data

Crystallographic data for compound **2** were recorded on a Bruker APEX-II CCD at 100 K with Mo Kα radiation (λ = 0.71073 Å). Crystallographic data for compounds **2-(MeCN)₂**, **7H**, **8**, and **9** were recorded on a Rigaku Oxford Diffraction Excalibur diffractometer, at 120 K with Mo Kα radiation (λ = 0.71073 Å) equipped with Eos CCD detector.

For data set **2** Bruker APEX3 software package was used for data collection, the applications SAINT[7] and SADABS[8] were used for the data reduction and absorption corrections of the data, respectively. For data sets **2-(MeCN)$_2$**, **7H**, **8**, and **9** the CrysAlisPro[9] software package was used for data collection, cell refinement and data reduction. The CrysAlisPro software package was used for empirical absorption corrections, which were applied using spherical harmonics, implemented in SCALE3 ABSPACK scaling algorithm. All further data processing was undertaken within the Olex2 software package.[10] The molecular structures of all compounds were solved with the ShelXT[11] structure solution program using Intrinsic Phasing and refined with the ShelXL[12-14] refinement package using Least Squares minimisation. Non-hydrogen atoms were refined anisotropically. Hydrogen atoms were all located in a difference map and repositioned geometrically.

Crystallographic data have been deposited with the Cambridge Crystallographic Data Centre as supplementary publication numbers 2114150 – 2114154. Selected crystallographic data are presented in Table S1 and S2 and full details in cif format can be obtained free of charge from the Cambridge Crystallographic Data Centre via www.ccdc.cam.uk/data_request/cif.

Table S1. Selected crystallographic data for compounds **2**, **2-(MeCN)$_2$** and **7H**.

	**2**	**2-(MeCN)$_2$**	**7-H**
CCDC No	2114150	2114151	2114154
Empirical formula	$C_{10}H_8B_2F_{12}N_6O_8S_4$	$C_{14}H_{14}B_2F_{12}N_8O_8S_4$	$C_{16}H_{16}B_2F_6N_6O_4S_2$
Formula Weight	718.08	800.19	556.09
Temperature (K)	100.0	120.00(5)	120.2(8)
Radiation	0.71073	0.71073	0.71073
Crystal system	Orthorhombic	Triclinic	Monoclinic
Space group	P bca	P -1	P 2_1/c
a (Å)	13.5759(11)	12.0480(3)	13.7735(5)
b (Å)	9.1503(8)	14.2941(4)	8.5800(2)
c (Å)	18.9509(16)	17.5385(5)	24.2584(9)
α(°)	90	96.679(2)	90
β (°)	90	92.373(2)	126.298(6)
γ (°)	90	93.071(2)	90
Cell volume (Å^3)	2354.1(3)	2992.13(14)	2310.47(19)
Z	4	4	4
ρcalc (gcm^{-3})	2.026	1776	1.599
μ (mm^{-1})	0.553	0.447	0.316
F (000)	1424.0	1600.0	1128.0
Crystal size/ mm^3	0.216 x 0.098 x 0.064	0.227 x 0.142 x 0.108	0.223 x 0.185 x 0.163
2θ range for data collection/°	5.242 to 61.022	6.460 to 58.876	6.836 to 58.606

Index ranges	-19 ≤ h ≤ 19;<br>-12 ≤ k ≤ 12;<br>-26 ≤ l ≤ 26	-16 ≤ h ≤ 15;<br>-19 ≤ k ≤ 19;<br>-23 ≤ l ≤ 23	-19 ≤ h ≤ 18;<br>-11 ≤ k ≤ 11;<br>-32 ≤ l ≤ 31
Reflections collected	64735	78895	30679
Independent reflections	3574 [R_{int} = 0.0508; R_{sigma} = 0.0228]	14908 [R_{int} = 0.0428; R_{sigma} = 0.0412]	5739 [R_{int} = 0.0384; R_{sigma} = 0.0384]
Data/restraints/parameters	3574/0/194	14908/90/885	5739/36/337
Goodnes-of-fit-on F^2 (*GOF*)	1.042	1.023	1.026
Final *R* indices [I>2σ(I)]	R_1 = 0.0333, wR_2 = 0.0768	R_1 = 0.0530; wR_2 = 0.1241	R_1 = 0.0493; wR_2 = 0.1132
R indices (all data)	R_1 = 0.0440, wR_2 = 0.0825	R_1 = 0.0795; wR_2 = 0.1391	R_1 = 0.0801; wR_2 = 0.1308
Largest diff. peak and hole (e Å^{-3})	0.44 / -0.58	0.90 / -0.62	0.52 / -0.34

Table S2. Selected crystallographic data for compounds **8** and **9**.

	**8**	**9**
CCDC No	2114153	2114152
Empirical formula	$C_{22}H_{20}B_2N_6$	$C_{17}H_{18}B_2F_6N_6O_4S_2$
Formula Weight	390.06	570.11
Temperature (K)	120.0(2)	120.00(11)
Radiation	0.71073	0.71073
Crystal system	Orthorhombic	Monoclinic
Space group	P bca	P 2_1/n
a (Å)	7.8774(2)	14.4558(4)
b (Å)	22.1348(5)	11.4367(3)
c (Å)	22.9407(6)	15.1421(4)
α(°)	90	90
β (°)	90	109.083(3)
γ (°)	90	90
Cell volume (Å^3)	4000.05(17)	2365.82(12)
Z	8	4
ρcalc (gcm^{-3})	1.295	1.601
μ (mm^{-1})	0.079	0.311
F (000)	1632.0	1160.0
Crystal size/ mm^3	0.416 x 0.223 x 0.208	0.22 x 0.15 x 0.07
2θ range for data collection/°	7.104 to 58.83	6.55 to 59.002
Index ranges	-10 ≤ h ≤ 10;<br>-30 ≤ k ≤ 28;<br>-28 ≤ l ≤ 31	-19 ≤ h ≤ 19;<br>-156 ≤ k ≤ 15;<br>-20 ≤ l ≤ 20
Reflections collected	81826	50857
Independent reflections	5208 [R_{int} = 0.0510; R_{sigma} = 0.0298]	6016 [R_{int} = 0.0364; R_{sigma} = 0.0260]
Data/restraints/parameters	5208/0/279	6016/0/343
Goodnes-of-fit-on F^2 (*GOF*)	1.072	1.034
Final *R* indices [I>2σ(I)]	R_1 = 0.0526; wR_2 = 0.1164	R_1 = 0.0368; wR_2 = 0.0866
R indices (all data)	R_1 = 0.0789; wR_2 = 0.1280	R_1 = 0.0496; wR_2 = 0.0931
Largest diff. peak and hole (e Å^{-3})	0.32 / -0.34	0.35 / -0.47

8.0 References

[1] S. Trofimenko, *J. Am. Chem. Soc.* **1967**, *89*, 3165-3170.

[2] C. J. Smith, M. W. S. Tsang, A. B. Holmes, R. L. Danheiser, J. W. Tester, *Org. Biomol. Chem.* **2005**, *3*, 3767-3781.

[3] P. J. Beswick, C. S. Greenwood, T. J. Mowlem, G. Nechvatal, D. A. Widdowson, *Tetrahedron* **1988**, *44*, 7325-7334.

[4] M. J. Frisch, G. W. Trucks, H. B. Schlegel, G. E. Scuseria, M. A. Robb, J. R. Cheeseman, G. Scalmani, V. Barone, G. A. Petersson, H. Nakatsuji, X. Li, M. Caricato, A. V. Marenich, J. Bloino, B. G. Janesko, R. Gomperts, B. Mennucci, H. P. Hratchian, J. V. Ortiz, A. F. Izmaylov, J. L. Sonnenberg, Williams, F. Ding, F. Lipparini, F. Egidi, J. Goings, B. Peng, A. Petrone, T. Henderson, D. Ranasinghe, V. G. Zakrzewski, J. Gao, N. Rega, G. Zheng, W. Liang, M. Hada, M. Ehara, K. Toyota, R. Fukuda, J. Hasegawa, M. Ishida, T. Nakajima, Y. Honda, O. Kitao, H. Nakai, T. Vreven, K. Throssell, J. A. Montgomery Jr., J. E. Peralta, F. Ogliaro, M. J. Bearpark, J. J. Heyd, E. N. Brothers, K. N. Kudin, V. N. Staroverov, T. A. Keith, R. Kobayashi, J. Normand, K. Raghavachari, A. P. Rendell, J. C. Burant, S. S. Iyengar, J. Tomasi, M. Cossi, J. M. Millam, M. Klene, C. Adamo, R. Cammi, J. W. Ochterski, R. L. Martin, K. Morokuma, O. Farkas, J. B. Foresman, D. J. Fox, Wallingford, CT, **2016**.

[5] Y. Zhao, D. G. Truhlar, *Theor. Chem. Acc.* **2008**, *120*, 215-241.

[6] B. Mennucci, E. Cancès, J. Tomasi, *J. Phys. Chem. B* **1997**, *101*, 10506-10517.

[7] Bruker, *Bruker AXS*, Inc. SAINT, Version 7.68A; Bruker AXS: Madison WI 2009.

[8] G. M. Sheldrick, SADABS, Version 2008/2; University of Göttingen: Germany, 2003.

[9] CrysAlisPro, Agil. Technol. Version 1.1 71.35.19 (release 27-10-2011 CrysAlis171.NET) (compiled Oct 27 2011,150211).

[10] OLEX2: a complete structure solution, refinement and analysis program. O. V. Dolomanov, L. J. Bourhis, R. J. Gildea, J. A. K. Howard, H. Puschmann, *J. Appl. Cryst.* **2009**, *42*, 339 – 341.

[11] *SUPERFLIP*- a computer program for the solution of crystal structures by charge flipping in arbitrary dimensions. L. Palatinus, G. Chapuis, *J. Appl. Cryst.* **2007**, *40*, 786 – 790.

[12] Symmetry determination following structure solution in P1. L. Palatinus, A. Van der Lee, *J. Appl. Cryst.* **2008**, *41*, 975 – 984.

[13] EDMA: a computer program for topological analysis of discrete electron densities. L. Palatinus, S. J. Prathapa, S. Van Smaalen, *J. Appl. Cryst.* **2012**, *45*, 575 – 580.

[14] A short history of SHELX. G. M. Sheldrick, *Acta Cryst.* **2008**, *A64*, 112 – 122.
